# Contribution of the *Bacillus subtilis ytrGABCDEF* operon to antibiotic stress adaptation

**DOI:** 10.1128/spectrum.02694-25

**Published:** 2025-12-05

**Authors:** Luna Baruah, Margareth Sidarta, Pauline Hammer úr Skúoy, Olivia Johnsson, Emma Frisk, Paula Didelot, Aysha Arshad, Michaela Wenzel

**Affiliations:** 1 Division of Chemical Biology, Department of Life Sciences, Chalmers University of Technology 684649, Gothenburg, Sweden; 2 Centre for Antibiotic Resistance Research in Gothenburg (CARe), Gothenburg, Sweden; Rowan University Cooper Medical School, Camden, New Jersey, USA

**Keywords:** antibiotic stress response, *Bacillus subtilis*, ABC transporters, cell wall biosynthesis

## Abstract

The *Bacillus subtilis ytrGABCDEF* operon encodes a putative ABC transporter, its transcriptional repressor YtrA, and YtrG, a small transmembrane protein of unknown function. The operon is induced by inhibitors of the membrane-bound lipid II cycle, cold shock, and during the exponential to stationary phase transition and has been implicated in antibiotic stress adaptation, cell wall synthesis, sporulation, biofilm formation, and competence. Here, we assessed its contribution to antibiotic stress adaptation using a broad range of *ytr* mutant strains and different susceptibility testing setups. We could not confirm a consistent role of the operon in antibiotic survival. However, we observed a stimulating effect of ABC transporter expression on cell wall synthesis and turnover and could pinpoint this to the membrane-bound lipid II cycle. We conclude that the *ytrGABCDEF* operon is induced by antibiotics that target lipid-linked cell wall precursors, not because it constitutes a dedicated antibiotic stress response but rather because these stress conditions mimic its natural trigger. We speculate that this could be related to the accumulation of intracellular cell wall precursor compounds, a signature effect of antibiotics that bind to lipid-linked cell wall precursors such as lipid II or bactoprenol phosphate.

**IMPORTANCE:**

The *Bacillus subtilis ytrGABCDEF* operon is a reliable and specific marker for the inhibition of cell wall biosynthesis by antibiotics. It has therefore advanced as a common reporter in transcriptomic, proteomic, and reporter gene studies aimed at elucidating antibiotic mechanisms of action. Despite this established role, its function is poorly understood, and its contribution to survival under antibiotic exposure is debated. Here, we provide evidence that the function of the operon is not related to antibiotic stress but rather that its induction is a by-product of antibiotic-induced changes in cell wall metabolism.

## INTRODUCTION

*Bacillus subtilis* is a well-characterized Gram-positive model organism, most prominently used for studying bacterial cell division, cell wall synthesis, and sporulation (1–4). Its evolutionary closeness to important Gram-positive pathogens, such as *Bacillus cereus*, *Bacillus anthracis*, *Listeria monocytogenes*, and *Staphylococcus aureus*, has made it a popular non-pathogenic model for studying antibiotic mechanisms and stress responses (5–7). Thus, a *B. subtilis* proteomic profiling library, comprising stress response profiles to around 100 different compounds with antimicrobial properties, constitutes the broadest systematic collection of proteomic stress responses to antibiotics and other antimicrobials (8). These profiles allow the identification of highly specific marker proteins that are indicative of a specific antibacterial mechanism or target and are thus a powerful tool in mode of action analysis (7). Some marker proteins are rather well-characterized, and their functions in antibiotic stress adaptation are clear, for example, upregulation of fatty acid synthases in response to treatment with the fatty acid synthesis inhibitor platensimycin (9). However, some proteins are reliable markers for a specific mechanism; however, their functions are partially or entirely unknown. This is the case for YtrB and YtrE, which are specific and reliable markers for inhibition of cell wall synthesis (10, 11).

**Fig 1:**
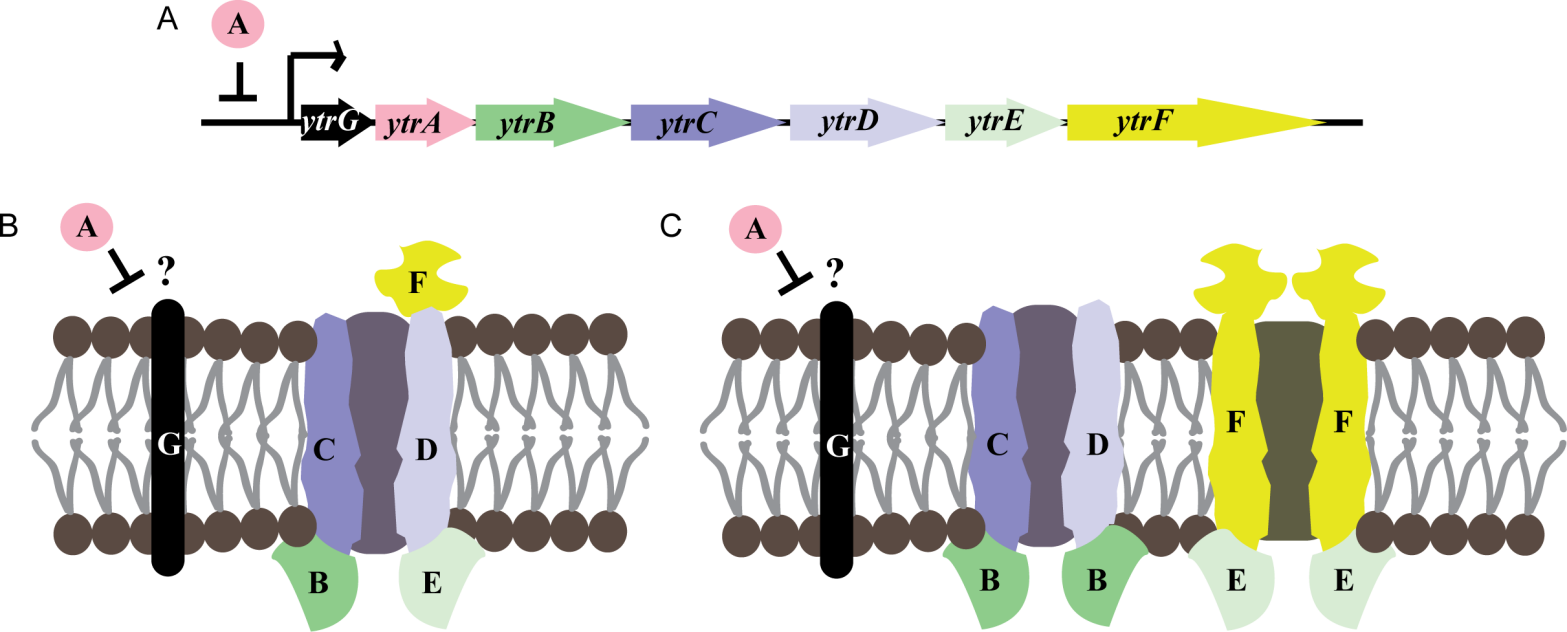
Genetic organization of the *ytr* operon (**A**) and predicted subunits of the encoded ABC transporter according to Yoshida et al. (13) (**B**) and Yu et al. (18) (**C**).

These proteins are part of the *ytrGABCDEF* operon (Fig. 1A). Earlier studies described the operon to encode one single ATP-binding cassette (ABC) transporter YtrBCDEF (Fig. 1B) (12–15). In this model, YtrB and YtrE constitute the ATP-binding subunits, YtrC and YtrD the transmembrane channel proteins, and YtrF an extracytoplasmic substrate-binding lipoprotein. The GntR family transcriptional repressor YtrA is the transcriptional repressor of the operon, and its deletion results in constitutive expression of the ABC transporter (14). Finally, the small transmembrane protein YtrG is part of the operon but is not predicted to be part of the ABC transporter (14, 16). Newer studies have proposed two independent transporters, YtrB_2_CD and YtrE_2_F_2_ (Fig. 1C) based on Alpha Fold structural predictions (17). The YtrE_2_F_2_ structure could indeed be observed experimentally, yet in a system that only expresses the E and F subunits and not the remaining operon (18). The genetic organization and regulation of the operon, lacking any known secondary promoter or posttranscriptional modifications and showing the same regulation patterns for all genes (13, 19), raises the question how a YtrB_2_CD and YtrE_2_F_2_ stoichiometry could be achieved. Future structural and genetic studies will be needed to conclusively unite these differing observations.

A number of 2D gel-based proteomic profiling studies have shown upregulation of YtrB and YtrE after treatment with compounds that interfere with cell wall synthesis (Table S1) (8, 10, 11, 20–23). A similar transcriptomic study found comparable results for operon transcript levels (Table S2) (14). Induction of *ytr* genes in response to cell wall synthesis inhibitors has been confirmed in further independent studies, for example, for vancomycin (3.3-fold to 5.9-fold induction after 3 min of treatment) (24), bacitracin (9.1-fold induction after 5 min of treatment) (25), and plectasin (56-fold after 10 min of treatment) (26). Moreover, the *Pytr* promoter has been used as a reporter for glycopeptides, such as vancomycin (27, 28) and ristocetin (27).

All these studies have consistently found that the *ytr* genes are expressed in response to antibiotics that interfere with the lipid II cycle, more precisely those that bind to lipid-linked cell wall precursors (bactoprenol phosphate/pyrophosphate, lipid I/II). Compounds that interfere with cytosolic or extracellular cell wall synthesis steps or specifically inhibit the involved enzymes do not affect *ytr* expression (Tables S1 and S2) (8, 10, 11, 14, 20–26). Senges et al. proposed that the encoded ABC transporter plays a role related to the lipid II cycle and speculated that it may be involved in resistance against such compounds (11). This speculation was corroborated by the finding that a ∆*ytrA* mutant, constitutively expressing *ytrGABCDEF*, was less sensitive to the acute effects of nisin than the corresponding wild-type strain (11).

In addition to antibiotic induction patterns, further observations have suggested a link between the *ytr* operon and cell wall synthesis (13, 15, 29). Koo et al. observed that a ∆*ytrA* mutant, constitutively expressing the operon, lost its genetic competence (29). Following up on this, Benda et al. found that Δ*ytrA,* as well as other *ytr* mutants that showed reduced competence, had a strongly increased cell wall thickness, potentially hampering the uptake of DNA (15). Additionally, the ∆*ytrA* deletion strain showed altered biofilm morphology and reduced sporulation (13, 15, 29). Both sporulation and biofilm formation are affected by the disruption of cell wall synthesis (15, 30, 31). The *ytr* operon has also been implicated in stationary phase transition, acetoin consumption, and cold shock (13, 19) (see also Fig. S1). Together, these observations suggest that the *ytr* operon plays a role during active cell growth and during adjustment to slow/stalled growth but is not needed for maintenance of non-growing stationary phase cells. This fits with a role in cell wall synthesis and/or homeostasis.

Here, we focused on the role of the *ytr* operon in adaptation to cell wall synthesis inhibition by antibiotics. Although we could not find clear evidence for a decisive role of the *ytr* operon in antibiotic adaptation, we could confirm its involvement in cell wall synthesis and sporulation and found clear temperature-dependent phenotypes.

## RESULTS

### Antibiotic susceptibility of the *ytr* mutant panel

Despite being reliably induced by inhibitors of the lipid II cycle, a clear role of the *ytr* operon in antibiotic resistance could not be established so far. Thus, disc diffusion and minimal inhibitory concentration (MIC) tests using a wide array of cell wall synthesis inhibitors did not result in altered antibiotic susceptibility of a ∆*ytrABCDEF* mutant (14). However, acute shock experiments with ∆*ytrA* showed reduced nisin susceptibility (11), suggesting that phenotypes may be observed in strains constitutively expressing the *ytr* operon, or with acute shock assays. To examine this, we performed susceptibility assays with a range of *ytr* mutants: ∆*ytrA* (PH5), ∆*ytrB* (PH1), ∆*ytrC* (PD3), ∆*ytrD* (PD2), ∆*ytrE* (PH2), ∆*ytrF* (PD1), ∆*ytrAB* (GP3193), ∆*ytrAE* (GP3196), ∆*ytrABE* (GP3206), ∆*ytrACD* (BLMS3), and ∆*ytrGABCDEF* (GP2646). If the operon is of importance for cellular survival under antibiotic stress, we expect a lower susceptibility of the ∆*ytrA* strain and a higher susceptibility of the ∆*ytrGABCDEF* strain. Single mutants of transporter subunits (∆*ytrB*, ∆*ytrC*, ∆*ytrD*, ∆*ytrE*, and ∆*ytrF*) and constitutively expressed incomplete transporter variants (∆*ytrAB*, ∆*ytrAE*, ∆*ytrABE*, and ∆*ytrACD*) were included to assess the importance of individual subunits, if susceptibility changes were found. All susceptibility assays were performed at both 37°C and 24°C to examine the effects of lower temperature.

**Fig 2:**
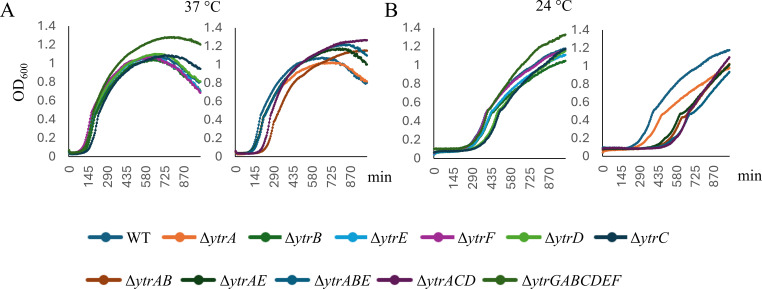
Growth curves of *B. subtilis* 168CA and *ytr* mutants at 37°C (**A**) 24°C (**B**). Left panels show strains not constitutively expressing the operon (∆*ytrB*, ∆*ytrC*, ∆*ytrD*, ∆*ytrE*, ∆*ytrF*, and ∆*ytrGABCDEF*) whereas the right panels show strains that force-express the whole operon or parts thereof (∆*ytrA*, ∆*ytrAB*, ∆*ytrAE*, ∆*ytrABE*, and ∆*ytrACD*). See also Fig. S4 for graphs with log scales.

Our initial antibiotic set consisted of ampicillin, nisin, vancomycin, D-cycloserine, and tetracycline. Based on gene expression data, nisin and vancomycin (binding lipid II) were expected to result in an altered susceptibility phenotype, whereas ampicillin and D-cycloserine (inhibiting extracellular and intracellular steps of cell wall synthesis, respectively) were not. The translation inhibitor tetracycline was included as an unrelated negative control. However, only ampicillin showed the expected pattern in standard microdilution MIC assays at 37°C (Fig. S2; Table S3). Additionally, all strains constitutively expressing incomplete transporter variants (∆*ytrAB*, ∆*ytrAE*, ∆*ytrABE*, and ∆*ytrACD*) showed increased susceptibility to ampicillin. At 24°C, only ∆*ytrAB*, ∆*ytrAE*, and ∆*ytrABE* strains showed mildly but reproducibly increased ampicillin susceptibility (Fig. S3; Table S3). It should be noted that the ∆*ytrAB*, ∆*ytrAE*, ∆*ytrABE*, and ∆*ytrACD* strains show clearly prolonged lag phases at lower temperatures (Fig. 2B). Thus, their increased susceptibility is likely an unspecific consequence of generally reduced fitness. For nisin, the ∆*ytrGABCDEF* mutant was more resistant at 37°C, whereas ∆*ytrA* was considerably more susceptible than the wild-type at 24°C. This pattern was the opposite of what was expected based on previous results (11), and the remaining strains showed no cohesive pattern. D-cycloserine was less effective against ∆*ytrC*, ∆*ytrD*, and ∆*ytrE* at 37°C, but less effective against ∆*ytrABE* and ∆*ytrACD* at 24°C, likewise not allowing clear conclusions. No reproducible MIC changes were observed for vancomycin and tetracycline at either temperature.

**Fig 3:**
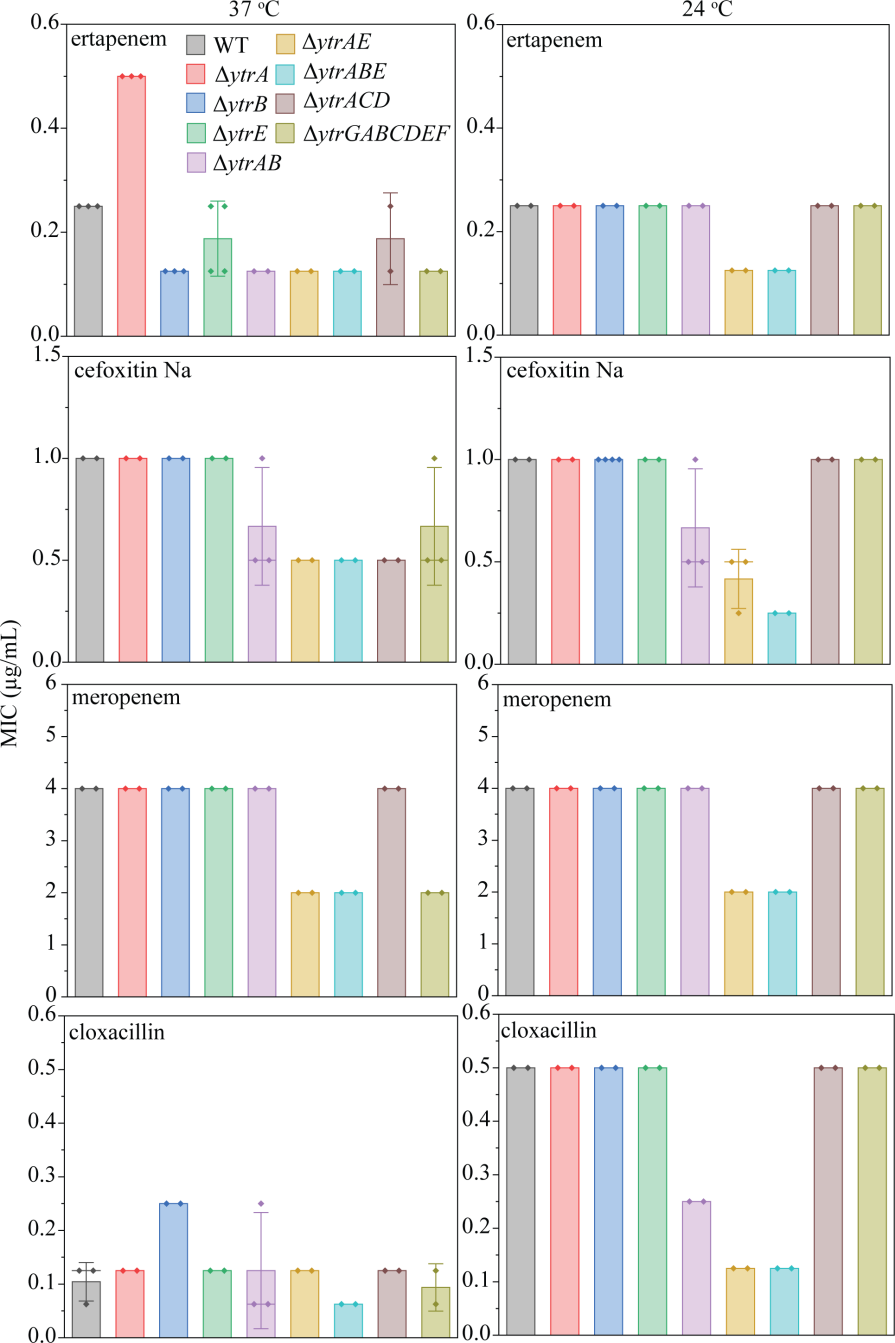
MICs of different β-lactam antibiotics against *ytr* deletion mutants at 37°C and 24°C. Error bars show the standard deviation of the mean.

Since ampicillin was the only antibiotic that produced the expected pattern for ∆*ytrA* and ∆*ytrGABCDEF,* we decided to test additional β-lactam antibiotics to examine whether, against the notion of the gene expression data, the *ytr* operon may protect against this antibiotic class. Thus, our second test set included ertapenem, cefoxitin, meropenem, and cloxacillin (Fig. 3; Table S4). Only for ertapenem did we find a lower susceptibility of ∆*ytrA* and a higher susceptibility of ∆*ytrGABCDEF*, yet only at 37°C. All other antibiotics showed a tendency to be more active against ∆*ytrAB*, ∆*ytrAE*, ∆*ytrABE*, ∆*ytrACD*, likely due to the compromised fitness of these strains.

### Mutant susceptibility to acute antibiotic shock

Since Senges et al. observed lower nisin susceptibility of ∆*ytrA* under acute antibiotic stress, we decided to perform acute shock experiments with the three antibiotics that showed clear MIC differences, ampicillin, ertapenem, and nisin. To this end, cultures were grown until the exponential growth phase and subsequently treated with different concentrations of antibiotic (Fig. S5 to S10). In contrast to endpoint optical density (OD) measurements used in MIC assays, acute shock assays permit assessment of the extent of growth inhibition over time, allowing the detection of immediate, subtle, and transient effects that go unnoticed in MIC assays (32). Here, we took into account the degree of initial cell lysis, length of the lag phase before recovery from initial inhibition/lysis, and maximum OD, comparing each mutant with the WT under the same conditions. Indeed, applying these criteria, several conditions that appeared indifferent in the MIC showed sensitive or tolerant phenotypes in these experiments (Table 1). However, we still could not reproduce the lower nisin susceptibility phenotype of the ∆*ytrA* mutant (11).

**TABLE 1:** Summary of antibiotic susceptibility assays for ampicillin (Amp), nisin (Nis), and ertapenem (Ert)^*a*^

	37°C						24°C					
	Amp		Nis		Ert		Amp		Nis		Ert	
	MIC	AS	MIC	AS	MIC	AS	MIC	AS	MIC	AS	MIC	AS
∆*ytrA*	T	S	I	S	T	T	I	I	S	S	I	I
∆*ytrB*	I	T	T	S	S	T	I	I	I	S	I	I
∆*ytrC*	I	S	I	T	nd	S	I	I	T	T	nd	S
∆*ytrD*	I	I	I	S	nd	I	I	T	T	T	nd	I
∆*ytrE*	I	T	I	S	S	I	I	T	S	S	I	I
∆*ytrF*	I	I	I	I	nd	I	I	T	I	I	nd	I
∆*ytrAB*	S	S	I	T	S	S	S	T	T	T	I	S
∆*ytrAE*	S	S	I	S	S	S	S	I	I	S	S	S
∆*ytrABE*	S	S	I	S	S	S	S	S	I	S	S	S
∆*ytrACD*	S	S	T	T	I	S	I	T	T	T	I	S
∆*ytrGABCDEF*	S	T	T	S	S	I	I	T	I	S	I	S

^*a*^ Sensitivity/tolerance in acute shock experiments was determined based on the extent of growth inhibition and length of lag phase in cultures able to recover from initial inhibition/lysis. The more sensitive (S) or more tolerant (T) strains are indicated as follows: more sensitive: S, more tolerant: T, indifferent/no reproducible result: I, nd: not determined. MIC: minimal inhibitory concentration, AS: acute shock.

### Effects of antibiotics on YtrD localization

To test the behavior of the Ytr transporter under antibiotic stress, we constructed a strain expressing a xylose-inducible YtrD fusion to monomeric superfolder green-fluorescent protein (msfGFP, strain MS42) as a proxy for the whole ABC transporter. As expected, YtrD showed a clear ubiquitous membrane localization in small clusters, a characteristic pattern for many transmembrane proteins (33). This localization pattern did not change with temperature (Fig. S11). Hence, we performed antibiotic experiments at 37°C. To test the effects of antibiotic stress on YtrD localization, we selected ampicillin, nisin, vancomycin, and D-cycloserine as cell wall synthesis inhibitors, the proton ionophore carbonyl cyanide m-chlorophenyl hydrazone (CCCP) as a control for membrane depolarization, and the protein synthesis inhibitors erythromycin and tetracycline as unrelated controls (Fig. S12). Vancomycin, erythromycin, and tetracycline had no major effects on YtrD localization. CCCP induced accumulation of the protein in characteristic clusters that typically occur as a consequence of a dysregulation of fluid lipid domains due to membrane depolarization (34, 35). Accordingly, the same domains were induced by nisin, which forms transmembrane pores after docking to lipid II (36). Additionally, nisin and ampicillin induced large membrane domains, from which YtrD was excluded. Such domains usually represent gel phase membranes that occur in lysing cells (37). D-cycloserine caused the accumulation of YtrD in bright membrane patches as well as delocalization of the protein into the cytosol, yet only at a time point where cell lysis had already set in, suggesting that these phenotypes are secondary effects. Thus, YtrD acted like most membrane proteins, being attracted to fluid and excluded from rigid membrane domains, and neither its normal localization nor its behavior under antibiotic stress pointed toward a specific recruitment under antibiotic stress, as can be observed with cell envelope stress proteins (38). It should be noted that we also attempted to localize YtrE to account for the possibility of a YtrE_2_F_2_ transporter, yet did not observe any membrane localization of a YtrE-msfGFP fusion (strain PH4) (Fig. S11).

### Possible role of the *ytrGABCDEF* operon in cell wall homeostasis

Despite varying results, our antibiotic susceptibility assays generally support the notion that the function of the *ytrGABCDEF* operon is connected to cell wall synthesis. However, if the operon was involved in adaptation to antibiotic stress, we would have expected to find more consistent susceptibility changes that correlate better with the gene expression data, that is, lower susceptibility of ∆*ytrA* to antibiotics that target lipid-linked cell wall precursors (vancomycin and nisin), and higher susceptibility of ∆*ytrGABCDEF*. This was not the case, leading us to the conclusion that the *ytr* operon is not induced as part of a protective stress response against these antibiotics. Rather, we propose that treatment with these compounds mimics conditions that would normally induce the operon. Outside of antibiotics, the operon is induced by cold shock and transition to the stationary growth phase (13, 19, 39). Both conditions impact cell wall homeostasis due to an adaptation to slower growth and an induction of autolysis. Additionally, both cold shock and certain lipid II-binding antibiotics impact the fluidity of the cell membrane (21, 40). However, a role of the operon in regulating membrane fluidity was dismissed since laurdan-based fluidity measurements did not reveal any significant differences between the mutants and the wild-type (Fig. S13).

**Fig 4:**
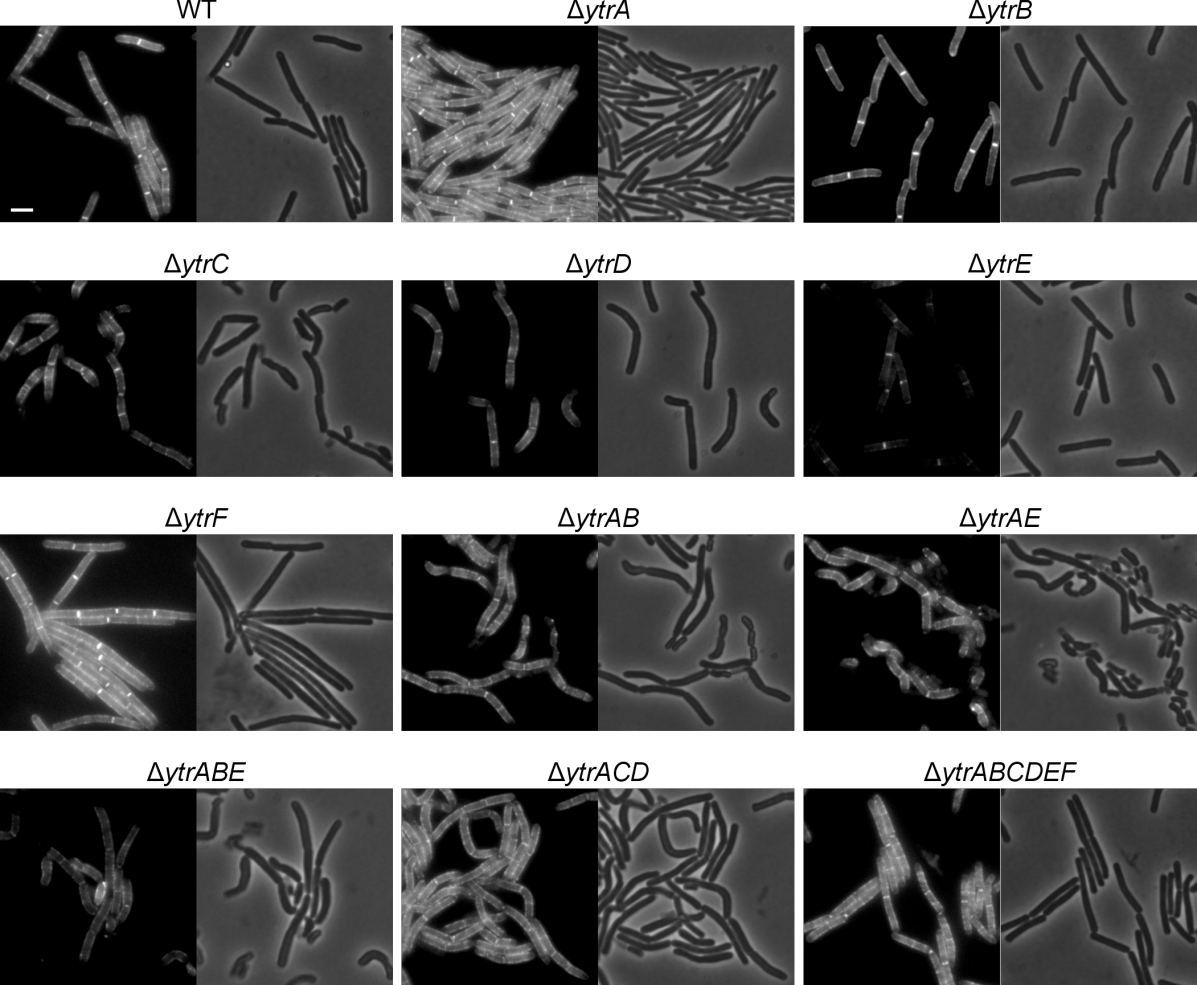
Microscopy images of *B. subtilis* 168CA (WT) and *ytr* mutants labeled with Van-FL. Cells were grown at 24°C. Exposure times, light intensity, and brightness/contrast settings were identical for all samples. Scale bar 2 µm. See Fig. S13 for the corresponding 37°C experiment.

To further probe the connection between the *ytr* operon and cell wall homeostasis, we characterized different *ytrGABCDEF* deletion mutants with respect to their cell wall synthesis and turnover phenotypes. First, we stained the different strains with BODIPY FL vancomycin (Van-FL), a fluorescent vancomycin derivative that allows the visualization of lipid II and thus indicates the localization of active cell wall synthesis. Fluorescence microscopy revealed distinct phenotypes of the different mutant strains (Fig. 4; Fig. S14). In exponentially growing wild-type cells, a characteristic pattern can be observed (41). These cells show a strong signal at mid-cell, where the cross-wall is synthesized during septation, and a less intense, spotty signal along the lateral axis of the cell, where the elongation machinery is active. No staining was observed at the cell poles, where no cell wall synthesis takes place. This pattern was similar at 37 and 24°C, with a slightly stronger septal signal at 37°C (Fig. 4; Fig. S14). While the ∆*ytrA* and ∆*ytrGABCDEF* mutants did not considerably differ from this pattern, expression of an incomplete transporter, either by deletion of *ytrA* (∆*ytrAB*, ∆*ytrAE*, ∆*ytrABE*, and ∆*ytrACD*) or by growth at low temperature, led to a decrease of the septal or overall fluorescence signal, with the clearest effects being observed for ∆*ytrAB*, ∆*ytrAE*, ∆*ytrABE*, and ∆*ytrACD* at 24°C (Fig. 4).

These observations were supported by quantification of whole-cell fluorescence, indicative of the amount of Van-FL binding to available lipid II and thus an estimate for the relative amount of lipid II in the cell membrane (Fig. S15). Intriguingly, at 37°C, the Van-FL signal was higher in ∆*ytrA* and lower in ∆*ytrGABCDEF* (Fig. S14 and S15), suggesting that the presence of the operon stimulates cell wall synthesis, an observation matching the thicker cell wall phenotype observed for ∆*ytrA* by Benda et al. (15). However, it must be considered that the increased cell wall thickness of certain mutants (shown to be the case for ∆*ytrA*, ∆*ytrAB*, and ∆*ytrAE* but not ∆*ytrGABCDEF*) (15) may affect the dye accessibility of membrane-bound lipid II, limiting the reliability of this quantification. Therefore, we additionally performed line scans across the lateral cell axis, visualizing the subcellular distribution of Van-FL, indicative of its accumulation at mid-cell (Fig. S16 to Fig. S39, see also schematic in Fig. S16 for a visualization of how line scans were drawn). Line scans clearly showed the reduction or loss of septal signal, in particular the ∆*ytrAB*, ∆*ytrAE*, ∆*ytrABE*, and ∆*ytrACD* strains grown at 24°C. These results corroborated a connection between the *ytr* operon and the lipid II cycle.

Next, we stained the same set of strains with BODIPY FL penicillin (bocillin), a fluorescently labeled penicillin that binds to penicillin-binding proteins (PBPs), allowing their visualization without the need for expressing fluorescent fusion proteins. In exponentially growing wild-type cells, bocillin results in a mostly septal staining pattern with weak lateral staining (Fig. S40 and S41), reminiscent of the localization pattern of fluorescent protein fusions to PbpB (PBP 2B) and PonA (PBP 1A/1B) (42). Slight aberrations of this pattern were observed in the ∆*ytrAB*, ∆*ytrAE*, ∆*ytrABE*, and ∆*ytrACD* strains, which showed small bocillin clusters at the cell membrane in addition to the regular septal stain. However, no major disruption of PBP localization was observed, supporting the expression data-derived notion that the *ytr* operon is linked to lipid II and bactoprenol phosphate derivatives.

We then employed a cell wall integrity assay, commonly referred to as “bubble assay,” which is based on an acetic acid/methanol fixation protocol. This fixation causes the protoplast to protrude through breaches in the cell wall, appearing as “bubbles” on the cell surface. This effect is strongly exacerbated by impaired cell wall synthesis as the continued activity of autolysins creates gaps in the peptidoglycan sacculus that cannot be filled due to the lack of available lipid II (10, 26, 43–45). Although the high variation between replicates did not allow us to obtain statistically significant results, there was a reproducible tendency for ∆*ytrC*, ∆*ytrD*, ∆*ytrE*, ∆*ytrAB*, ∆*ytrAE*, ∆*ytrABE*, ∆*ytrACD,* and ∆*ytrGABCDEF* to display more bubbles than their corresponding wild-type control at 24°C (up to 2.5-fold, see Fig. S42).

### Effects on cold-induced autolysis

**Fig 5:**
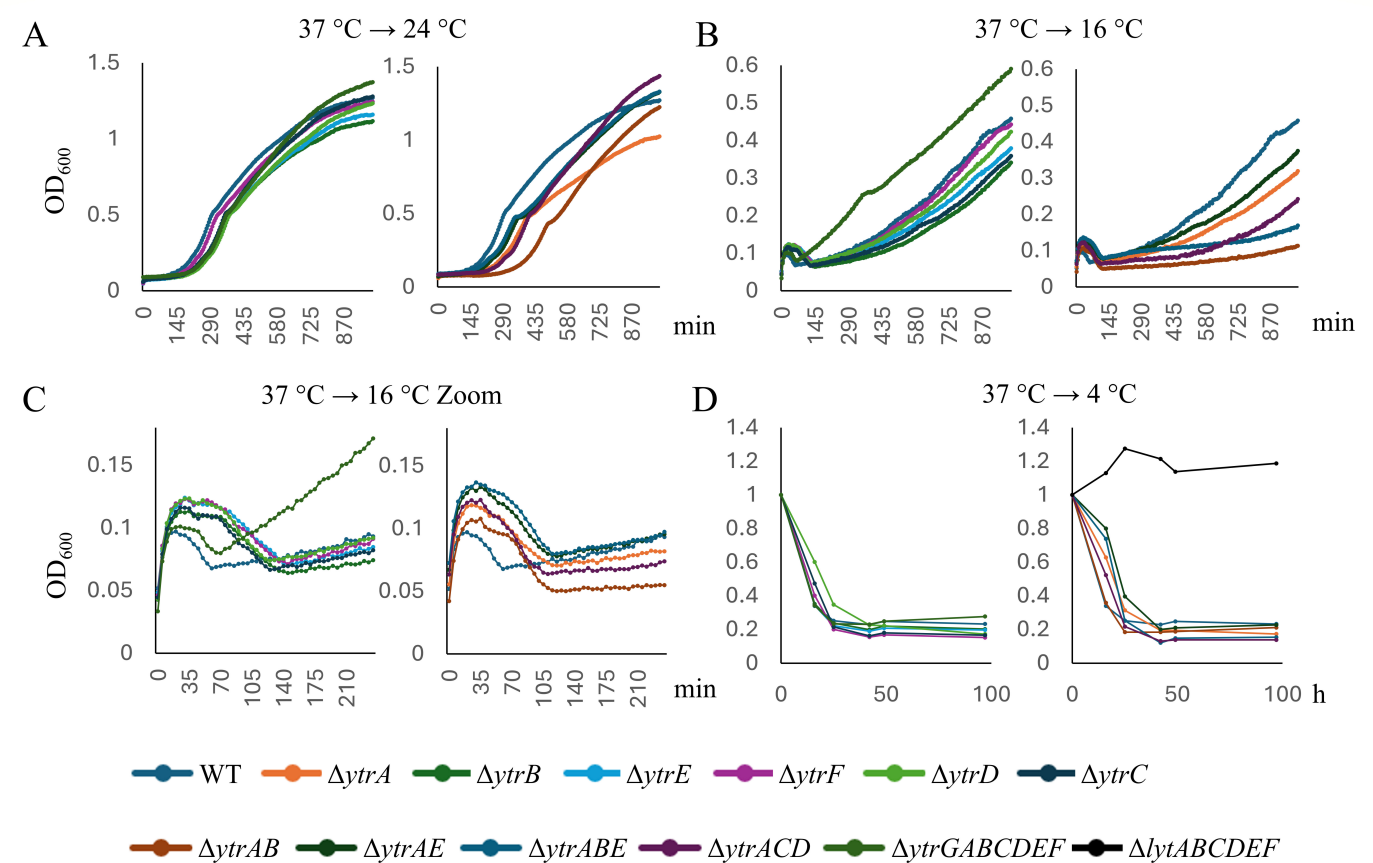
Growth curves of *B. subtilis* 168CA and *ytr* mutants after temperature shifts. Cultures were shifted from 37°C to 24°C (**A**), 16°C (**B and C**), or 4°C (**D**) after dilution of overnight cultures. For the 4°C experiment in (**D**), the autolysin-defective ∆*lytABCDEF* mutant was included as a negative control. Left panels show strains not constitutively expressing the operon (∆*ytrB*, ∆*ytrC*, ∆*ytrD*, ∆*ytrE*, ∆*ytrF*, and ∆*ytrGABCDEF*), whereas the right panels show strains that force-express the whole operon or parts thereof (∆*ytrA*, ∆*ytrAB*, ∆*ytrAE*, ∆*ytrABE*, and ∆*ytrACD*) and, in (**D**), the ∆*lytABCDEF* control. See Fig. S43 for graphs on a log scale.

Cell wall synthesis and autolysis are intimately linked, and autolysis in *B. subtilis* is induced by cold shock. Furthermore, YtrF belongs to the same protein family as FtsX, which regulates the major autolysin CwlO (15). Therefore, we decided to perform growth experiments at different temperatures to assess a possible role of the *ytr* operon in the regulation of cold-induced autolysis. Figure 2 shows growth curves of the different *B. subtilis* strains grown at constant 37°C and 24°C, and Fig. 5 of cultures shifted from 37°C to 24°C, 16°C, and 4°C, respectively. Under all conditions, mutants that constitutively expressed the whole or incomplete operon (∆*ytrA*, ∆*ytrAB*, ∆*ytrAE*, ∆*ytrABE*, and ∆*ytrACD*) showed the strongest growth defects, whereas the whole operon mutant (∆*ytrGABCDEF*) reached higher end ODs than the wild-type (Fig. 2 and 5). When shifted to 16°C, cultures underwent initial cell lysis but were able to resume growth eventually (Fig. 5B and C). Surprisingly, the whole operon mutant overcame this initial cell lysis faster and grew considerably better than all other strains, including the wild-type. All other mutants showed a clear delay in resuming growth and grew considerably slower and to lower ODs than the wild-type. While ∆*ytrF* was the least affected, ∆*ytrA*, ∆*ytrAB*, ∆*ytrABE*, and ∆*ytrACD* showed the most severe growth defects. When shifted to 4°C, which induces autolysis and does not allow cultures to resume growth, the ∆*ytrGABCDEF* mutants no longer exhibited a fitness advantage (Fig. 5D). Slightly faster lysis was observed for ∆*ytrA*, ∆*ytrAB*, ∆*ytrABE*, and ∆*ytrACD*. Taken together, these results suggest that the *ytr* operon affects cell lysis and resumed growth of surviving cells after partial lysis of the population. The fact that ∆*ytrA* showed increased lysis, lower fitness, and reduced capacity for regrowth, whereas the whole operon mutant generally showed the opposite effect, suggests that expression of the *ytr* operon promotes rather than prevents cell lysis. Together with the Van-FL results and the previously published TEM data (15), these results point towards increased cell wall synthesis and turnover in cells that constitutively express the *ytr* operon.

### Effects on motility and sporulation

**Fig 6:**
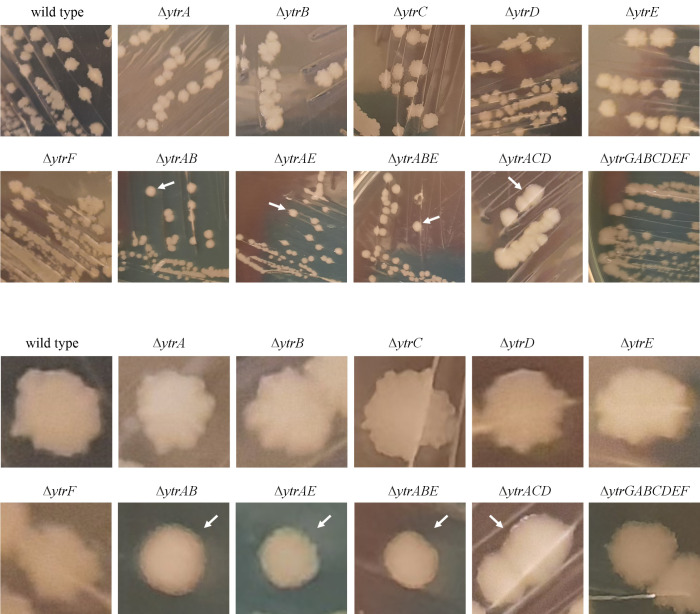
Colony morphology of *ytr* deletion mutants. Lower panels show zoom ins of upper panels. Arrows indicate strains with smooth colony borders.

Interestingly, *ytr* mutants exhibit clear phenotypes with respect to several cellular differentiation processes with bimodal regulation patterns. This includes the competence defects and altered biofilm morphology observed by Benda et al. (15), as well as autolysis. Additionally, we observed that the ∆*ytrAB*, ∆*ytrAE*, ∆*ytrABE*, and ∆*ytrACD* strains produced colonies with smooth boundaries, whereas wild-type cells and all other mutants displayed the “rough” colony boundaries that are characteristic of *B. subtilis* and indicative of swarming microcolonies (46–49), showing that *ytr* also affects swarming motility (Fig. 6).

Furthermore, lower sporulation efficiencies have been found for ∆*ytrA* (29), as well as for mutants that do not express *ytrF* or the whole operon (13). However, the results for *ytrF::pMUTIN2 (*13) could not be confirmed with ∆*ytrF::ery* or ∆*ytrF::kan* deletion mutants (29), and when we ran preliminary sporulation efficiency tests, we could also not confirm the previous findings for ∆*ytrA* and *∆ytrGABCDEF*. Therefore, we decided to assess the sporulation behavior of these strains in more detail.

To this end, we followed asymmetric septation of the ∆*ytrA* and ∆*ytrGABCDEF* mutants over time using fluorescence microscopy. The wild-type and the sporulation-deficient ∆*spoIIIE* mutant (PG344) (50) were included as controls. Sporulation was induced by glucose limitation, and membranes were stained with the non-toxic membrane dye FM4-64 from the onset of glucose limitation. Microscopy samples were taken in hourly intervals from 0 to 10 h, as well as after 24 h, after resuspension in glucose-free medium. Sporulation stages were divided into asymmetric septation, engulfment, forespores, and phase-bright spores (51).

**Fig 7:**
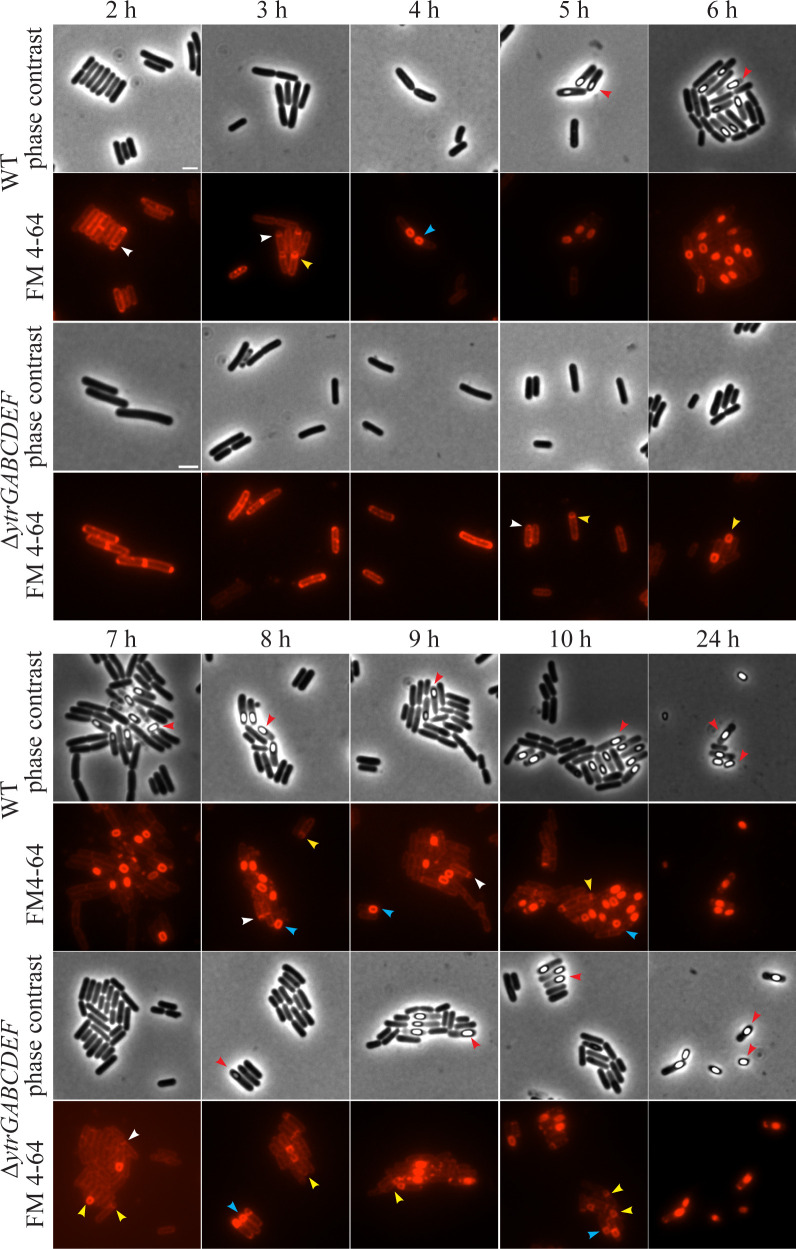
Fluorescence and phase contrast microscopy images of sporulating *B. subtilis* 168CA (WT) and GP2646 (∆*ytrGABCDEF*). Cells were taken for microscopy in hourly intervals from 2 to 10 h and at 24 h after sporulation induction. The experiment was conducted at 37°C. The sporulation stage was determined based on FM4-64 membrane staining and phase contrast images. Arrows indicate different stages of sporulation: white: asymmetric septation, yellow: engulfment, blue: forespore stage, red: phase-bright spores. Scale bar 2 µm.

All strains were still in the vegetative pre-septation stage at the 1 h time point (data not shown). The wild-type and *ΔytrA* mutant formed asymmetric septa after 2 h and initiated the engulfment process, indicated by curved septa, after 3 h (Fig. 7; Fig. S44). The sporulation process continued to the forespore stage and started to show phase-bright spores from the 5 h time point onward. The ∆*ytrGABCDEF* mutant displayed clearly delayed sporulation with asymmetric septa forming at the 5 h time point and phase-bright spores appearing after 7 h (Fig. 7). As expected, the *ΔspoIIE* strain, lacking a gene required for asymmetric septation (52), failed to sporulate and underwent cell lysis instead (Fig. S45). After 24 h, the wild-type, ∆*ytrA,* and ∆*ytrGABCDEF* had successfully sporulated, indicating that sporulation is not defective but merely delayed in the whole operon deletion strain. This was supported by the assessment of sporulation efficiency by heat treatment after 24 h, which showed similar values for these three strains (Fig. S46).

## DISCUSSION

It has been well-established that the *ytr* operon is induced by compounds that impair cell wall synthesis (8, 10, 11, 14, 20–26). Thereby, induction correlates with impairment of the membrane-bound lipid II cycle, that is, the conversion of lipid I to lipid II as well as lipid carrier recycling by dephosphorylation of bactoprenol pyrophosphate to bactoprenol phosphate (Tables S1 and S2). Despite this consensus, previous studies could not find an increased antibiotic sensitivity of *ytr* operon deletion strains (14). However, a ∆*ytrA* deletion mutant that constitutively expresses the operon showed decreased nisin sensitivity in acute shock experiments (11). Despite these conflicting observations, it has been proposed that the *ytrGABCDEF* operon could be involved in antibiotic detoxification (11, 15). Here, we could not confirm this hypothesis. Although we did find differences in the antibiotic sensitivity of diverse *ytr* mutants in both MICs and acute shock assays, the observed effects did not correlate with operon induction patterns, antibiotic targets, or antibiotic class, and were mostly in the range of only 2-fold concentration changes. We could also not observe any evidence for a specific role of the YtrBCDEF (or alternatively YtrB_2_CD) transporter under antibiotic stress based on fluorescence microscopy, as YtrD-msfGFP did not show any cell wall synthesis or stress-specific localization pattern, as known for some cell wall synthesis enzymes and related stress response proteins (21, 38).

From our and others’ results, we conclude that instead of being induced as a specific antibiotic stress response, the inhibition of the lipid II cycle, in particular, binding to lipid II or bactoprenol, results in the same cellular signal that would naturally induce the *ytr* operon. One possibility could be a change in membrane fluidity, as all three triggers known for *ytr* induction, namely antibiotics, temperature, and growth phase, impact this parameter. However, YtrA belongs to the GntR family of transcriptional regulators, which is known to bind small molecule inducers (53, 54), making a metabolic regulation more likely. An early hypothesis was that acetoin could be the inducer molecule, but that was not the case (13). It is tempting to speculate that induction could be related to the accumulation of intracellular cell wall precursors, since the accumulation of uridine diphosphate N-acetylmuramic acid (UDP-MurNac) pentapeptide is a signature effect of lipid II-binding antibiotics (23). However, this speculation needs to be assessed in future studies.

It should be noted that YtrA also controls the expression of the *ywoBCD* operon, which has not been studied here. This operon encodes for a membrane protein of unknown function, a hydrolase, and a major facilitator superfamily transporter (14). Both *ytrGABCDEF* and *ywoBCD* operons are induced in the presence of cell wall synthesis inhibitors (10, 14, 25). However, deletion of the *ywo* operon as well as deletion of both operons also did not alter antibiotic susceptibility (14).

Another hypothesis was that the *ytrGABCDEF* operon could be involved in the uptake of a cell wall precursor or a molecule that could regulate or otherwise affect cell wall synthesis (15). Constitutive expression of the operon in a ∆*ytrA* deletion mutant led to considerably thicker cell walls, supporting an involvement of the transporter(s) in cell wall synthesis. However, the same was observed for ∆*ytrAB* and ∆*ytrAE* strains, expressing non-functional transporter variants that lack one of the ATP-binding subunits, essentially ruling out the explanation that an active transport process is responsible for this phenotype (15). However, it is conceivable that only one ATP-binding subunit is sufficient for the transporter to function or that the protein complex does not act as a transporter at all.

Our results confirmed an involvement of the *ytr* operon in cell wall synthesis. Using Van-FL, we could observe decreased labeling in the whole operon mutant, suggesting that less lipid II is present on the membrane surface. In line, the opposite was observed with the ∆*ytrA* mutant. Furthermore, we could see a clear decrease in septal lipid II accumulation in mutants expressing a non-functional transporter, suggesting reduced cell division activity. No major changes were observed in the localization of PBPs, supporting our notion that the *ytrGABCDEF* operon is connected to the membrane-bound bactoprenol cycle. Although not statistically significant, the tendency of *ytr* mutants to show more cell wall breaches in the bubble assay provides additional support, since this method is most sensitive for antibiotics that target a membrane-bound step of lipid II synthesis (10, 26). The assay is also strongly dependent on autolysin activity, and an autolysin-defective mutant does not produce bubbles. Hence, increased bubbles observed at 24°C could be due to either reduced lipid II synthesis, which would align well with the Van-FL results, or increased autolysin activity.

*B. subtilis* undergoes extensive autolysis at cold temperatures (Fig. 5) (55), and the *ytr* operon is known to be induced by cold shock (19, 39). Furthermore, the ∆*ytrA* mutant displayed reduced fitness at 16°C (29). Our results confirm this observation. Almost all mutants displayed some degree of growth defect at lower temperatures, especially when shifted from 37°C. Thereby, the single mutants ∆*ytrB*, ∆*ytrC*, ∆*ytrD*, and ∆*ytrE* displayed mild defects, whereas the constitutive mutants ∆*ytrA*, ∆*ytrAB*, ∆*ytrAE*, ∆*ytrABE*, and ∆*ytrACD* were much more strongly impaired. These observations could have many reasons, but both a weakened peptidoglycan layer or increased autolysin activity in these mutants would be viable explanations for increased cell lysis and slower growth rates when adapting to lower temperature. If YtrF would fulfill an autolysin-regulating function, as hypothesized based on its similarity to FtsX (15), we would have expected stronger phenotypes of the ∆*ytrF* mutant. In fact, we did not observe a clear phenotype of this strain in any of our assays. Similarly, neither deletion nor overexpression of *ytrF* affected competence (15). Interestingly, the ∆*ytrGABCDEF* strains grew considerably better than the wild-type, especially after a harsh temperature shift from 37°C to 16°C. This observation may point to a distinct, yet so far unknown function of the *ytrG* gene.

Unexpectedly, the ∆*ytrGABCDEF* mutant showed delayed sporulation, suggesting that the operon promotes sporulation rather than inhibits it. However, there were no differences in sporulation efficiency after 24 h. These results differed from previous studies that showed decreased sporulation efficiency in ∆*ytrA* and a pMUTIN2 insertion in the promoter region that does not express the operon (13, 29). Sporulation is a highly medium-dependent process, and it is possible that these differences are due to different media and sporulation protocols, or even varying strain backgrounds (56–58). More extensive studies will be needed to assess the effects of the *ytr* operon on sporulation and explain the conflicting observations made by different groups.

In conclusion, we could not confirm a role of the *ytr* operon in antibiotic stress adaptation. We did, however, provide additional evidence for its involvement in the lipid II cycle and a role in promoting both cell wall synthesis and turnover. Based on our observations, we propose that antibiotics that interfere with lipid-linked cell wall precursors cause a cellular state that triggers de-repression of the *ytrGABCDEF* operon, speculatively the accumulation of an intracellular peptidoglycan precursor molecule such as UDP-MurNac.

## MATERIALS AND METHODS

### Antibiotics

Cefoxitin, cloxacillin, ertapenem, and meropenem were purchased from Sigma Aldrich; ampicillin, erythromycin, and kanamycin from Fisher Bioreagents; vancomycin, D-cycloserine, and tetracycline from Fisher Scientific; nisin from MP Biomedicals; CCCP from Alfa Aesar; spectinomycin from Duchefa Biochemie; and daptomycin from Abcam. Meropenem, tetracycline, and CCCP were dissolved in sterile DMSO, ampicillin, ertapenem, cefoxitin, cloxacillin, D-cycloserine, nisin, spectinomycin, kanamycin, daptomycin, and vancomycin in sterile water, and erythromycin in sterile ethanol. Stock concentrations were prepared at 100 mM (CCCP), 0.25 mg/mL (nisin), 50 mg/mL (tetracycline, kanamycin), 20 mg/mL (erythromycin), 1 mg/mL (meropenem), or 10 mg/mL (all other antibiotics). All antibiotic stock solutions were stored at −20°C until further use.

### Strain construction

Strains, plasmids, and primers used in this study are listed in Tables S5 and S6. *Escherichia coli* strains used as cloning hosts were grown in Luria-Bertani (LB) medium or on LB agar supplemented with 0.5% (wt/vol) glucose at 37°C. *B. subtilis* strains were grown at 37°C in Spizizen minimal medium (SMM) (59), LB medium, or on LB agar. Where appropriate, 100 µg/mL ampicillin, 7.5 µg/mL kanamycin, 2 µg/mL erythromycin, or 100 µg/mL spectinomycin was added for selection.

#### Plasmid construction

Plasmids pMS33 (*Pxyl-ytrD-msfgfp*) and pPH2 (*Pxyl-ytrE-msfgfp*) were designed in SnapGene 6.2 and constructed with Gibson assembly (60) (GeneArt Gibson Assembly HiFi Kit, Invitrogen). The *ytrD* and *ytrE* gene sequences were obtained from the SubtiWiki database (16). The *ytrD* genes were amplified from *B. subtilis* 168CA (DSM 402) chromosomal DNA, isolated by standard phenol-chloroform extraction (61), using the primer pair MSP152/MSP153 for *ytrD* and PHP14/PHP15 for *ytrE*. The pMW1 (21) plasmid backbone was linearized by PCR using primers MWP1 and Abs1. After 1 h of DpnI treatment at 37°C and subsequent purification, Gibson assembly was performed using a 1:1 vector to insert ratio (0.08 pmol), resulting in plasmids pMS33 and pPH2, which were then transformed into chemically competent *E. coli* cells (62). The plasmids were isolated and verified by sequencing (Eurofins MWG) using the sequencing primers TerS21 and Abs5.

#### Construction of *B. subtilis* strains

Strains were constructed by transforming either plasmid or chromosomal DNA into *B. subtilis* 168CA as indicated in Table S5. *B. subtilis* was transformed according to a standard starvation protocol (40). All strains were confirmed by PCR (see Table S6 for PCR primers).

### Experimental growth conditions

With the exception of sporulation experiments, in which Belitzky minimal medium (BMM) was used, strains were grown in LB medium throughout the study. Unless otherwise noted, experiments were performed at both 37°C and 24°C, and cultures were kept under continuous agitation to ensure optimal oxygen supply.

### Minimal inhibitory concentrations (MIC)

MICs against all strains were determined in a standard microdilution assay (63), following the guidelines issued by the Clinical Laboratory Standardization Institute (CLSI) (64), with the only exception of using LB as culture medium. All cultures were incubated for 16 h prior to reading out the results. The lowest antibiotic concentration inhibiting visible growth was defined as MIC.

### Acute antibiotic shock experiments

*B. subtilis* overnight cultures were diluted to an OD_600_ of ~ 0.05, and all strains were grown until the exponential growth phase (OD_600_ 0.4–1). Subsequently, cultures were adjusted to an OD_600_ of 0.3, followed by splitting of the cultures and addition of antibiotics as specified in the respective figure legends. Growth was followed by optical density readings in 5 min intervals. Measurements were recorded using a BMG Clariostar Plus plate reader and were continued until stationary phase. For each condition, every strain was compared with the WT with respect to the degree of initial cell lysis, length of the lag phase before recovery from initial inhibition/lysis, and maximum OD. Differences reproducible across all biological replicates were categorized as sensitive (S) or tolerant (T), whereas all samples without growth differences or irreproducible differences were categorized as indifferent (I).

### Protein localization

Protein localization experiments using *B. subtilis* MS42 (*Pxyl-ytrD-msfgfp*) and PH4 (*Pxyl-ytrE-msfGFP*) were performed with exponentially growing cultures (OD_600_ ~ 0.3). Localization of YtrD was examined in cells grown at constant 37°C or 24°C or in cultures grown overnight at 37°C and shifted to 24°C upon dilution. Antibiotic effects on YtrD localization were examined at 37°C. Expression of the fusion protein was induced with 0.25% xylose. Antibiotic concentrations were selected based on acute shock experiments performed with *B. subtilis* 168CA under the exact conditions used for microscopy (34). Concentrations that led to approximately 50% growth inhibition and were used in protein localization experiments are specified in Table S7. Fluorescence light microscopy was performed on a Nikon Eclipse Ti2 inverted fluorescence microscope equipped with a CFI Plan Apochromat objective (DM Lambda 100× Oil N.A. 1.45, W.D. 0.13 mm, Ph3), a Lumencor Sola SE II FISH 365 light source, a Photometrics PRIME BSI camera, an Okolab incubator, and Nis ELEMENTS AR 5.21.03 software. All microscopy images were processed and analyzed with Fiji (65).

### Acetic acid/methanol fixation

*B. subtilis* overnight cultures were diluted to an OD_600_of ~ 0.05, and all strains were grown until the exponential growth phase (OD_600_ 0.3–0.8). Samples of 200 μL were fixed in 800 μL of a 1:3 mixture of acetic acid and methanol. Fixed samples were spotted (3-4 × 0.5 μL) on glass slides that were coated with a thin film of 1.2% agarose (66), covered with a coverslip, and examined by phase contrast microscopy. Cell wall damage was quantified by counting protruding protoplasts (bubbles on the cell surface) and expressed as the percentage of bubbles per total cell count.

### BODIPY FL vancomycin (Van-FL) staining

Van-FL (V34850, Thermo Scientific) was dissolved in sterile water at a stock concentration of 1 mg/mL. Aliquots were stored at −20°C until further use. On the day of the experiment, the Van-FL stock was mixed 1:1 with unlabeled vancomycin, resulting in a 0.5 mg/mL Van-FL working solution. *B. subtilis* overnight cultures were diluted to an OD_600_ of ~ 0.05, and all strains were grown until exponential growth phase (OD_600_ 0.3–0.8). Samples of 200 µL were transferred to Eppendorf tubes and stained with 0.4 µL of the Van-FL working stock. After 2 min of staining, cells were observed by fluorescence microscopy. Septal Van-FL accumulation was quantified by lateral line scans through the center of the cell (see Fig. S16). Ten cells per strain and condition were semi-randomly selected for line scans, that is, lysed cells, out-of-focus cells, chains, overlapping cells, and cells that were too deformed to draw a straight lateral line were excluded from the analysis.

### BOCILLIN FL penicillin (bocillin) staining

Bocillin (B13233, Thermo Scientific) was dissolved in sterile water at a stock concentration of 1 mg/mL. Aliquots were stored at −20°C until further use. *B. subtilis* overnight cultures were diluted to an OD_600_ of ~ 0.05, and all strains were grown until exponential growth phase (OD_600_ 0.3–0.8). Samples of 200 µL were transferred to Eppendorf tubes and stained with 0.2 µL bocillin. After 10 min of staining, cells were observed by fluorescence microscopy.

### Bacterial growth and lysis experiments

*B. subtilis* overnight cultures were diluted to an OD_600_ of ~ 0.05, and all strains were grown until exponential growth phase (OD_600_ 0.3-0.8). Cultures were then adjusted to an OD_600_ of 0.05 and transferred to sterile 96-well plates. The growth of the cultures was followed by optical density readings in 5 min intervals. Measurements were recorded using a BMG Clariostar Plus plate reader and continued until cultures reached the stationary phase. Cultures were either grown at constant temperature (24°C, 37°C) or subjected to temperature shifts after adjustment to OD_600_ 0.05 (37°C–24°C, and 37°C–16°C). For temperature-dependent lysis experiments, cultures were shifted from 37°C to 4°C. The OD_600_ of cultures shifted to 4°C was measured in 8–16 h intervals over 2 days. For growth on solid medium, 5 µL of exponentially growing cultures were spotted on LB agar plates and streaked out with an inoculation loop. Growth and colony morphology were examined after 16 h of incubation at 37°C.

### Laurdan spectroscopy

Laurdan generalized polarization (GP) was measured as described previously (67, 68). In short, *B. subtilis* strains were grown in LB supplemented with 0.2% glucose. After reaching an OD_600_ of 0.6, cultures were stained with 10 µM laurdan for 5 min, washed four times with prewarmed (37°C or 24°C) laurdan buffer (phosphate-buffered saline (Sigma-Aldrich), 0.2% glucose, 1% dimethylformamide), and resuspended to an OD_600_ of 0.3 using the same buffer. Kinetic laurdan measurements were performed at an excitation wavelength of 350 nm and emission wavelengths of 460 and 500 nm using a BMG Clariostar Plus plate reader. Laurdan GP was calculated using the formula:


$$GP=\frac{I_{435}-I_{500}}{I_{435}+I_{500}}$$

### Sporulation assays

Sporulation assays were performed at 37°C. Sporulation of strains 168CA (WT), PG344 (*spoIIIE::ery*), PH5 (*ytrA::ery*), and GP2646 (*ytrGABCDEF::ery*) was induced by a published resuspension method (69) with some modifications. Overnight cultures were grown in BMM (70), diluted to an OD_500_ of 0.05 in fresh BMM, and grown to an OD_500_ of 0.5. Cultures were then harvested by centrifugation (16,200 × *g*, 2 min, 37°C). Cell pellets were resuspended in 1 mL prewarmed BMM without glucose, and the OD_500_ was readjusted to 0.5. Subsequently, 0.5 µg/mL of the membrane dye FM4-64 was added to the cell suspension, and incubation of the cells was continued. Fluorescence microscopy images were taken every hour from 2 to 10 h, and at 24 h after resuspension. Spore heat resistance assays were performed at 24 h after resuspension. Sporulation efficiency was determined by heating cells to 80°C for 10 min. Serial dilutions of heated and unheated samples were spread on LB agar plates and incubated at 37°C overnight. Spore yield was expressed as the ratio of the number of colonies in heated and unheated samples. Experiments were performed in biological duplicates.

### Statistical analysis

Unless stated otherwise, experiments were performed in biological triplicates, and numerical values represent the average of biologically independent experiments. Error bars represent the standard deviation of the mean. Where appropriate, *P* values were calculated with unpaired, heteroscedastic *t*-tests using OriginPro (OriginLab Corporation, versions 2023).

## SUPPLEMENTAL MATERIAL

Supplemental tables and figures spectrum.02694-25-s0001.pdf Tables S1 to S7 and Figures S1 to S46.

ASM does not own the copyrights to Supplemental Material that may be linked to, or accessed through, an article. The authors have granted ASM a non-exclusive, world-wide license to publish the Supplemental Material files. Please contact the corresponding author directly for reuse.

## References

[1] Adams DW, Errington J. 2009. Bacterial cell division: assembly, maintenance and disassembly of the Z ring. Nat Rev Microbiol 7:642–653. doi:10.1038/nrmicro2198 19680248

[2] Errington J, Wu LJ. 2017. Cell cycle machinery in Bacillus subtilis. Subcell Biochem 84:67–101. doi:10.1007/978-3-319-53047-5_3 28500523 PMC6126333

[3] McKenney PT, Driks A, Eichenberger P. 2013. The Bacillus subtilis endospore: assembly and functions of the multilayered coat. Nat Rev Microbiol 11:33–44. doi:10.1038/nrmicro2921 23202530 PMC9910062

[4] Galinier A, Foulquier E, Pompeo F. 2021. Metabolic control of cell elongation and cell division in Bacillus subtilis. Front Microbiol 12:697930. doi:10.3389/fmicb.2021.697930 34248920 PMC8270655

[5] Su Y, Liu C, Fang H, Zhang D. 2020. Bacillus subtilis: a universal cell factory for industry, agriculture, biomaterials and medicine. Microb Cell Fact 19:173. doi:10.1186/s12934-020-01436-8 32883293 PMC7650271

[6] Akinsemolu AA, Onyeaka H, Odion S, Adebanjo I. 2024. Exploring Bacillus subtilis: ecology, biotechnological applications, and future prospects. J Basic Microbiol 64:e2300614. doi:10.1002/jobm.202300614 38507723

[7] Wenzel M, Bandow JE. 2011. Proteomic signatures in antibiotic research. Proteomics 11:3256–3268. doi:10.1002/pmic.201100046 21726050

[8] Bandow JE, Brötz H, Leichert LIO, Labischinski H, Hecker M, Brotz H, Leichert LIO, Labischinski H, Hecker M. 2003. Proteomic approach to understanding antibiotic action. Antimicrob Agents Chemother 47:948–955. doi:10.1128/AAC.47.3.948-955.2003 12604526 PMC149304

[9] Wenzel M, Patra M, Albrecht D, Chen DYK, Nicolaou KC, Metzler-Nolte N, Bandow JE. 2011. Proteomic signature of fatty acid biosynthesis inhibition available for in vivo mechanism-of-action studies. Antimicrob Agents Chemother 55:2590–2596. doi:10.1128/AAC.00078-11 21383089 PMC3101464

[10] Wenzel M, Kohl B, Münch D, Raatschen N, Albada HB, Hamoen L, Metzler-Nolte N, Sahl HG, Bandow JE. 2012. Proteomic response of Bacillus subtilis to lantibiotics reflects differences in interaction with the cytoplasmic membrane. Antimicrob Agents Chemother 56:5749–5757. doi:10.1128/AAC.01380-12 22926563 PMC3486579

[11] Senges CHR, Stepanek JJ, Wenzel M, Raatschen N, Ay Ü, Märtens Y, Prochnow P, Vázquez Hernández M, Yayci A, Schubert B, et al.. 2020. Comparison of proteomic responses as global approach to antibiotic mechanism of action elucidation. Antimicrob Agents Chemother 65:e01373-20. doi:10.1128/AAC.01373-20 33046497 PMC7927858

[12] Quentin Y, Fichant G, Denizot F. 1999. Inventory, assembly and analysis of Bacillus subtilis ABC transport systems. J Mol Biol 287:467–484. doi:10.1006/jmbi.1999.2624 10092453

[13] Yoshida KI, Fujita Y, Ehrlich SD. 2000. An operon for a putative ATP-binding cassette transport system involved in acetoin utilization of Bacillus subtilis. J Bacteriol 182:5454–5461. doi:10.1128/JB.182.19.5454-5461.2000 10986249 PMC110989

[14] Salzberg LI, Luo Y, Hachmann A-B, Mascher T, Helmann JD. 2011. The Bacillus subtilis GntR family repressor YtrA responds to cell wall antibiotics. J Bacteriol 193:5793–5801. doi:10.1128/JB.05862-11 21856850 PMC3187214

[15] Benda M, Schulz LM, Stülke J, Rismondo J. 2021. Influence of the ABC transporter YtrBCDEF of Bacillus subtilis on competence, biofilm formation and cell wall thickness. Front Microbiol 12:587035. doi:10.3389/fmicb.2021.587035 33897624 PMC8060467

[16] Pedreira T, Elfmann C, Stülke J. 2022. The current state of SubtiWiki, the database for the model organism Bacillus subtilis. Nucleic Acids Res 50:D875–D882. doi:10.1093/nar/gkab943 34664671 PMC8728116

[17] Mahendran A, Orlando BJ. n.d. Genome wide structural prediction of ABC transporter systems in Bacillus subtilis. Front Microbiol 15. doi:10.3389/fmicb.2024.1469915 PMC11466899 39397791

[18] Yu P, Krah BS, Orlando MA, Subramanian S, Orlando BJ. 2025. Structural analysis of a Gram-positive type VII ABC transporter induced by cell wall-targeting antibiotics. Structure. doi:10.1016/j.str.2025.10.004 PMC13042256 41161309

[19] Nicolas P, Mäder U, Dervyn E, Rochat T, Leduc A, Pigeonneau N, Bidnenko E, Marchadier E, Hoebeke M, Aymerich S, et al.. 2012. Condition-dependent transcriptome reveals high-level regulatory architecture in Bacillus subtilis. Science 335:1103–1106. doi:10.1126/science.1206848 22383849

[20] Stepanek JJ, Lukežič T, Teichert I, Petković H, Bandow JE. 2016. Dual mechanism of action of the atypical tetracycline chelocardin. Biochim Biophys Acta 1864:645–654. doi:10.1016/j.bbapap.2016.03.004 26969785

[21] Müller A, Wenzel M, Strahl H, Grein F, Saaki TNV, Kohl B, Siersma T, Bandow JE, Sahl H-G, Schneider T, Hamoen LW. 2016. Daptomycin inhibits cell envelope synthesis by interfering with fluid membrane microdomains. Proc Natl Acad Sci USA 113:E7077–E7086. doi:10.1073/pnas.1611173113 27791134 PMC5111643

[22] Wenzel M, Chiriac AI, Otto A, Zweytick D, May C, Schumacher C, Gust R, Albada HB, Penkova M, Krämer U, Erdmann R, Metzler-Nolte N, Straus SK, Bremer E, Becher D, Brötz-Oesterhelt H, Sahl H-G, Bandow JE. 2014. Small cationic antimicrobial peptides delocalize peripheral membrane proteins. Proc Natl Acad Sci USA 111:E1409–E1418. doi:10.1073/pnas.1319900111 24706874 PMC3986158

[23] Münch D, Müller A, Schneider T, Kohl B, Wenzel M, Bandow JE, Maffioli S, Sosio M, Donadio S, Wimmer R, Sahl H-G. 2014. The lantibiotic NAI-107 binds to bactoprenol-bound cell wall precursors and impairs membrane functions. J Biol Chem 289:12063–12076. doi:10.1074/jbc.M113.537449 24627484 PMC4002112

[24] Cao M, Wang T, Ye R, Helmann JD. 2002. Antibiotics that inhibit cell wall biosynthesis induce expression of the Bacillus subtilis σ^W^ and σ^M^ regulons. Mol Microbiol 45:1267–1276. doi:10.1046/j.1365-2958.2002.03050.x 12207695

[25] Mascher T, Margulis NG, Wang T, Ye RW, Helmann JD. 2003. Cell wall stress responses in Bacillus subtilis: the regulatory network of the bacitracin stimulon. Mol Microbiol 50:1591–1604. doi:10.1046/j.1365-2958.2003.03786.x 14651641

[26] Schneider T, Kruse T, Wimmer R, Wiedemann I, Sass V, Pag U, Jansen A, Nielsen AK, Mygind PH, Raventós DS, Neve S, Ravn B, Bonvin A, De Maria L, Andersen AS, Gammelgaard LK, Sahl H-G, Kristensen H-H. 2010. Plectasin, a fungal defensin, targets the bacterial cell wall precursor Lipid II. Science 328:1168–1172. doi:10.1126/science.1185723 20508130

[27] Hutter B, Fischer C, Jacobi A, Schaab C, Loferer H. 2004. Panel of Bacillus subtilis reporter strains indicative of various modes of action. Antimicrob Agents Chemother 48:2588–2594. doi:10.1128/AAC.48.7.2588-2594.2004 15215113 PMC434206

[28] Zhang Q, Cornilleau C, Müller RR, Meier D, Flores P, Guérin C, Wolf D, Fromion V, Carballido-Lopez R, Mascher T. 2023. Comprehensive and comparative transcriptional profiling of the cell wall stress response in Bacillus subtilis. bioRxiv. doi:10.1101/2023.02.03.526509 41992703

[29] Koo B-M, Kritikos G, Farelli JD, Todor H, Tong K, Kimsey H, Wapinski I, Galardini M, Cabal A, Peters JM, Hachmann A-B, Rudner DZ, Allen KN, Typas A, Gross CA. 2017. Construction and analysis of two genome-scale deletion libraries for Bacillus subtilis. Cell Syst 4:291–305. doi:10.1016/j.cels.2016.12.013 28189581 PMC5400513

[30] Zhu X, Liu D, Singh AK, Drolia R, Bai X, Tenguria S, Bhunia AK. 2018. Tunicamycin mediated inhibition of wall teichoic acid affects Staphylococcus aureus and Listeria monocytogenes cell morphology, biofilm formation and virulence. Front Microbiol 9:1352. doi:10.3389/fmicb.2018.01352 30034372 PMC6043806

[31] Bucher T, Oppenheimer-Shaanan Y, Savidor A, Bloom-Ackermann Z, Kolodkin-Gal I. 2015. Disturbance of the bacterial cell wall specifically interferes with biofilm formation. Environ Microbiol Rep 7:990–1004. doi:10.1111/1758-2229.12346 26472159

[32] Schäfer A-B, Sidarta M, Abdelmesseh Nekhala I, Marinho Righetto G, Arshad A, Wenzel M. 2024. Dissecting antibiotic effects on the cell envelope using bacterial cytological profiling: a phenotypic analysis starter kit. Microbiol Spectr 12:e0327523. doi:10.1128/spectrum.03275-23 38289933 PMC10913488

[33] Schäfer A-B, Wenzel M. 2020. A how-to guide for mode of action analysis of antimicrobial peptides. Front Cell Infect Microbiol 10:540898. doi:10.3389/fcimb.2020.540898 33194788 PMC7604286

[34] Strahl H, Bürmann F, Hamoen LW. 2014. The actin homologue MreB organizes the bacterial cell membrane. Nat Commun 5:3442. doi:10.1038/ncomms4442 24603761 PMC3955808

[35] Strahl H, Hamoen LW. 2010. Membrane potential is important for bacterial cell division. Proc Natl Acad Sci USA 107:12281–12286. doi:10.1073/pnas.1005485107 20566861 PMC2901462

[36] Schneider T, Sahl HG. 2010. An oldie but a goodie - cell wall biosynthesis as antibiotic target pathway. Int J Med Microbiol 300:161–169. doi:10.1016/j.ijmm.2009.10.005 20005776

[37] Wenzel M, Rautenbach M, Vosloo JA, Siersma T, Aisenbrey CHM, Zaitseva E, Laubscher WE, van Rensburg W, Behrends JC, Bechinger B, Hamoen LW. 2018. The multifaceted antibacterial mechanisms of the pioneering peptide antibiotics tyrocidine and gramicidin S. mBio 9:e00802-18. doi:10.1128/mBio.00802-18 30301848 PMC6178620

[38] Domínguez-Escobar J, Wolf D, Fritz G, Höfler C, Wedlich-Söldner R, Mascher T. 2014. Subcellular localization, interactions and dynamics of the phage-shock protein-like Lia response in Bacillus subtilis. Mol Microbiol 92:716–732. doi:10.1111/mmi.12586 24666271

[39] Beckering CL, Steil L, Weber MHW, Völker U, Marahiel MA. 2002. Genomewide transcriptional analysis of the cold shock response in Bacillus subtilis. J Bacteriol 184:6395–6402. doi:10.1128/JB.184.22.6395-6402.2002 12399512 PMC151959

[40] Sidarta M, Lorente Martín AI, Monsalve A, Marinho Righetto G, Schäfer A-B, Wenzel M. 2024. Lipid phase separation impairs membrane thickness sensing by the Bacillus subtilis sensor kinase DesK. Microbiol Spectr 12:e0392523. doi:10.1128/spectrum.03925-23 38717171 PMC11237406

[41] Schirner K, Eun Y-J, Dion M, Luo Y, Helmann JD, Garner EC, Walker S. 2015. Lipid-linked cell wall precursors regulate membrane association of bacterial actin MreB. Nat Chem Biol 11:38–45. doi:10.1038/nchembio.1689 25402772 PMC4270829

[42] Kamal El-Sagheir AM, Abdelmesseh Nekhala I, Abd El-Gaber MK, Aboraia AS, Persson J, Schäfer A-B, Wenzel M, Omar FA. 2023. N4-substituted piperazinyl norfloxacin derivatives with broad-spectrum activity and multiple mechanisms on gyrase, topoisomerase IV, and bacterial cell wall synthesis. ACS Bio Med Chem Au 3:494–506. doi:10.1021/acsbiomedchemau.3c00038 PMC10739246 38144255

[43] Young FE. 1966. Autolytic enzyme associated with cell walls of Bacillus subtilis. J Biol Chem 241:3462–3467. doi:10.1016/S0021-9258(18)99855-8 4958491

[44] Blackman SA, Smith TJ, Foster SJ. 1998. The role of autolysins during vegetative growth of Bacillus subtilis 168. Microbiology (Reading) 144:73–82. doi:10.1099/00221287-144-1-73 9537764

[45] Vollmer W, Joris B, Charlier P, Foster SBP. 2008. Bacterial peptidoglycan (murein) hydrolases. FEMS Microbiol Rev 32:259–286. doi:10.1111/j.1574-6976.2007.00099.x 18266855

[46] Tasaki S, Nakayama M, Shoji W. 2017. Morphologies of Bacillus subtilis communities responding to environmental variation. Dev Growth Differ 59:369–378. doi:10.1111/dgd.12383 28675458

[47] Baudu E, Raspaud E, Fontagné-Faucher C, Nait Chabane Y, Marcato-Romain C-E. 2025. Morphogenesis and mechanical properties of Bacillus amyloliquefaciens biofilms: a comparative study of rough and smooth morphotypes. Curr Res Microb Sci 8:100403. doi:10.1016/j.crmicr.2025.100403 40487030 PMC12141842

[48] Hamouche L, Laalami S, Daerr A, Song S, Holland IB, Séror SJ, Hamze K, Putzer H. 2017. Bacillus subtilis swarmer cells lead the swarm, multiply, and generate a trail of quiescent descendants. mBio 8:e02102-16. doi:10.1128/mBio.02102-16 28174308 PMC5296600

[49] Kim H, Singh AK, Bhunia AK, Bae E. 2014. Laser-induced speckle scatter patterns in Bacillus colonies. Front Microbiol 5:537. doi:10.3389/fmicb.2014.00537 25352840 PMC4196546

[50] Gray DA, Dugar G, Gamba P, Strahl H, Jonker MJ, Hamoen LW. 2019. Extreme slow growth as alternative strategy to survive deep starvation in bacteria. Nat Commun 10:890. doi:10.1038/s41467-019-08719-8 30792386 PMC6385201

[51] Sidarta M, Li D, Hederstedt L, Bukowska-Faniband E. 2018. Forespore targeting of SpoVD in Bacillus subtilis is mediated by the N-terminal part of the protein. J Bacteriol 200:200. doi:10.1128/JB.00163-18 PMC5996694 29661861

[52] Khanna K, Lopez-Garrido J, Sugie J, Pogliano K, Villa E. 2021. Asymmetric localization of the cell division machinery during Bacillus subtilis sporulation. eLife 10:e62204. doi:10.7554/eLife.62204 34018921 PMC8192124

[53] Haydon DJ, Guest JR. 1991. A new family of bacterial regulatory proteins. FEMS Microbiol Lett 63:291–295. doi:10.1016/0378-1097(91)90101-f 2060763

[54] Suvorova IA, Korostelev YD, Gelfand MS. 2015. GntR family of bacterial transcription factors and their DNA binding motifs: structure, positioning and co-evolution. PLoS One 10:e0132618. doi:10.1371/journal.pone.0132618 26151451 PMC4494728

[55] Svarachorn A, Tsuchido T, Shinmyo A, Takano M. 1991. Autolysis of Bacillus subtilis induced by low temperature. J Ferment Bioeng 71:281–283. doi:10.1016/0922-338X(91)90283-M

[56] Cooney PH, Whiteman PF, Freese E. 1977. Media dependence of commitment in Bacillus subtilis. J Bacteriol 129:901–907. doi:10.1128/jb.129.2.901-907.1977 402360 PMC235028

[57] Verma N, Singh NA, Kumar N, Raghu HV. 2013. Screening of different media for sporulation of Bacillus megaterium. Int J Microbiol Res Rev 1:68–073.

[58] Mutlu A, Kaspar C, Becker N, Bischofs IB. 2020. A spore quality–quantity tradeoff favors diverse sporulation strategies in Bacillus subtilis. ISME J 14:2703–2714. doi:10.1038/s41396-020-0721-4 32724142 PMC7784978

[59] Spizizen J. 1958. Transformation of biochemically deficient strains of Bacillus subtilis by deoxyribonucleate. Proc Natl Acad Sci USA 44:1072–1078. doi:10.1073/pnas.44.10.1072 16590310 PMC528696

[60] Gibson DG, Young L, Chuang R-Y, Venter JC, Hutchison CA, Smith HO. 2009. Enzymatic assembly of DNA molecules up to several hundred kilobases. Nat Methods 6:343–345. doi:10.1038/nmeth.1318 19363495

[61] Green MR, Sambrook J. 2017. Isolation of high-molecular-weight DNA using organic solvents. Cold Spring Harb Protoc 2017:356–359. doi:10.1101/pdb.prot093450 28373491

[62] Hanahan D, Jessee J, Bloom FR. 1991. Plasmid transformation of Escherichia coli and other bacteria. Methods Enzymol 204:63–113. doi:10.1016/0076-6879(91)04006-a 1943786

[63] Saeloh D, Tipmanee V, Jim KK, Dekker MP, Bitter W, Voravuthikunchai SP, Wenzel M, Hamoen LW. 2018. The novel antibiotic rhodomyrtone traps membrane proteins in vesicles with increased fluidity. PLoS Pathog 14:e1006876. doi:10.1371/journal.ppat.1006876 29451901 PMC5833292

[64] CLSI. 2013. 23rd Informational Supplement M100-S23. In Performance standards for antimicrobial susceptibility testing. CLSI, Wayne, PA, USA.

[65] Schindelin J, Arganda-Carreras I, Frise E, Kaynig V, Longair M, Pietzsch T, Preibisch S, Rueden C, Saalfeld S, Schmid B, Tinevez JY, White DJ, Hartenstein V, Eliceiri K, Tomancak P, Cardona A. 2012. Fiji: an open-source platform for biological-image analysis. Nat Methods 9:676–682. doi:10.1038/nmeth.2019 22743772 PMC3855844

[66] Te Winkel JD, Gray DA, Seistrup KH, Hamoen LW, Strahl H. 2016. Analysis of antimicrobial-triggered membrane depolarization using voltage sensitive dyes. Front Cell Dev Biol 4:29. doi:10.3389/fcell.2016.00029 27148531 PMC4829611

[67] Wenzel M, Vischer NOE, Strahl H, Hamoen LW. 2018. Assessing membrane fluidity and visualizing fluid membrane domains in bacteria using fluorescent membrane dyes. Bio Protoc 8:e3063. doi:10.21769/BioProtoc.3063 PMC8342135 34532528

[68] Schäfer A-B, Steenhuis M, Jim KK, Neef J, O’Keefe S, Whitehead RC, Swanton E, Wang B, Halbedel S, High S, van Dijl JM, Luirink J, Wenzel M. 2023. Dual action of eeyarestatin 24 on sec-dependent protein secretion and bacterial DNA. ACS Infect Dis 9:253–269. doi:10.1021/acsinfecdis.2c00404 36637435 PMC9926488

[69] Nicholson WL, Setlow PS. 1991. Sporulation, germination and outgrowth, p 391–450. In Harwood CR, Cutting SM (ed), Molecular biological methods for Bacillus. John Wiley & Sons Ltd, Chichester.

[70] Stülke J, Hanschke R, Hecker M. 1993. Temporal activation of β-glucanase synthesis in Bacillus subtilis is mediated by the GTP pool. J Gen Microbiol 139:2041–2045. doi:10.1099/00221287-139-9-2041 8245830

