# Supplementary material for: Contribution of the Bacillus subtilis ytrGABCDEF operon to antibiotic stress adaptation
Source: Microbiol Spectr. 2025 Dec 5;14(1):e02694-25. doi: 10.1128/spectrum.02694-25 (PMC12772383; doi:10.1128/spectrum.02694-25)
Supplement: Supplemental tables and figures — Tables S1 to S7 and Figures S1 to S46. [file spectrum.02694-25-s0001.pdf]

Supplementary Information

Contribution of the *Bacillus subtilis ytrGABCDEF* operon to antibiotic stress adaptation

Luna Baruah[1,2&], Margareth Sidarta[1,2&], Pauline Hammer úr Skúoy[1], Olivia Johnsson[1], Emma Frisk[1], Paula Didelot[1], Aysha Arshad[1], Michaela Wenzel[1,2]*

[1]Division of Chemical Biology, Department of Life Sciences, Chalmers University of Technology, 412 96 Gothenburg, Sweden
[2]Centre for Antibiotic Resistance Research in Gothenburg (CARe), 413 46 Gothenburg, Sweden

[&]These authors contributed equally to this work.

*Corresponding author: Michaela Wenzel (wenzelm@chalmers.se)

Supplementary tables

Table S1: Upregulation of YtrB and YtrE protein levels after treatment with cell wall biosynthesis-targeting antibiotics
Table S2: Induction of *ytr* transcript levels after treatment with antibiotics
Table S3: MICs of different antibiotics against *ytr* deletion mutants
Table S4: MICs of different β-lactam antibiotics against *ytr* deletion mutants
Table S5: Strains and plasmids used in this work
Table S6: Primers used in this work
Table S7: Antibiotics and concentrations used for protein localization experiments

Supplementary figures

Figure S1: Transcript levels of *ytrA, ytrB, ytrC, ytrD, ytrE,* and *ytrF* under diverse culture conditions
Figure S2: MICs of different antibiotics against *ytr* deletion mutants at 37 °C
Figure S3: MICs of different antibiotics against *ytr* deletion mutants at 24 °C
Figure S4: Growth curves of *B. subtilis* 168CA and *ytr* mutants at 37 °C **(A)** and 24 °C **(B)** depicted on a log scale.
Figure S5: Growth curves of 168CA (WT) and *ytr* deletion mutants after acute shock with different concentrations of ampicillin at 37 °C
Figure S6: Growth curves of 168CA (WT) and *ytr* deletion mutants after acute shock with different concentrations of ampicillin at 24 °C
Figure S7: Growth curves of 168CA (WT) and *ytr* deletion mutants after acute shock with different concentrations of nisin at 37 °C
Figure S8: Growth curves of 168CA (WT) and *ytr* deletion mutants after acute shock with different concentrations of nisin at 24 °C
Figure S9: Growth curves of 168CA (WT) and *ytr* deletion mutants after acute shock with different concentrations of ertapenem at 37 °C
Figure S10: Growth curves of 168CA (WT) and *ytr* deletion mutants after acute shock with different concentrations of ertapenem at 24 °C
Figure S11: Localization of YtrD-GFP at different temperatures **(A)** and YtrE-GFP **(B)**
Figure S12: Localization of YtrD under antibiotic stress
Figure S13: Laurdan spectroscopy of deletion mutants at 37 °C and 24 °C
Figure S14: Microscopy images of *B. subtilis* 168CA (WT) and *ytr* mutants labeled with Van-FL
Figure S15: Quantification of whole cell fluorescence from microscopy images of *B. subtilis* strains labeled with Van-FL
Figure S16: Longitudinal line scans of individual *B. subtilis* 168CA (WT) cells grown at 37 °C and stained with Van-FL in exponential growth phase
Figure S17: Longitudinal line scans of individual *B. subtilis* PH5 (Δ*ytrA*) cells grown at 37 °C and stained with Van-FL in exponential growth phase
Figure S18: Longitudinal line scans of individual *B. subtilis* PH1 (Δ*ytrB*) cells grown at 37 °C and stained with Van-FL in exponential growth phase
Figure S19: Longitudinal line scans of individual *B. subtilis* PD3 (Δ*ytrC*) cells grown at 37 °C and stained with Van-FL in exponential growth phase
Figure S20: Longitudinal line scans of individual *B. subtilis* PD2 (Δ*ytrD*) cells grown at 37 °C and stained with Van-FL in exponential growth phase

Figure S21: Longitudinal line scans of individual *B. subtilis* PH2 (Δ*ytrE*) cells grown at 37 °C and stained with Van-FL in exponential growth phase
Figure S22: Longitudinal line scans of individual *B. subtilis* PD1 (Δ*ytrF*) cells grown at 37 °C and stained with Van-FL in exponential growth phase
Figure S23: Longitudinal line scans of individual *B. subtilis* GP3193 (Δ*ytrAB*) cells grown at 37 °C and stained with Van-FL in exponential growth phase
Figure S24: Longitudinal line scans of individual *B. subtilis* GP3196 (Δ*ytrAE*) cells grown at 37 °C and stained with Van-FL in exponential growth phase
Figure S25: Longitudinal line scans of individual *B. subtilis* GP3206 (Δ*ytrABE*) cells grown at 37 °C and stained with Van-FL in exponential growth phase
Figure S26: Longitudinal line scans of individual *B. subtilis* BLMS3 (Δ*ytrACD*) cells grown at 37 °C and stained with Van-FL in exponential growth phase
Figure S27: Longitudinal line scans of individual *B. subtilis* GP2646 (Δ*ytrGABCDEF*) cells grown at 37 °C and stained with Van-FL in exponential growth phase
Figure S28: Longitudinal line scans of individual *B. subtilis* 168CA (WT) cells grown at 24 °C and stained with Van-FL in exponential growth phase
Figure S29: Longitudinal line scans of individual *B. subtilis* PH5 (Δ*ytrA*) cells grown at 24 °C and stained with Van-FL in exponential growth phase
Figure S30: Longitudinal line scans of individual *B. subtilis* PH1 (Δ*ytrB*) cells grown at 24 °C and stained with Van-FL in exponential growth phase
Figure S31: Longitudinal line scans of individual *B. subtilis* PD3 (Δ*ytrC*) cells grown at 24 °C and stained with Van-FL in exponential growth phase
Figure S32: Longitudinal line scans of individual *B. subtilis* PD2 (Δ*ytrD*) cells grown at 24 °C and stained with Van-FL in exponential growth phase
Figure S33: Longitudinal line scans of individual *B. subtilis* PH2 (Δ*ytrE*) cells grown at 24 °C and stained with Van-FL in exponential growth phase
Figure S34: Longitudinal line scans of individual *B. subtilis* PD1 (Δ*ytrF*) cells grown at 24 °C and stained with Van-FL in exponential growth phase
Figure S35: Longitudinal line scans of individual *B. subtilis* GP3193 (Δ*ytrAB*) cells grown at 24 °C and stained with Van-FL in exponential growth phase
Figure S36: Longitudinal line scans of individual *B. subtilis* GP3196 (Δ*ytrAE*) cells grown at 24 °C and stained with Van-FL in exponential growth phase
Figure S37: Longitudinal line scans of individual *B. subtilis* GP3206 (Δ*ytrABE*) cells grown at 24 °C and stained with Van-FL in exponential growth phase
Figure S38: Longitudinal line scans of individual *B. subtilis* BLMS3 (Δ*ytrACD*) cells grown at 24 °C and stained with Van-FL in exponential growth phase
Figure S39: Longitudinal line scans of individual *B. subtilis* GP2646 (Δ*ytrGABCDEF*) cells grown at 24 °C and stained with Van-FL in exponential growth phase
Figure S40: Microscopy images of *B. subtilis* 168CA (WT) and *ytr* mutants grown at 37 °C and labeled with bocillin.
Figure S41: Microscopy images of *B. subtilis* 168CA (WT) and *ytr* mutants grown at 24 °C and labeled with bocillin.
Figure S42: Bubble assay of deletion mutants at 37 °C and 24 °C
Figure S43: Growth curves of *B. subtilis* 168CA and *ytr* mutants after temperature shifts depicted on log scales

Figure S44: Fluorescence and phase contrast microscopy image of sporulating *B. subtilis* PH5 (*ΔytrA*)
Figure S45: Fluorescence and phase contrast microscopy image of *B. subtilis* PG344 (*ΔspoIIE*)
Figure S46: Sporulation efficiency of *B. subtilis* 168CA (wild type), PG344 (*ΔspoIIE*), PH5 (*ΔytrA*), and GP2646 (*ΔytrGABCDEF*)

Table S1: Upregulation of YtrB and YtrE protein levels after treatment with cell wall biosynthesis-targeting antibiotics. Proteomic profiling by 2D PAGE was performed with *B. subtilis* 168CA grown at 37 °C in Belitzky minimal medium according to Wenzel et al.[1]. Note that 2D PAGE experiments were limited to soluble proteins with a pI between 4 and 7, making YtrB and YtrE the only members of the *ytr* operon that can be detected and quantified by this approach. Data are taken from the references indicated in the table.

antibiotic	mode of action	induction factor YtrB	induction factor YtrE	reference
bacitracin	binds C55-PP	20.0	-	2
chelocardin	protein synthesis, membrane*	6.7	-	3
cephalexin	penicillin-binding proteins	-	-	4
daptomycin	binds lipid II and PG, disturbs fluid membrane microdomains	-	-	5
D-cycloserine	lipid II synthesis through inhibition of alanine racemase and D-ala-D-ala ligase	-	-	2
gallidermin	binds lipid II	14.0	21.0	6
mersacidin	binds lipid II	9.8	14.0	6
methicillin	penicillin-binding proteins	-	-	2
MP196	cell membrane, secondary effects on lipid II synthesis by delocalization of MurG	-	>2	7
NAI-107	binds lipid II	9.5	7.8	8
nisin	binds lipid II and C55-PP, forms pores	-	2.1	6
vancomycin	binds lipid II	20.0	20.0	2

Minus (-) indicates no significant upregulation (≥2 fold change in at least three biological replicates was defined as significantly upregulated), PG: phosphatidylglycerol, *mode of action shifts from protein synthesis inhibition to membrane damage with increasing concentration.

Table S2: Induction of *ytr* transcript levels after treatment with antibiotics. All experiments were performed with *B. subtilis* 168CA grown at 37 °C in Luria Bertani medium[9]. Significant induction (log10 ratio ≥1) is indicated with '+', no regulation with '-'. Data are taken from Salzberg *et al.*[9].

		induction (log10 ratio ≥1)					
antibiotic	mode of action	*ytrA*	*ytrB*	*ytrC*	*ytrD*	*ytrE*	*ytrF*
bacitracin	inhibits lipid II synthesis by binding C55-PP	+	+	+	+	+	+
D-cycloserine	inhibits lipid II synthesis through inhibition of alanine racemase and D-ala-D-ala ligase	-	-	-	-	-	-
daptomycin	binds lipid II and PG, disturbs fluid membrane microdomains	-	-	-	-	-	-
fosfomycin	impairs lipid I synthesis by inhibiting MraY	-	-	-	-	-	-
moenomycin	inhibits penicillin-binding proteins	-	-	-	-	-	-
oxacillin	inhibits penicillin-binding proteins	-	-	-	-	-	-
ramoplanin	inhibits cell wall biosynthesis by binding lipid I and lipid II	+	+	+	+	+	+
ristocetin	binds lipid II in a manner similar to vancomycin	+	+	+	+	+	+
vancomycin	binds lipid II	+	+	+	+	+	+

Table S3: MICs of different antibiotics against *ytr* deletion mutants. Abbreviations: ampicillin (amp), nisin (nis), vancomycin (van), D-cycloserine (D-cyc), tetracycline (tet).

	37 ºC				
strains	amp (μg/mL)	nis (μg/mL)	van (μg/mL)	D-cyc (μg/mL)	tet (μg/mL)
168CA	2	6.4	1	128	10.67 ± 4.62
Δ*ytrA*	4	6.4	1	128	16
Δ*ytrB*	2.67 ± 1.15	12.8	1	128	13.33 ± 4.62
Δ*ytrC*	1.67 ± 0.58	6.4	1	256	13.33 ± 4.62
Δ*ytrD*	2.33 ± 1.53	6.4	1	256	13.33 ± 4.62
Δ*ytrE*	3.33 ± 1.15	6.4	1	256	13.33 ± 4.62
Δ*ytrF*	3.33 ± 1.15	6.4	1	128	13.33 ± 4.62
Δ*ytrAB*	0.67 ± 0.29	6.4	1	128	13.33 ± 4.62
Δ*ytrAE*	0.67 ± 0.29	6.4	1	128	13.33 ± 4.62
Δ*ytrABE*	0.83 ± 0.29	6.4	1	128	13.33 ± 4.62
Δ*ytrACD*	1	12.8	1	128	16
Δ*ytrGABCDEF*	1.33 ± 0.58	12.8	1	128	13.33 ± 4.62
	24 ºC				
strains	amp (μg/mL)	nis (μg/mL)	van (μg/mL)	D-cyc (μg/mL)	tet (μg/mL)
168CA	2	3.2	0.5	128	4
Δ*ytrA*	2	0.8	0.5	128	4
Δ*ytrB*	2	3.2	0.5	128	4
Δ*ytrC*	2	12.8	0.5	128	4
Δ*ytrD*	2	6.4	0.5	128	4
Δ*ytrE*	2	1.6	0.34 ± 0.19	128	4
Δ*ytrF*	2	3.2	0.5	128	4
Δ*ytrAB*	1	9.6 ± 3.7	0.75 ± 0.29	128	4
Δ*ytrAE*	1	3.2	0.5	128	4
Δ*ytrABE*	1	3.2	0.5	64	4
Δ*ytrACD*	2	12.8	0.5	64	3.33 ± 0.94
Δ*ytrGABCDEF*	2	3.2	0.5	128	4

Table S4: MICs of different β-lactam antibiotics against *ytr* deletion mutants. Abbreviations: ertapenem (etp), cefoxitin Na (cefo), meropenem (mem), cloxacillin (clx).

strain	MIC							
	37 ºC				24 ºC			
	etp	cefo	mem	clx	etp	cefo	mem	clx
168CA	0.25	1	4	0.1	0.25	1	4	0.5
Δ*ytrA*	0.5	1	4	0.13	0.25	1	4	0.5
Δ*ytrB*	0.13	1	4	0.25	0.25	1	4	0.5
Δ*ytrE*	0.19 ± 0.07	1	4	0.13	0.25	1	4	0.5
Δ*ytrAB*	0.13	0.67 ± 0.29	4	0.05 ± 0.02	0.25	0.67 ± 0.29	4	0.25
Δ*ytrAE*	0.13	0.5	2	0.13	0.13	0.42 ± 0.14	2	0.13
Δ*ytrABE*	0.13	0.5	2	0.06	0.13	0.25	2	0.13
Δ*ytrACD*	0.19 ± 0.09	0.5	4	0.13	0.25	1	4	0.5
Δ*ytrGABCDEF*	0.13	0.67 ± 0.29	2	0.09 ± 0.04	0.25	1	4	0.5

Table S5: Strains and plasmids used in this work. An arrow indicates transformation of the indicated strain with plasmid or chromosomal DNA (DNA→ strain).

strain	genotype	origin
E. coli		
TOP10	*F–mcrA Δ(mrr-hsdRMS-mcrBC) φ80lacZΔM15 ΔlacX74 recA1 araD139 Δ(ara-leu)7697 galU galK λ–rpsL(StrR) endA1 nupG*	ThermoFisher
W3110	*F- λ- thyA36 deoC2 IN1*	[10]
B. subtilis		
168CA	*trpC2*	[11]
BKE30420	*trpC2 ΔytrE::ery*	[12]
BKE30450	*trpC2 ΔytrB::ery*	[12]
BKE30460	*trpC2 ΔytrA::ery*	[12]
BKK30410	*trpC2 ΔytrF::kan*	[12]
BKK30430	*trpC2 ΔytrD::kan*	[12]
BKK30440	*trpC2 ΔytrC::kan*	[12]
BLMS3	*trpC2 ΔytrA::ery ΔytrCD*	[13]
GP2646	*trpC2 ΔytrGABCDEF::ery*	[13]
GP3193	*trpC2 ΔytrA::ery ΔytrB*	[13]
GP3196	*trpC2 ΔytrA::ery ΔytrE*	[13]
GP3206	*trpC2 ΔytrA::ery ΔytrB ΔytrE*	[13]
KS19	*trpC2 ΔlytABC::kan ΔlytD::tet ΔlytE::cat ΔlytF::spc*	[14]
MS42	*trpC2 amyE::spc Pxyl-ytrD-msfgfp*	this work pMS33→168CA
PD1	*trpC2 ΔytrF::kan*	this work BKK30410→168CA
PD2	*trpC2 ΔytrD::kan*	this work BKK30430→168CA
PD3	*trpC2 ΔytrC::kan*	this work BKK30440→168CA
PG344	*spoIIE::ery*	[15]
PH1	*trpC2 ΔytrB::ery*	this work BKE30450→168CA
PH2	*trpC2 ΔytrE::ery*	this work BKE30420→168CA
PH4	*trpC2 amyE::spc Pxyl-ytrE-msfGFP*	this work pPH2→168CA
PH5	*trpC2 ΔytrA::ery*	this work BKE30450→168CA
**plasmids**		
pMS33	*bla amyE::spc Pxyl-ytrD-msfgfp*	this work
pPH2	*bla amyE::spc Pxyl-ytrE-msfgfp*	this work
pMW1	*bla amyE::spc Pxyl-msfgfp*	[5]

Table S6: Primers used in this work.

DNA template	primer	primer sequence	PCR product
Gibson assembly: vector backbone linearization			
pMW1	MWP1	ATGAGCAAAGGAGAAGAACTTTTC	linear pMW1
	Abs1	CCTAGGAATCTCCTTTCTAGATGC	
Gibson assembly: insert amplification			
168CA	MSP152	CTAGAAAGGAGATTCCTAGGTTGCCGGATTCCGGACTG	*ytrD* insert
	MSP153	AGTTCTTCTCCTTTGCTCATGCTTCCGCTTCCGCTTCCGCTTCCGCTTCCGCTTCCCAAAAGTTTCATTTTTTTATAAATCGCAAAATAAC	*ytrD* insert
	PHP14	CTAGAAAGGAGATTCCTAGGATGATTGATGTTCAGCATATCGACC	ytrE insert
	PHP15	AGTTCTTCTCCTTTGCTCATGCCGGAGCCGGAGCCGGAGCCGGAGCCGGAGCCGGAAACCTCAACGGTAATTCCTCC	*ytrE* insert
Plasmid and strain confirmation			
pMS33	TerS21	GGGCAACAAACTAATGTGCAA	1163 bp
	Abs5	GAAAATTTGTGCCCATTAACATCACCATC	
PD1	KanR774	AGTAAGTGGCTTTATTGATCTTGGG	636 bp
	PHP9	ATGTTTTTGGCATCTTCCATGGAGGATAAAAAGAGAATCGGAAC	
PD2, PD3	KanR774	AGTAAGTGGCTTTATTGATCTTGGG	1432 bp (PD2); 2439 bp (PD3)
	PHP20	CGAAGTCGTATCCTACTACGATTTC	
PH1	ErmR815	CCTTAAAACATGCAGGAATTGACG	634 bp
	PHP19	GCTGCTGTGCTCAAAAAAGTAG	
PH2	ErmR815	CCTTAAAACATGCAGGAATTGACG	637 bp
	PHP20	CGAAGTCGTATCCTACTACGATTTC	
PH5	MSP101	GCTCATTGGCATTACTTTTAATGGC	637 bp
	PHP19	GCTGCTGTGCTCAAAAAAGTAG	
MS42, PH4	Absp12	CTGATTTATGAACAAAAAAGAAACCATCATTG	3578 bp (MS42); 3299 bp (PH4)
	Abs5	GAAAATTTGTGCCCATTAACATCACCATC	

Table S7: Antibiotics and concentrations used for protein localization experiments.

antibiotic	target/mechanism	concentration
ampicillin	cell wall, penicillin-binding proteins (PBPs), transpeptidase[16]	2 µg/mL
nisin	cell wall, lipid II[17], membrane[18], pore-former[19]	6.4 µg/mL
vancomycin	cell wall, lipid II[5,20,21]	0.8 µg/mL
D-cycloserine	cell wall, alanine racemase and D-ala:D-ala ligase[22]	32 µg/mL
erythromycin	protein synthesis, 50S ribosomal subunit[23]	1 µg/mL
tetracycline	protein synthesis, 30S ribosomal subunit[24,25]	10.7 µg/mL
CCCP	proton ionophore[26,27]	100 µM

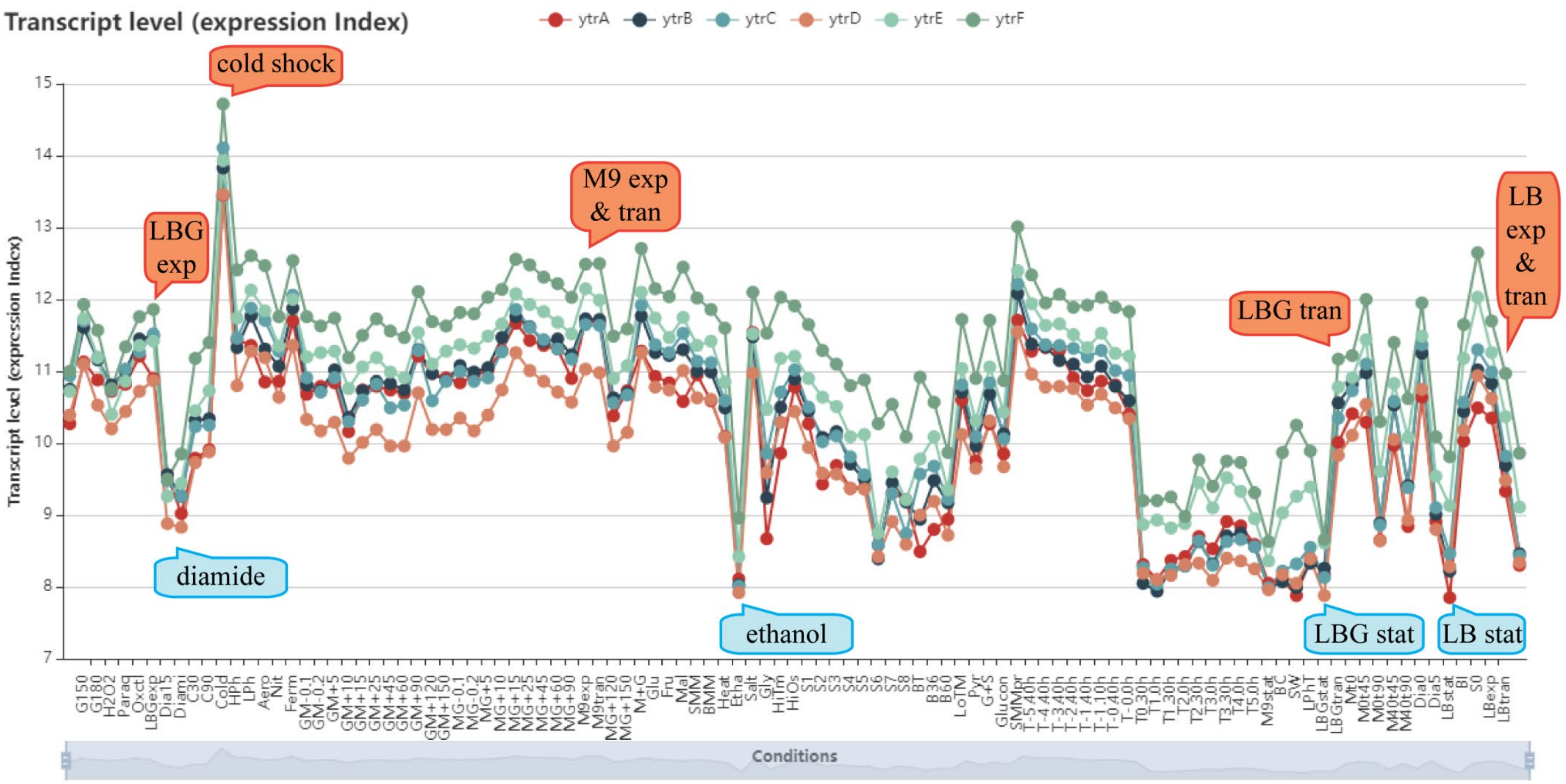

Figure S1: Transcript levels of *ytrA, ytrB, ytrC, ytrD, ytrE,* and *ytrF* under diverse culture conditions[28]. The expression index graph was obtained from the SubtiWiki expression browser[29]. Boxes indicate conditions of interest with high (orange) and low (blue) expression levels. Abbreviations: LB supplemented with 0.3% glucose (LBG), exponential/log phase (exp), transition phase (tran), stationary phase (stat). A full list of conditions pertaining to other conditions can be found in the SubtiWiki expression browser.

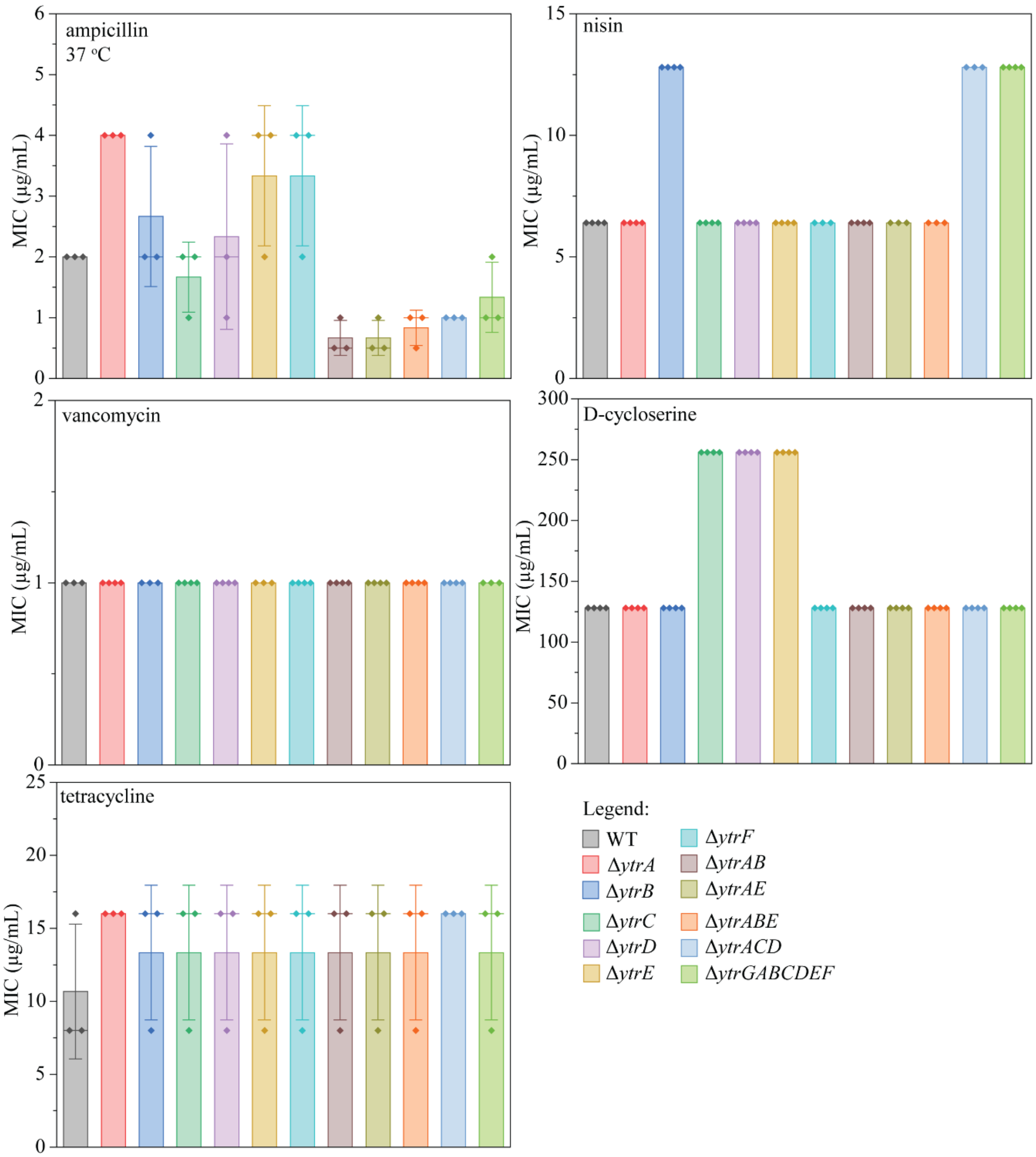

Figure S2: MICs of different antibiotics against *ytr* deletion mutants at 37 °C. Error bars show standard deviation of the mean.

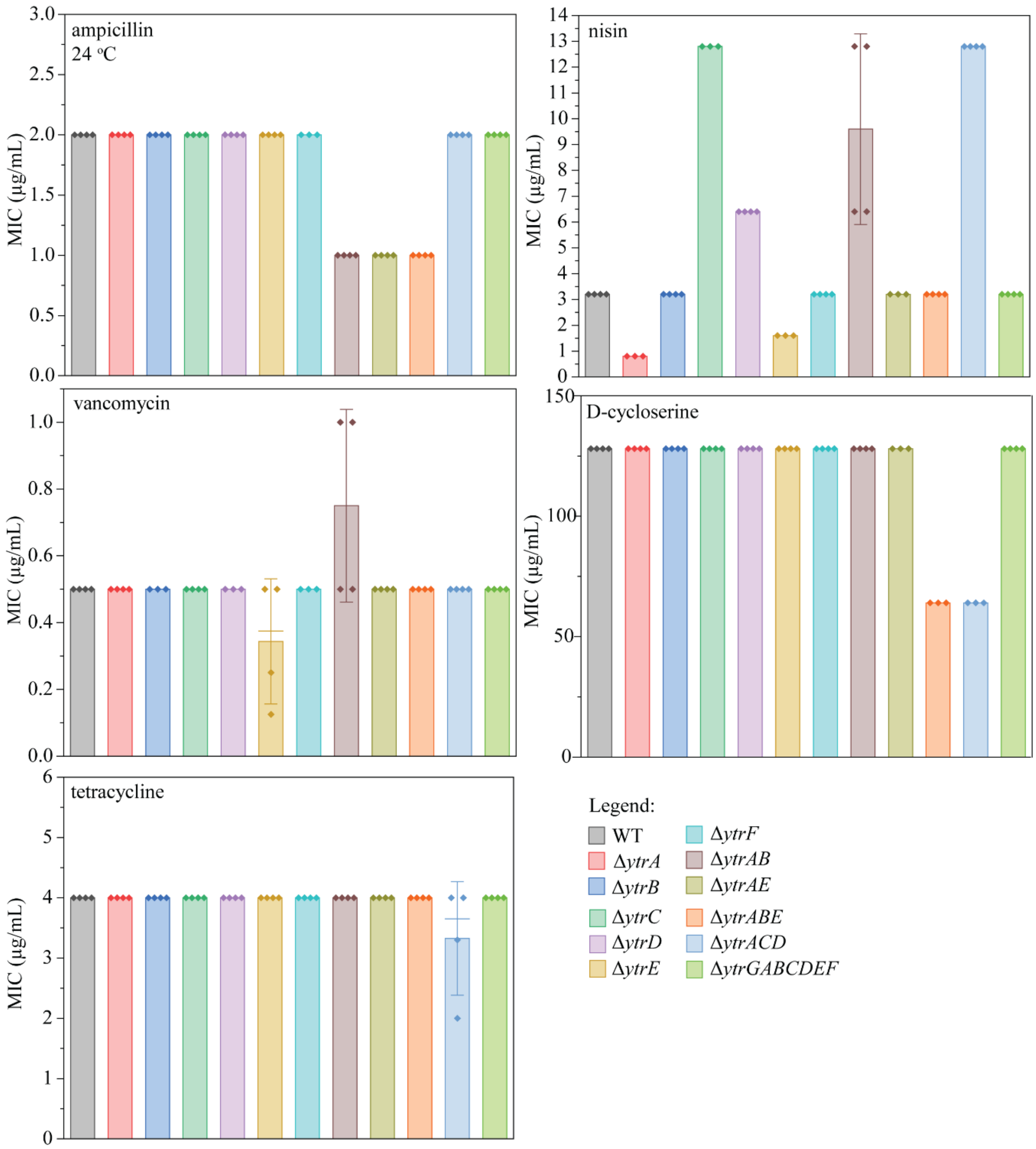

Figure S3: MICs of different antibiotics against *ytr* deletion mutants at 24 °C. Error bars show standard deviation of the mean.

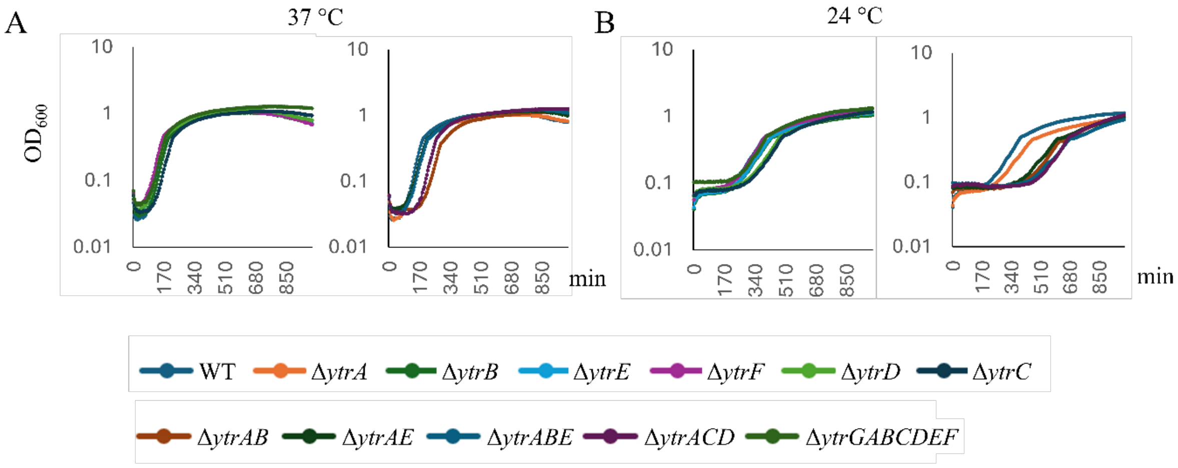

Figure S4: Growth curves of *B. subtilis* 168CA and *ytr* mutants at 37 °C **(A)** and 24 °C **(B)** depicted on a log scale. Left panels show strains not constitutively expressing the operon (Δ*ytrB*, Δ*ytrC*, Δ*ytrD*, Δ*ytrE*, Δ*ytrF*, Δ*ytrGABCDEF*), while the right panels show strains that force-express the whole operon or parts thereof (Δ*ytrA*, Δ*ytrAB*, Δ*ytrAE*, Δ*ytrABE*, Δ*ytrACD*).

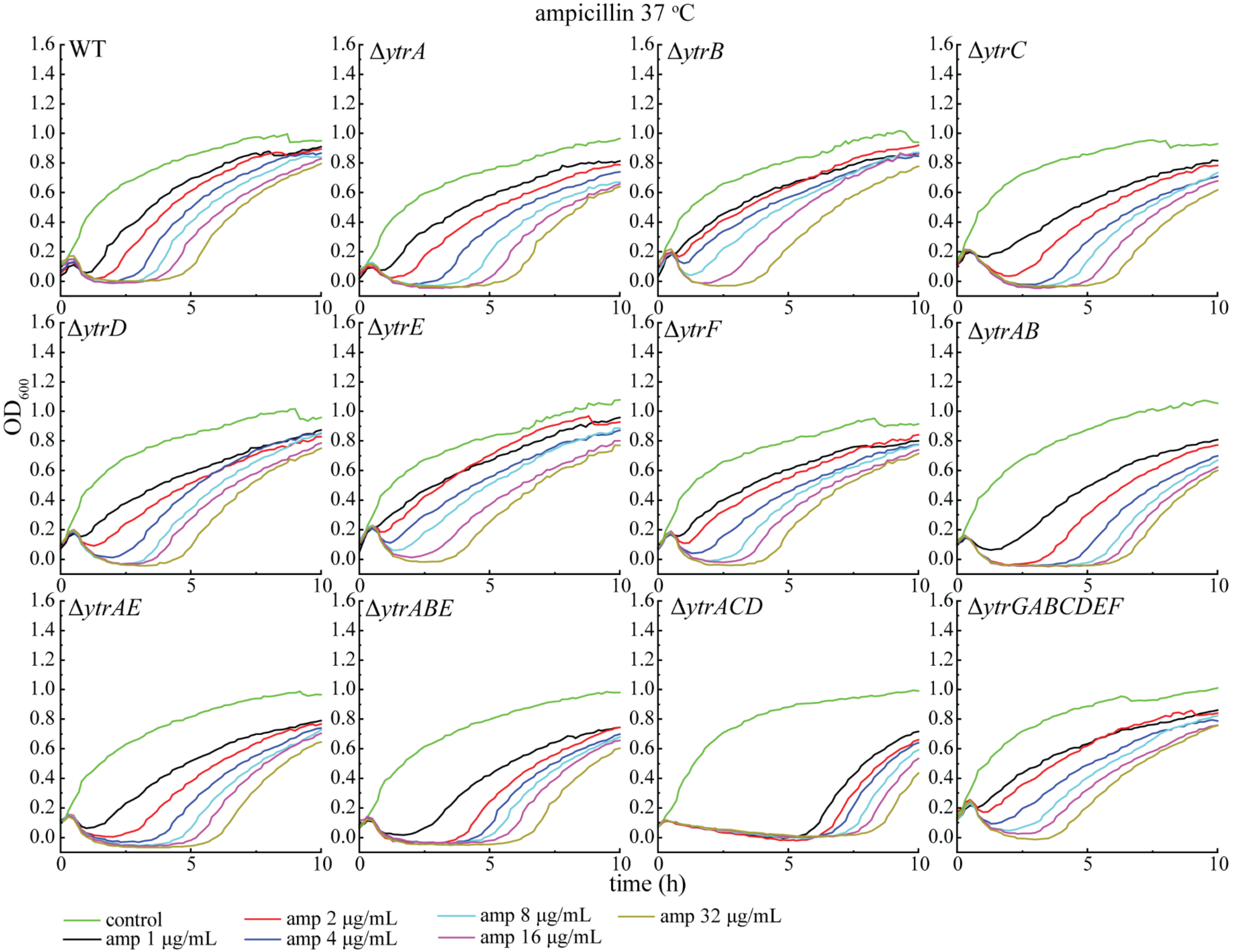

Figure S5: Growth curves of 168CA (WT) and *ytr* deletion mutants after acute shock with different concentrations of ampicillin at 37 °C.

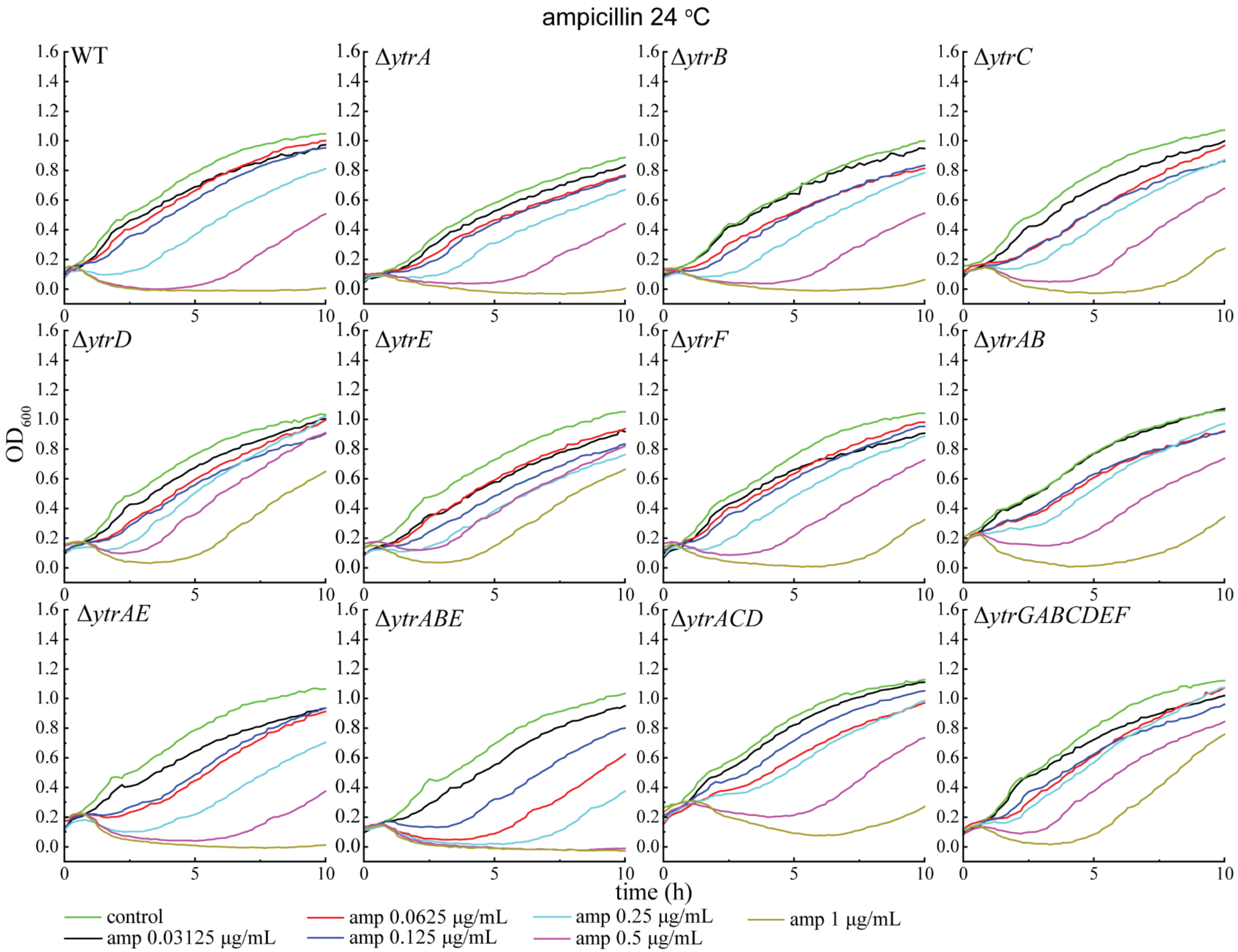

Figure S6: Growth curves of 168CA (WT) and *ytr* deletion mutants after acute shock with different concentrations of ampicillin at 24 °C.

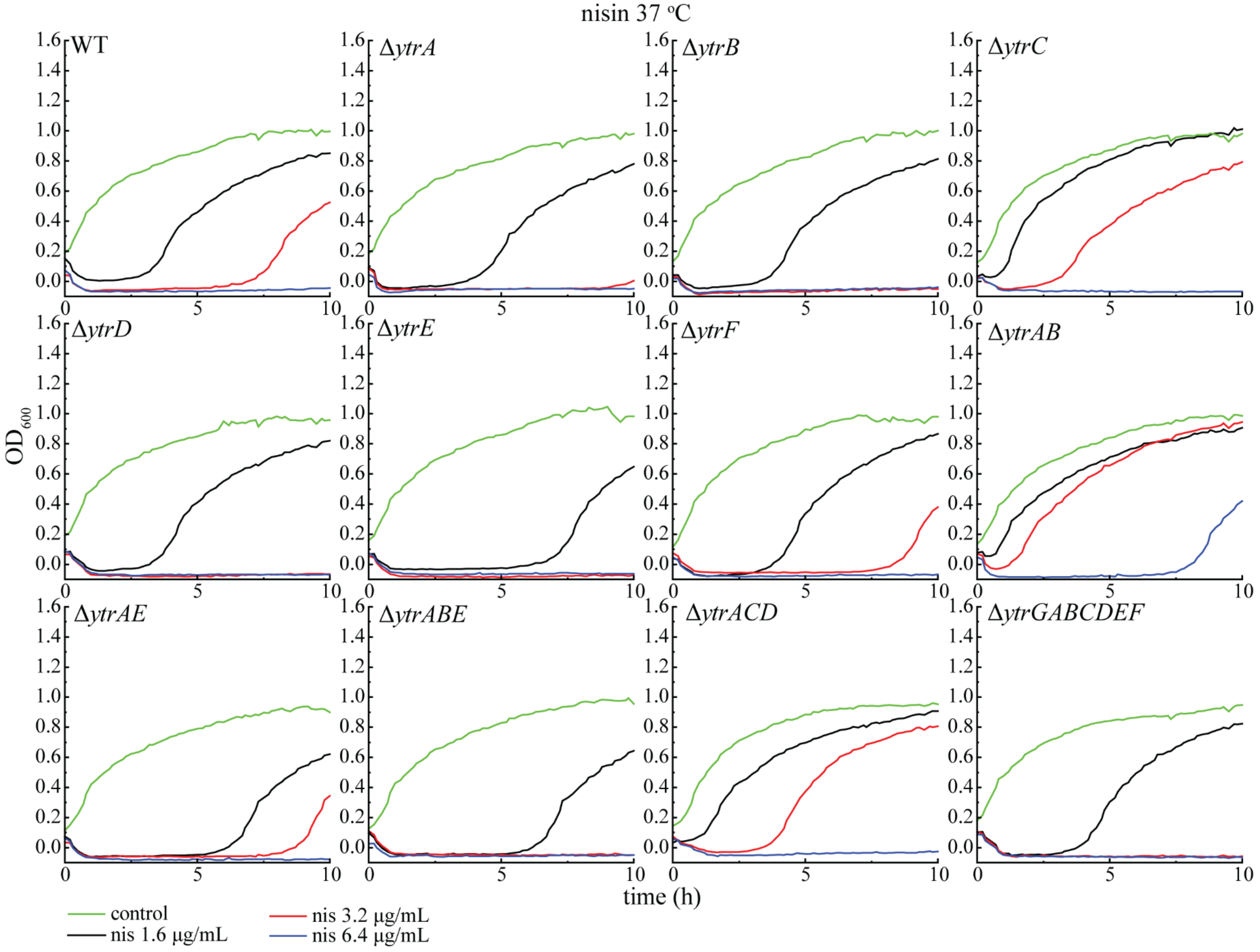

Figure S7: Growth curves of 168CA (WT) and *ytr* deletion mutants after acute shock with different concentrations of nisin at 37 °C.

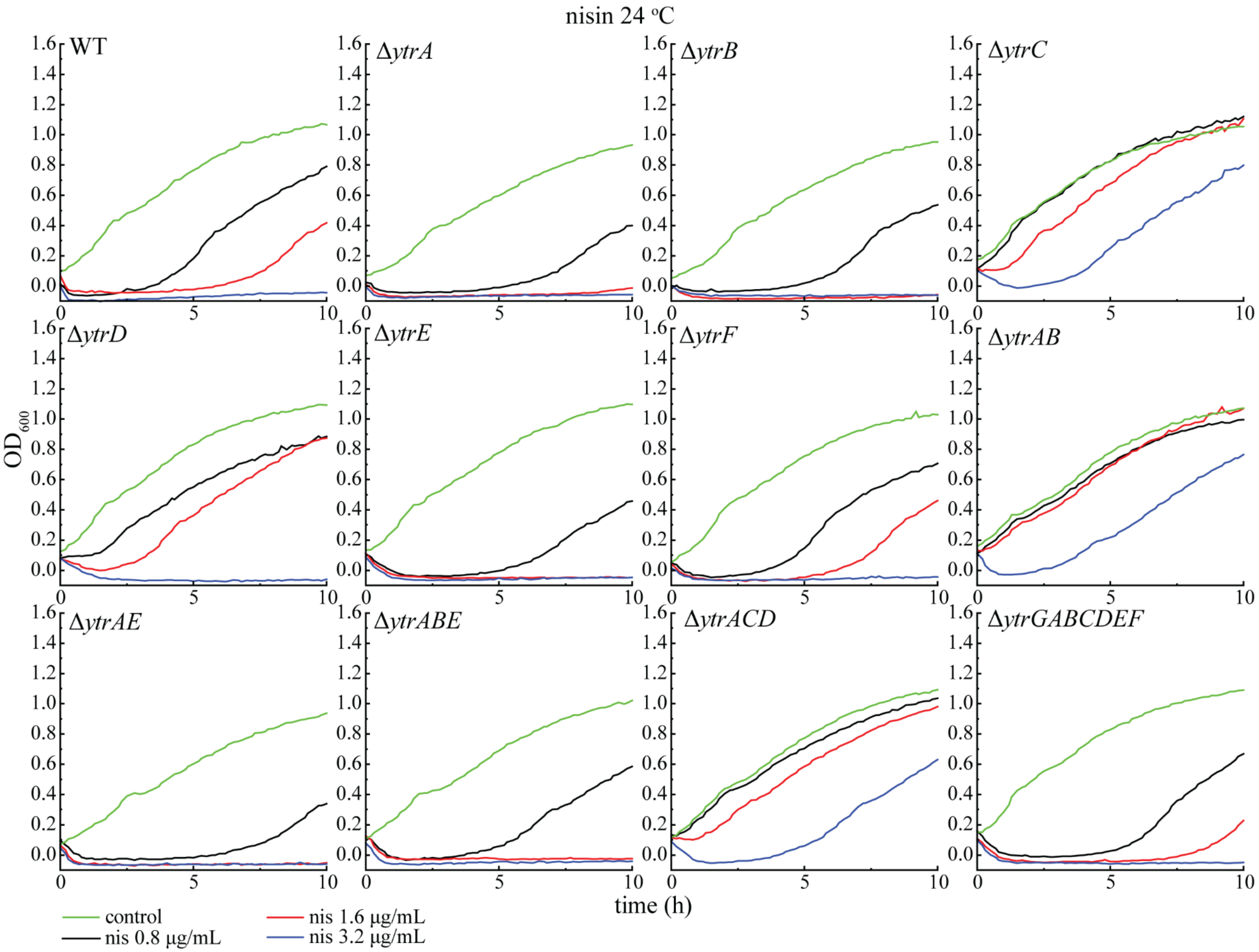

Figure S8: Growth curves of 168CA (WT) and *ytr* deletion mutants after acute shock with different concentrations of nisin at 24 °C.

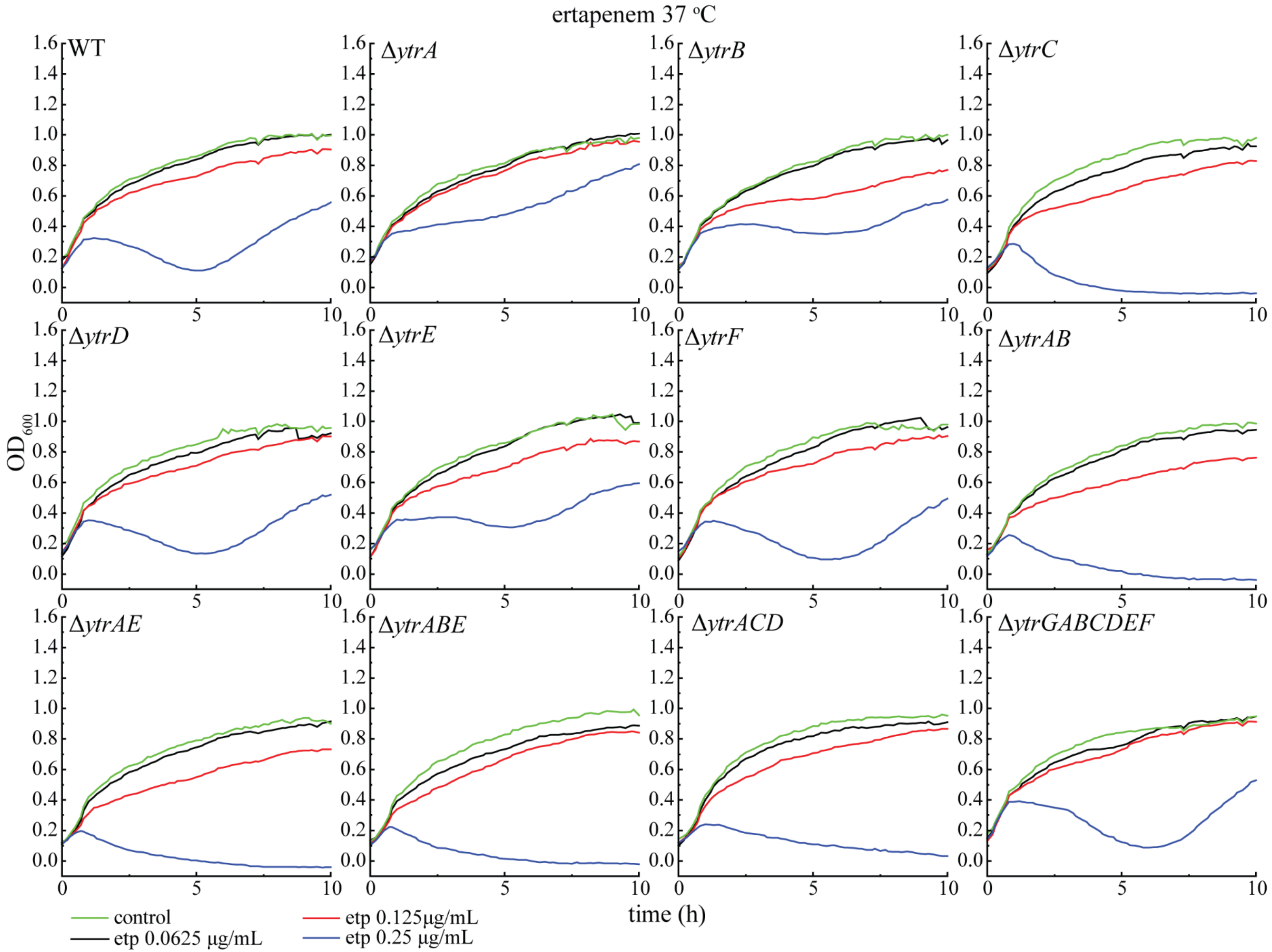

Figure S9: Growth curves of 168CA (WT) and *ytr* deletion mutants after acute shock with different concentrations of ertapenem at 37 °C.

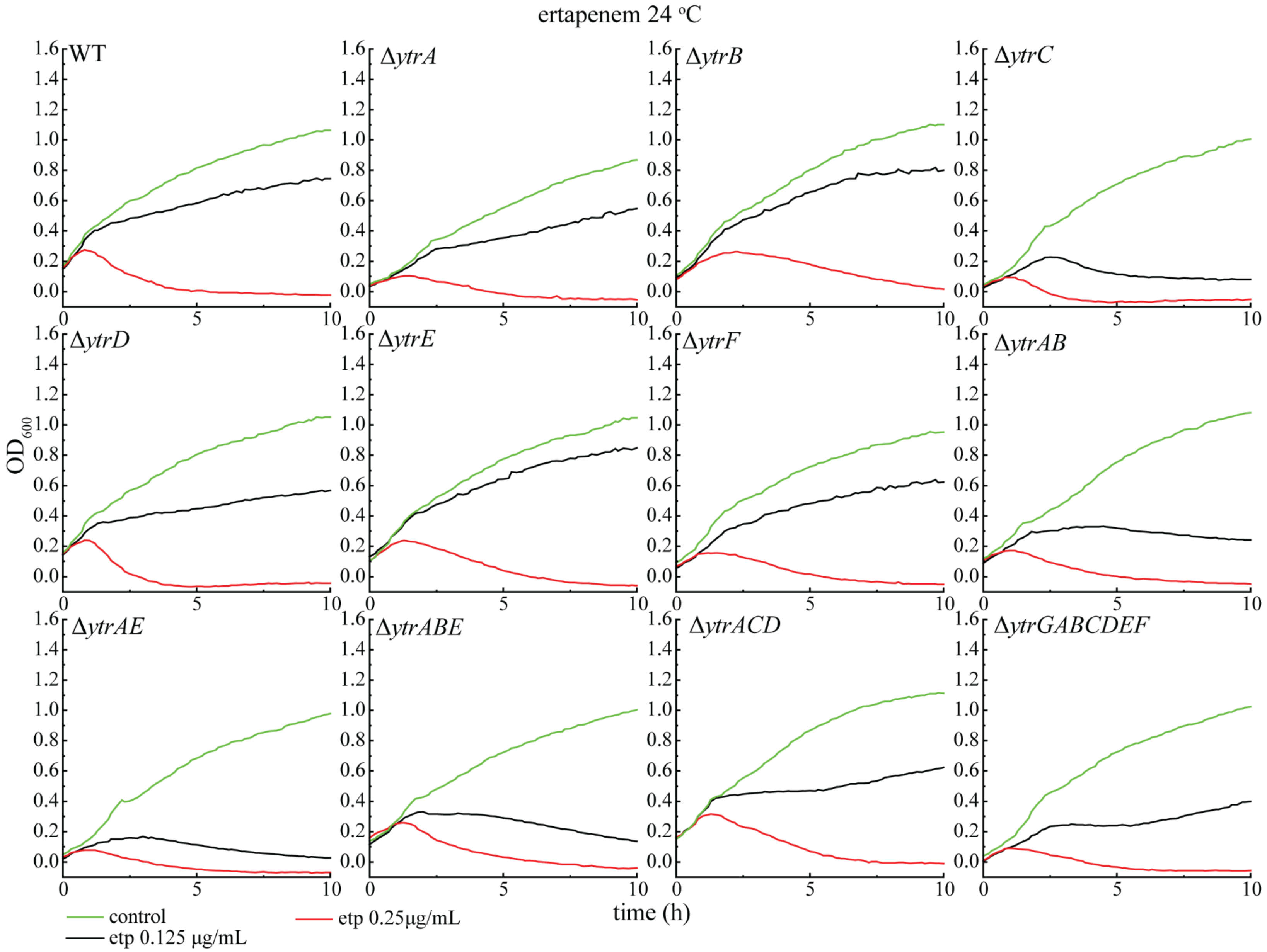

Figure S10: Growth curves of 168CA (WT) and *ytr* deletion mutants after acute shock with different concentrations of ertapenem at 24 °C.

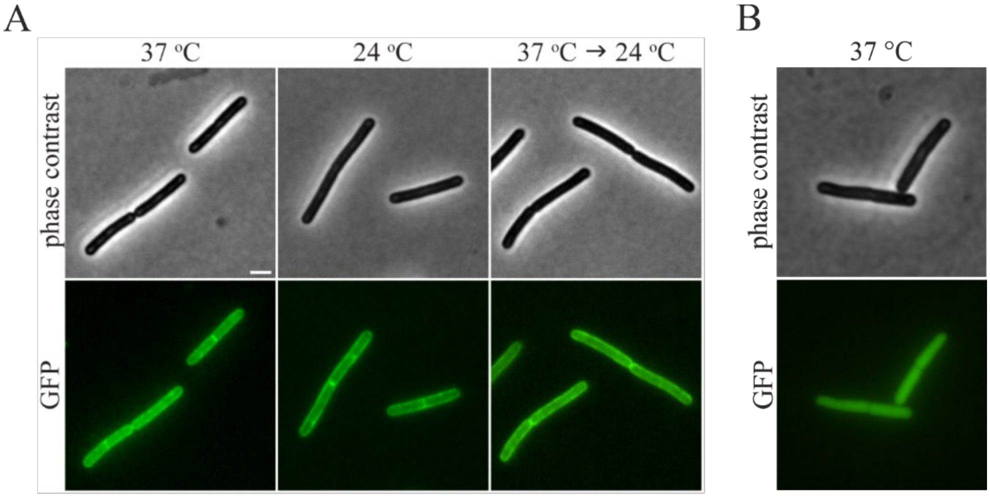

Figure S11: Localization of YtrD-GFP at different temperatures **(A)** and YtrE-GFP **(B)**. *B. subtilis* MS42 (*Pxyl-ytrD-msfgfp*) and PH4 (*Pxyl-ytrE-msfgfp*) were grown at the indicated temperatures until early log phase (OD_{600}=0.3) prior to microscopy. For the temperature shift to 24 ºC, overnight cultures were grown at 37 °C and shifted to 24 °C after dilution in the morning. Scale bar 2 µm.

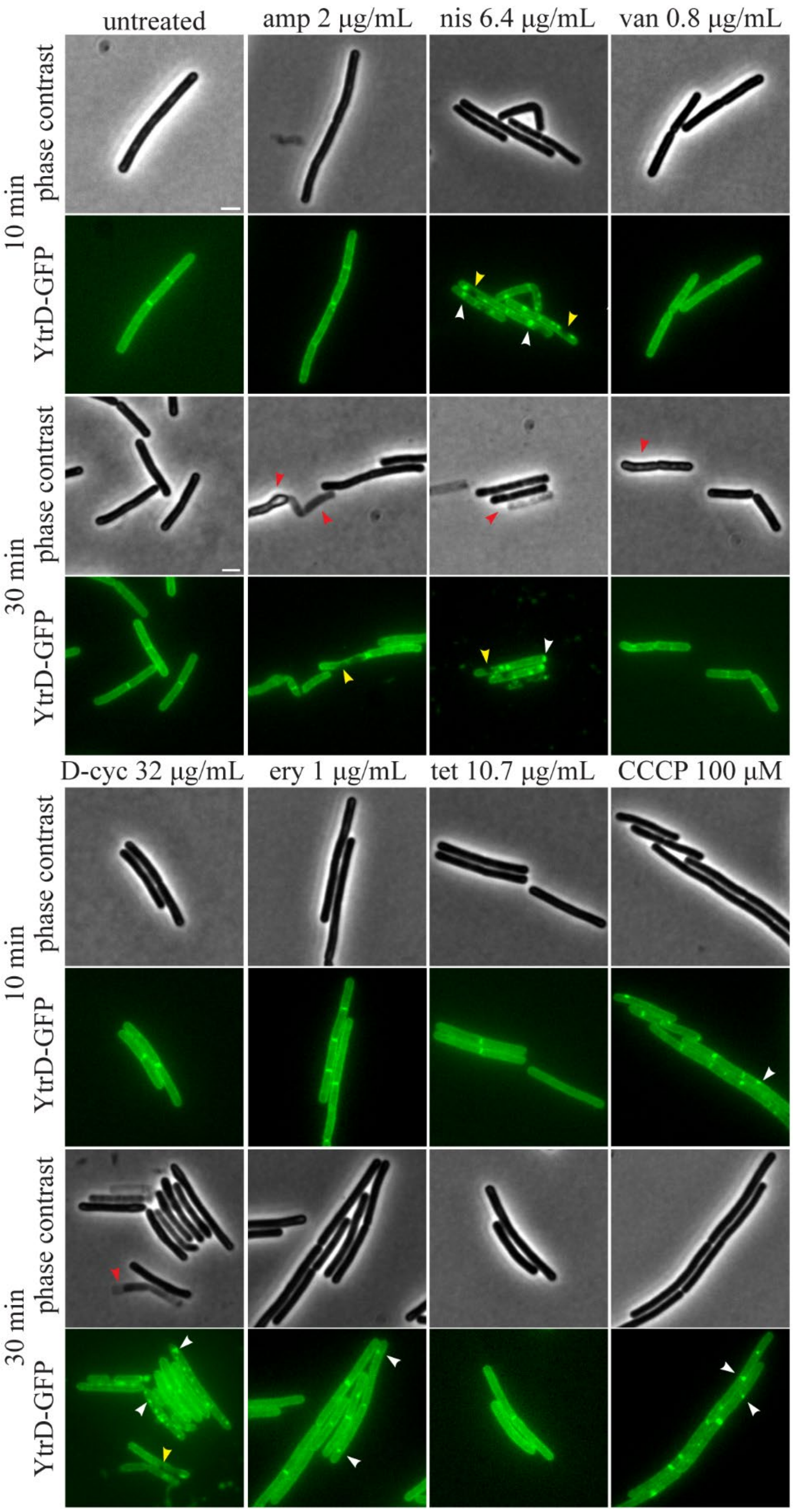

Figure S12: Localization of YtrD under antibiotic stress. *B. subtilis* MS42 (*Pxyl-ytrD-msfgfp*) was grown at 37 °C until an OD_{600} of 0.3 and subsequently treated with antibiotics (amp: ampicillin, nis: nisin; van: vancomycin, D-cyc: D-cycloserine, ery: erythromycin, tet: tetracycline, CCCP: carbonyl cyanide m-chlorophenyl hydrazone). Arrow legend: GFP clusters (white), area void of GFP (orange), cell deformation or lysis (Red). Scale bar 2 µm.

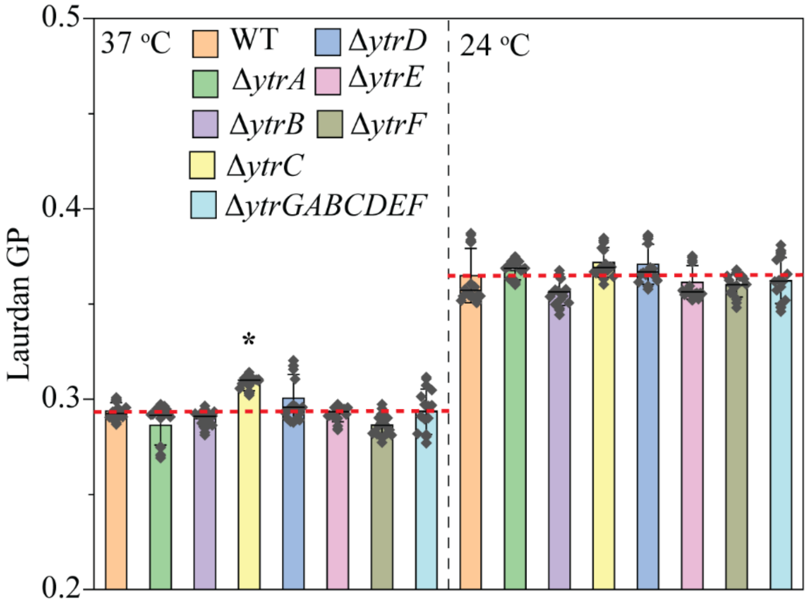

Figure S13: Laurdan spectroscopy of deletion mutants at 37 °C and 24 °C. Red line marks the average of the wild type. Statistical significance ($p<0.05$) was tested with a two-tailed heteroscedastic t-test. Only Δ*ytrC* showed a significant difference to the wild type ($p=0.017$), yet only at 37 °C.

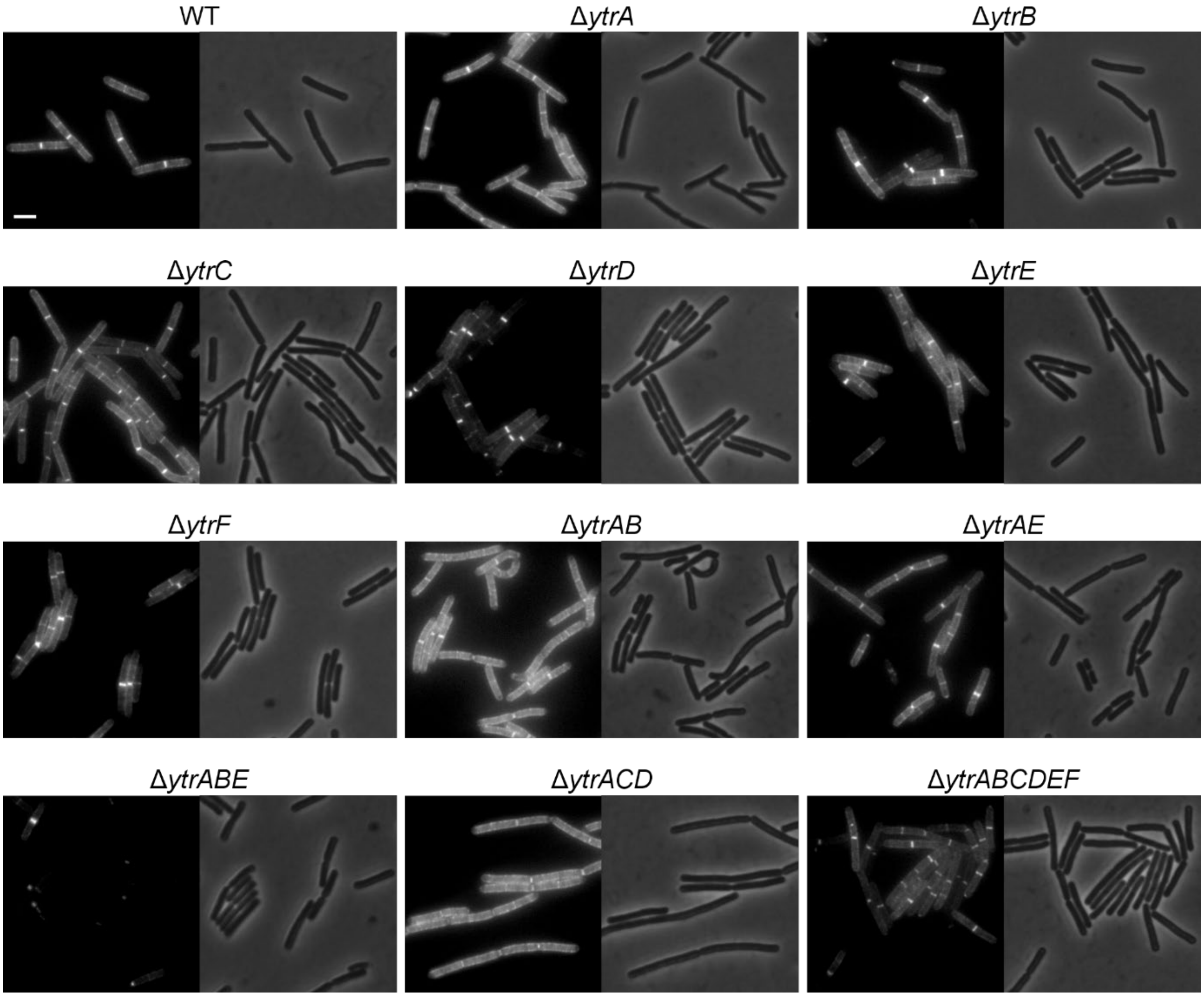

Figure S14: Microscopy images of *B. subtilis* 168CA (WT) and *ytr* mutants labeled with Van-FL. Cells were grown at 37 °C. Exposure times, light intensity, and brightness/contrast settings were identical for all samples. Scale bar 2 µm.

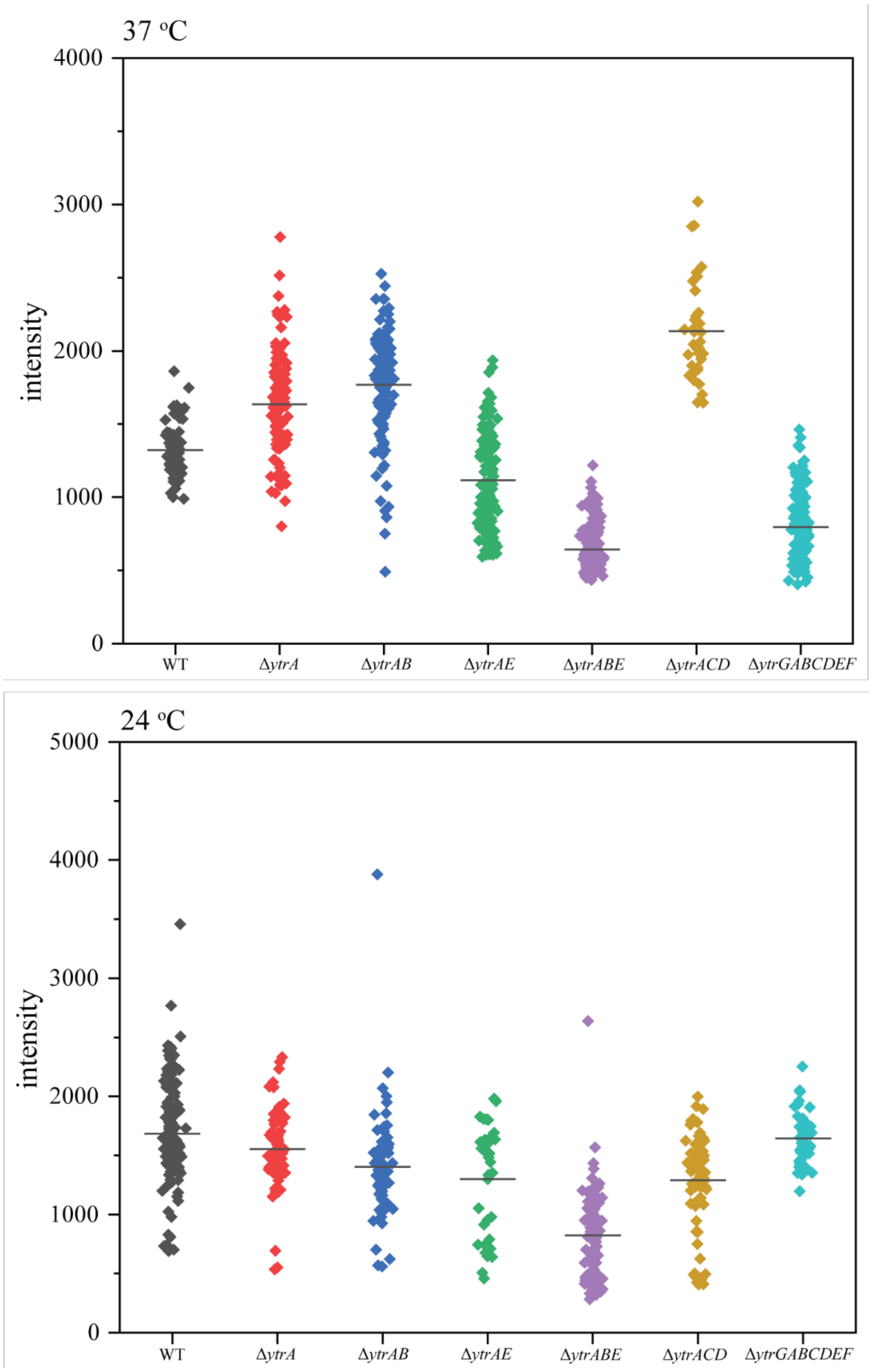

Figure S15: Quantification of whole cell fluorescence from microscopy images of *B. subtilis* strains labeled with Van-FL.

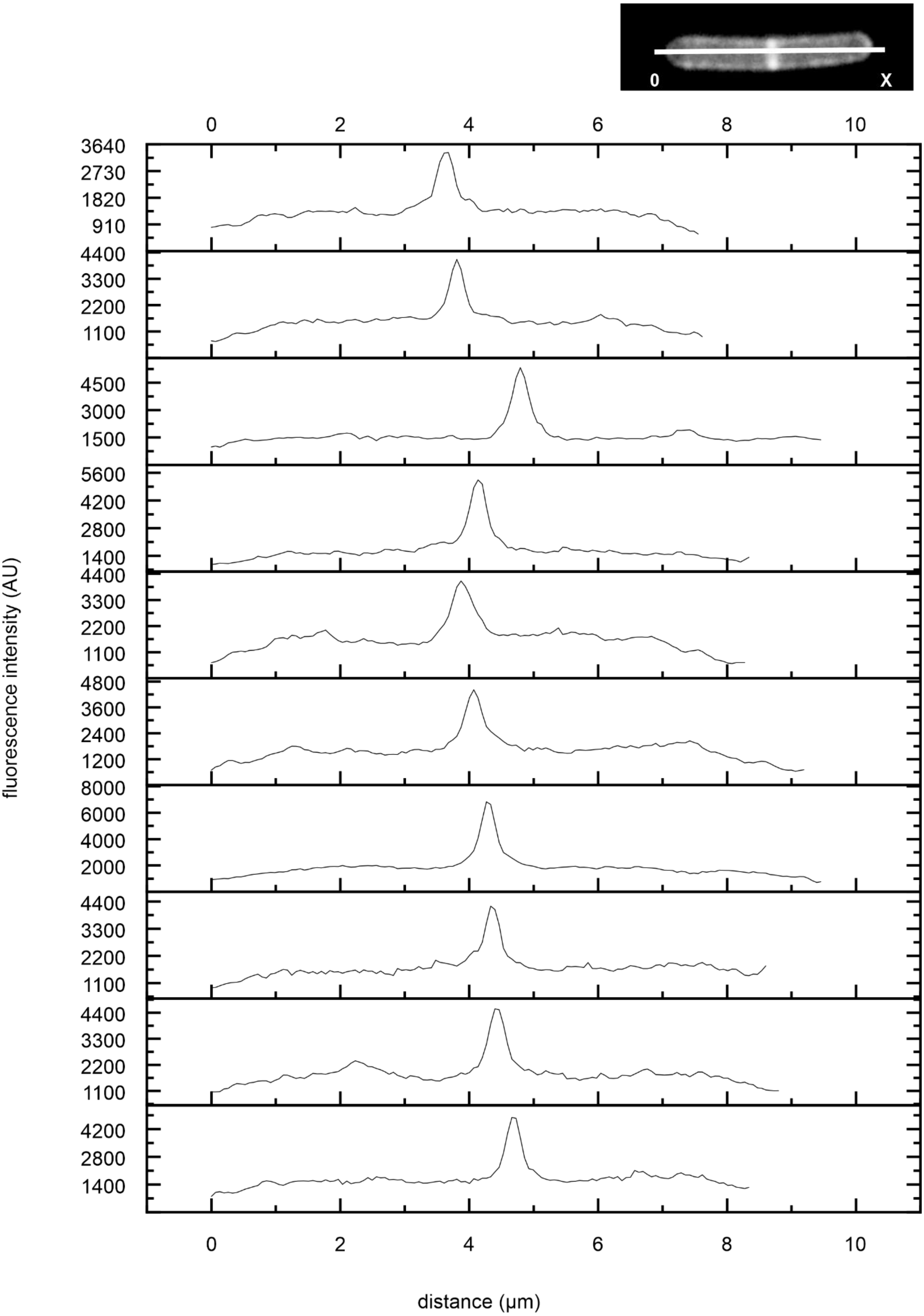

Figure S16: Longitudinal line scans of individual *B. subtilis* 168CA (WT) cells grown at 37 °C and stained with Van-FL in exponential growth phase. Longitudinal line scans were drawn through the middle of the cell to measure septal accumulation of Van-FL signal.

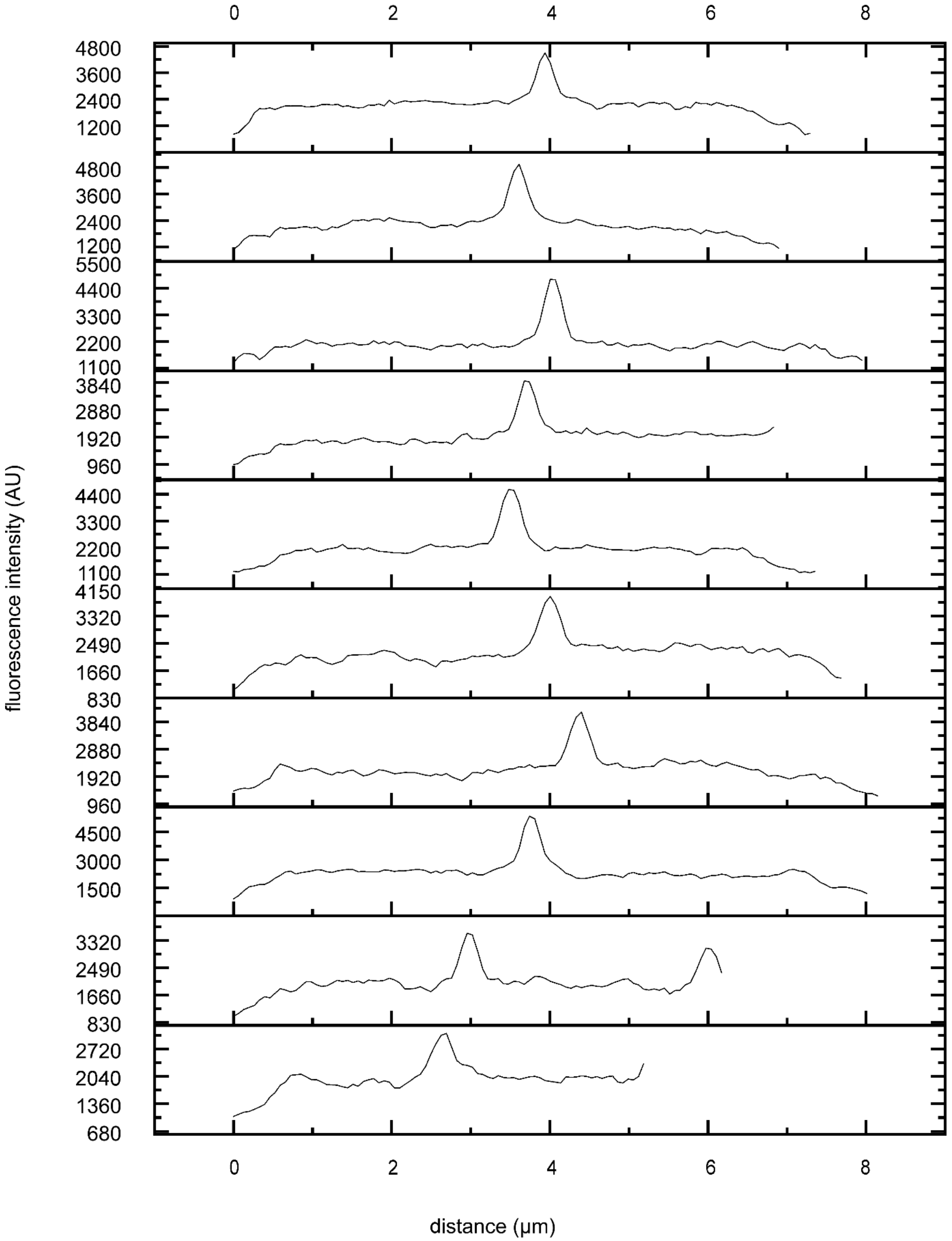

Figure S17: Longitudinal line scans of individual *B. subtilis* PH5 (Δ*ytrA*) cells grown at 37 °C and stained with Van-FL in exponential growth phase.

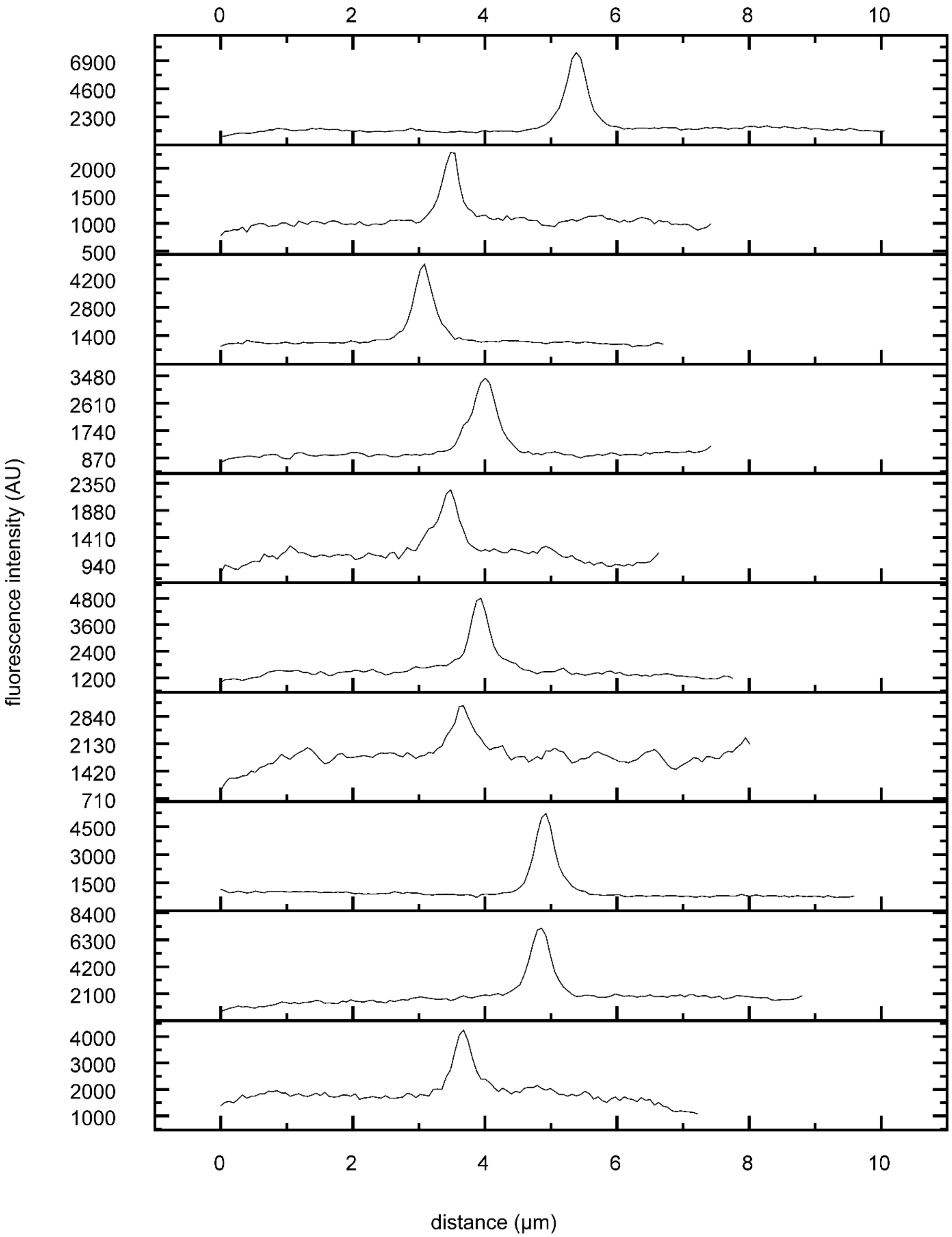

Figure S18: Longitudinal line scans of individual *B. subtilis* PH1 (Δ*ytrB*) cells grown at 37 °C and stained with Van-FL in exponential growth phase.

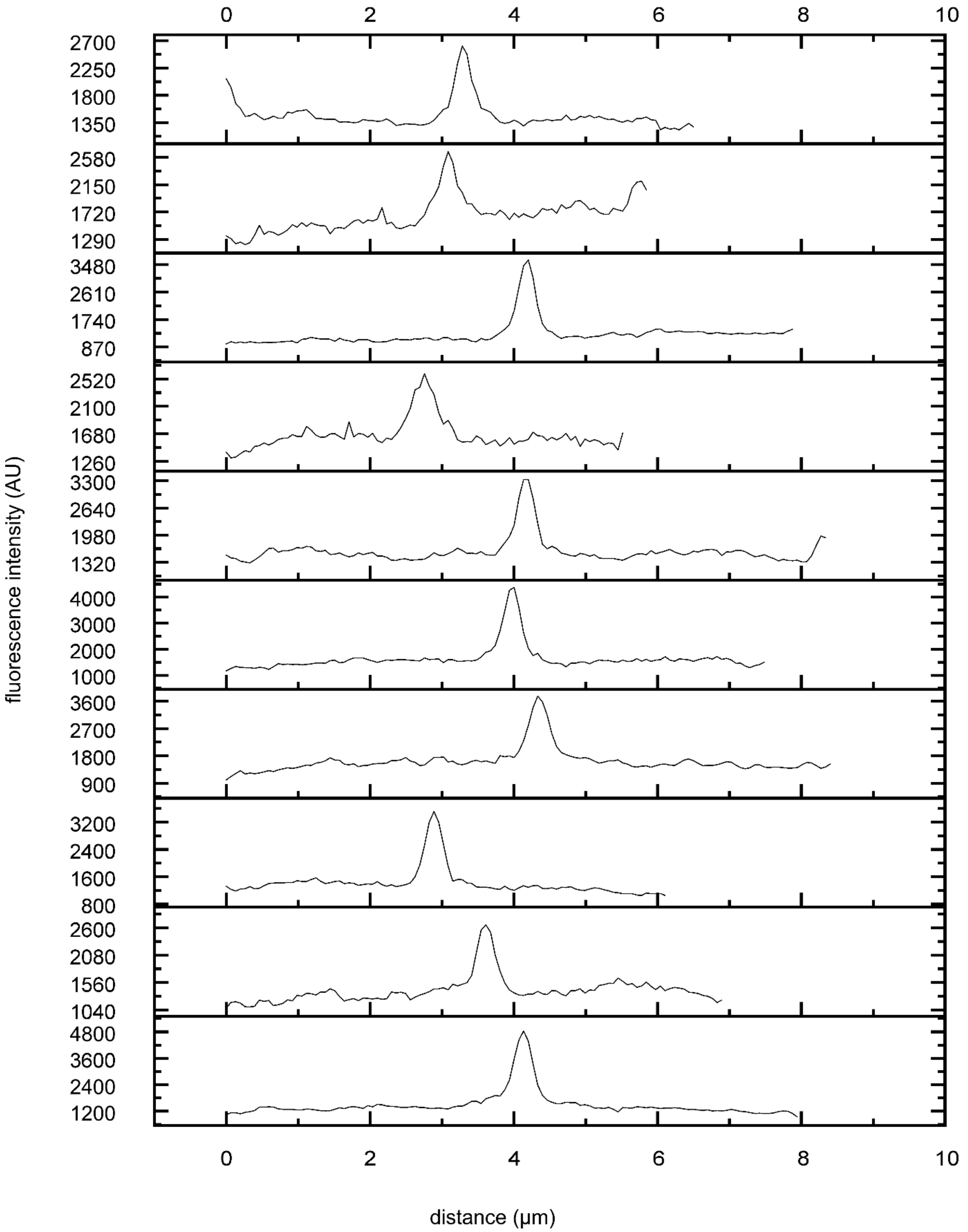

Figure S19: Longitudinal line scans of individual *B. subtilis* PD3 (Δ*ytrC*) cells grown at 37 °C and stained with Van-FL in exponential growth phase.

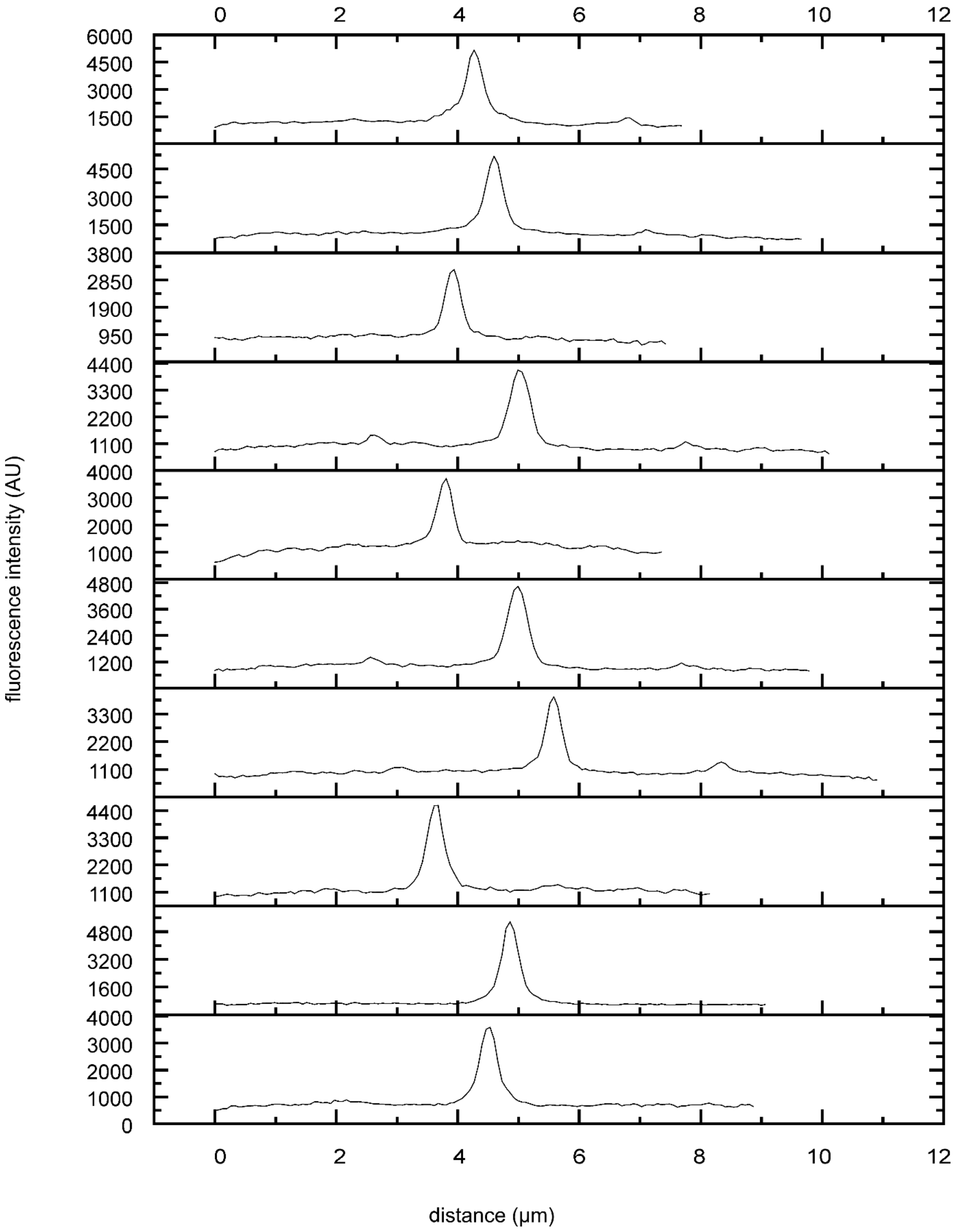

Figure S20: Longitudinal line scans of individual *B. subtilis* PD2 (Δ*ytrD*) cells grown at 37 °C and stained with Van-FL in exponential growth phase.

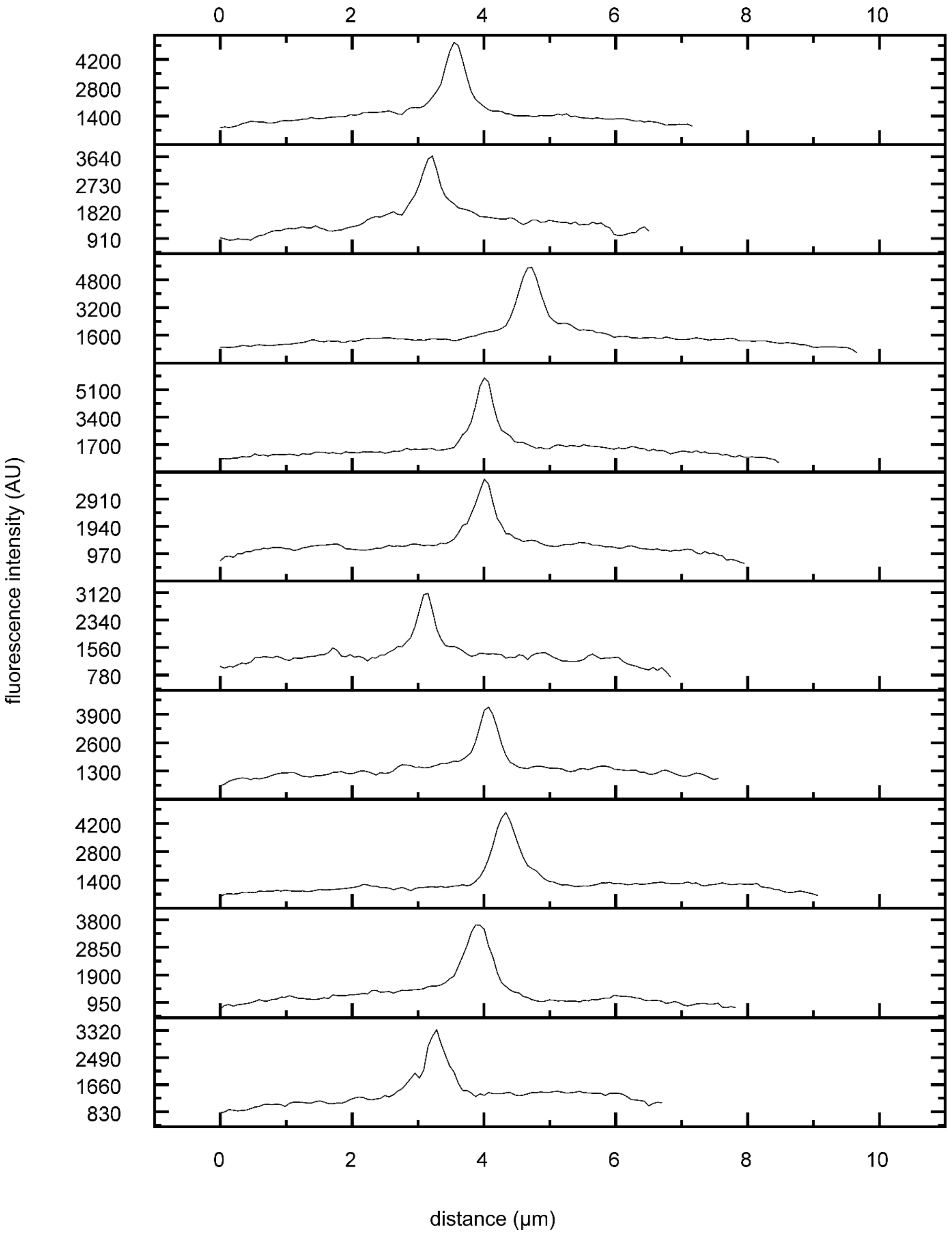

Figure S21: Longitudinal line scans of individual *B. subtilis* PH2 (Δ*ytrE*) cells grown at 37 °C and stained with Van-FL in exponential growth phase.

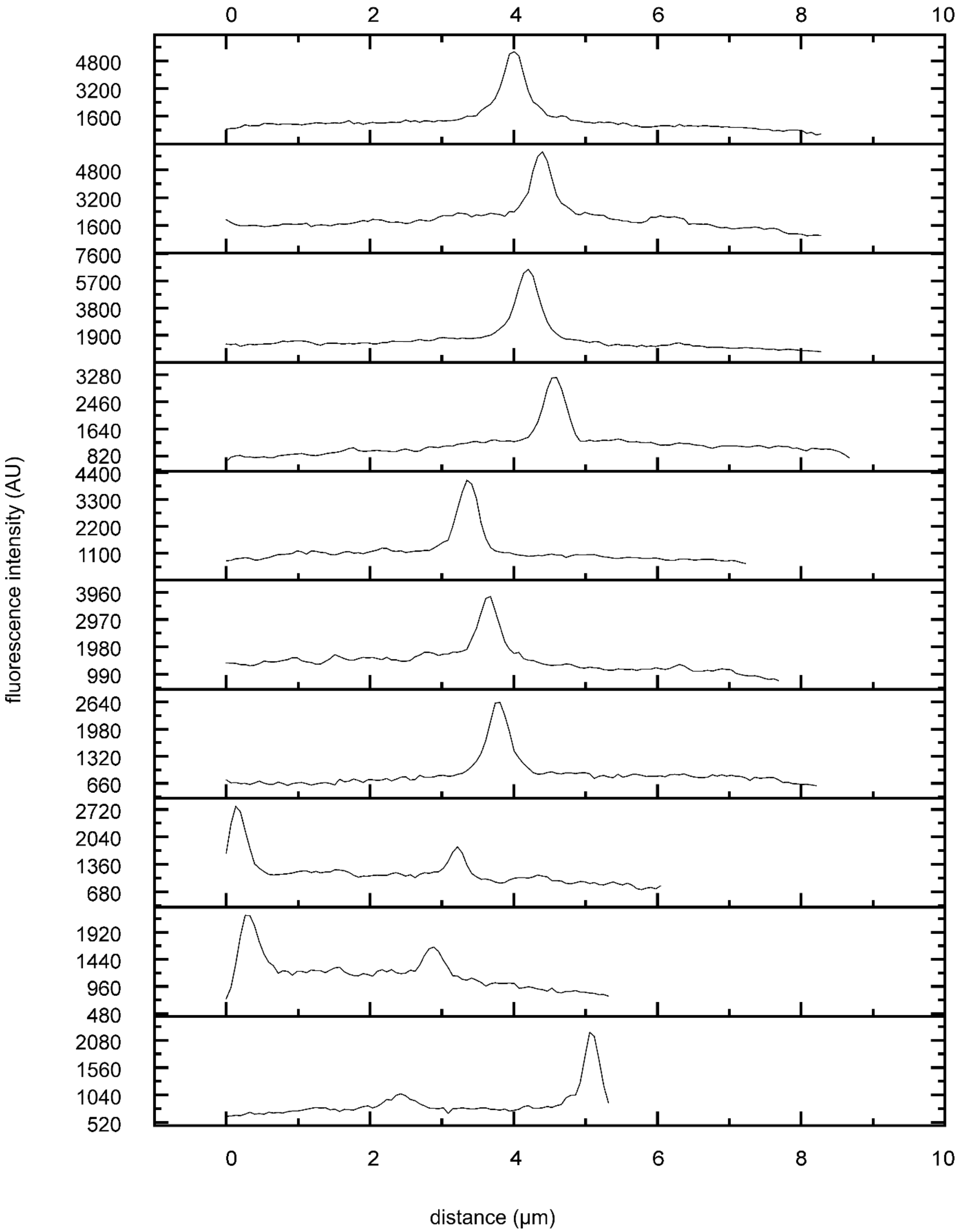

Figure S22: Longitudinal line scans of individual *B. subtilis* PD1 (Δ*ytrF*) cells grown at 37 °C and stained with Van-FL in exponential growth phase.

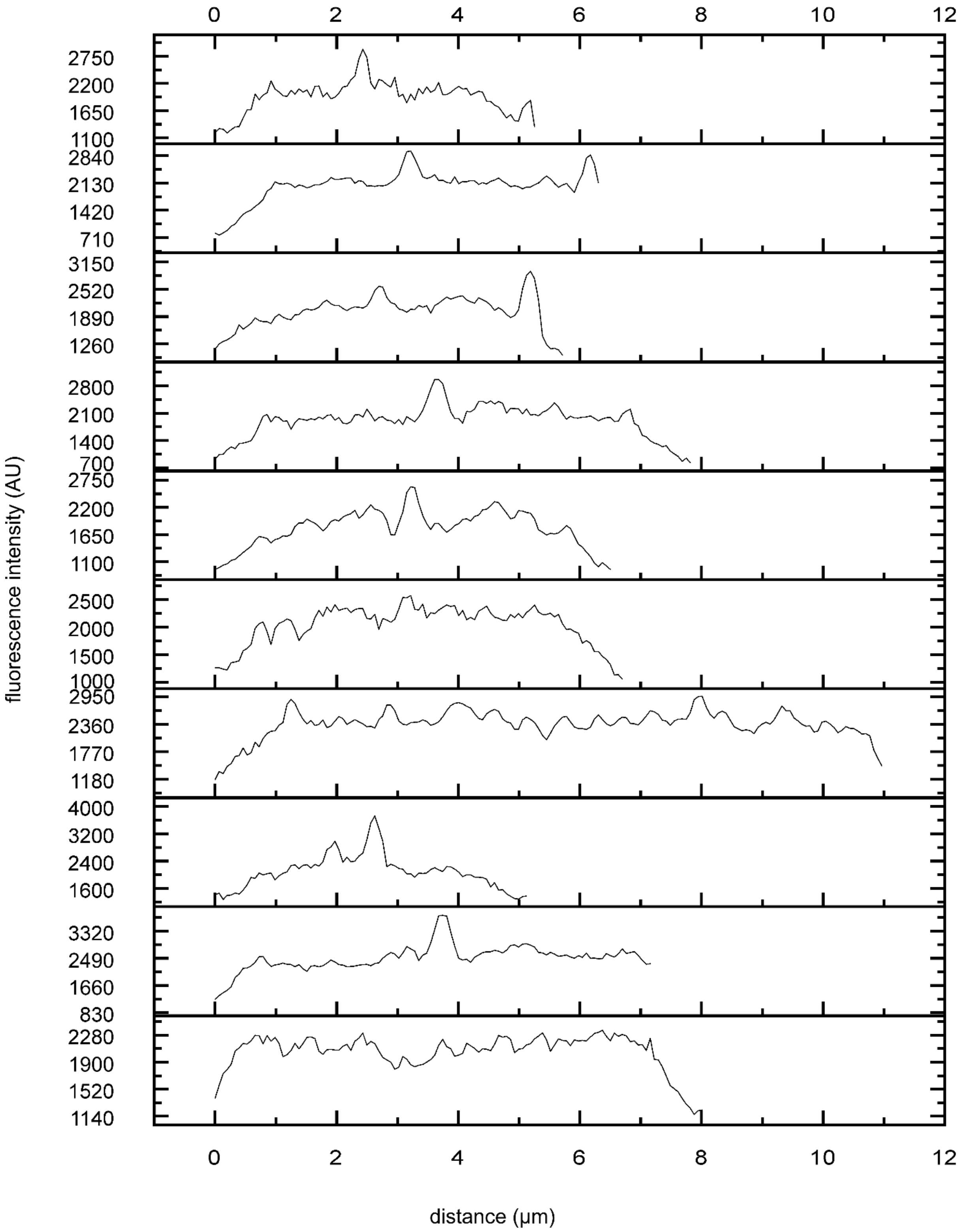

Figure S23: Longitudinal line scans of individual *B. subtilis* GP3193 (Δ*ytrAB*) cells grown at 37 °C and stained with Van-FL in exponential growth phase.

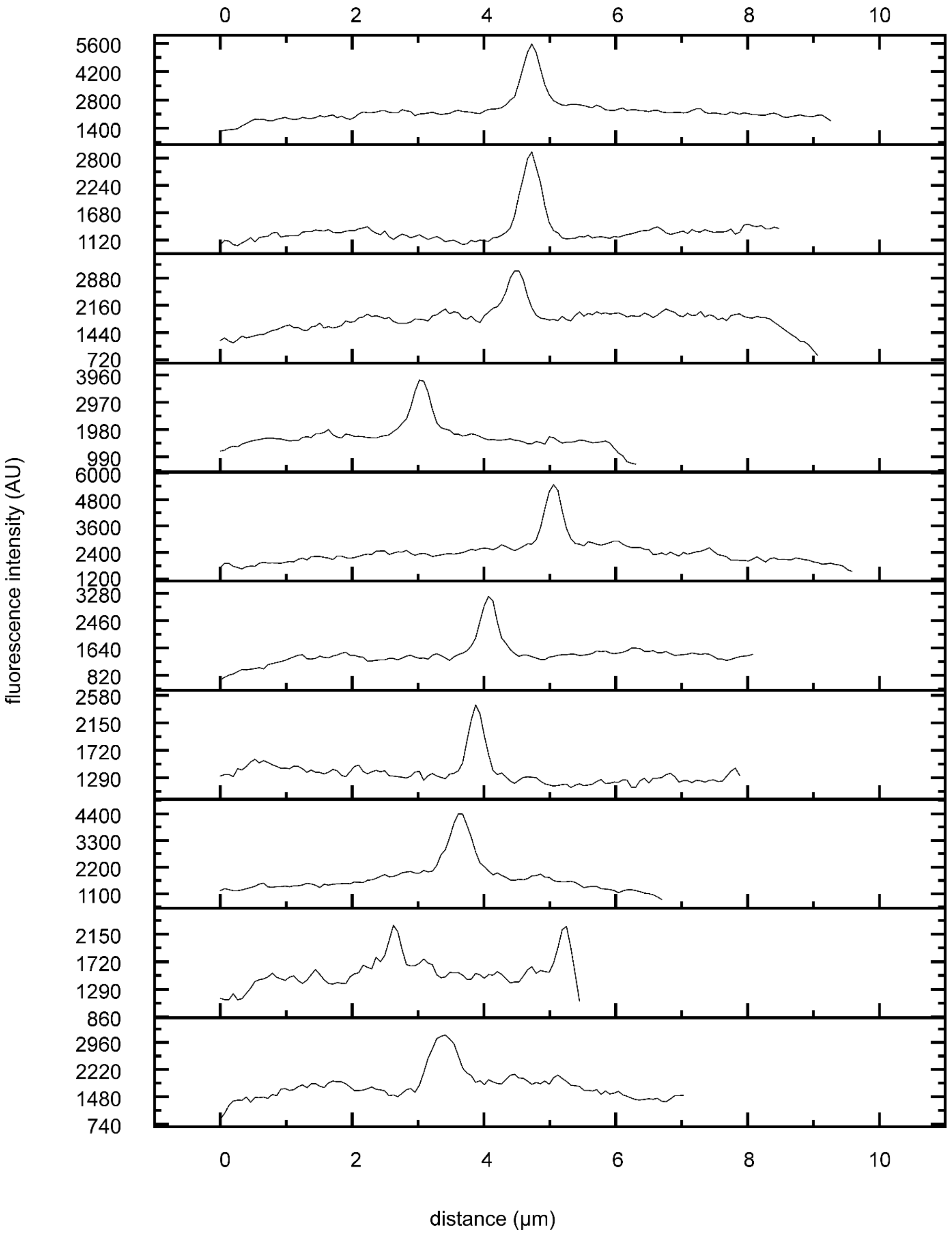

Figure S24: Longitudinal line scans of individual *B. subtilis* GP3196 (Δ*ytrAE*) cells grown at 37 °C and stained with Van-FL in exponential growth phase.

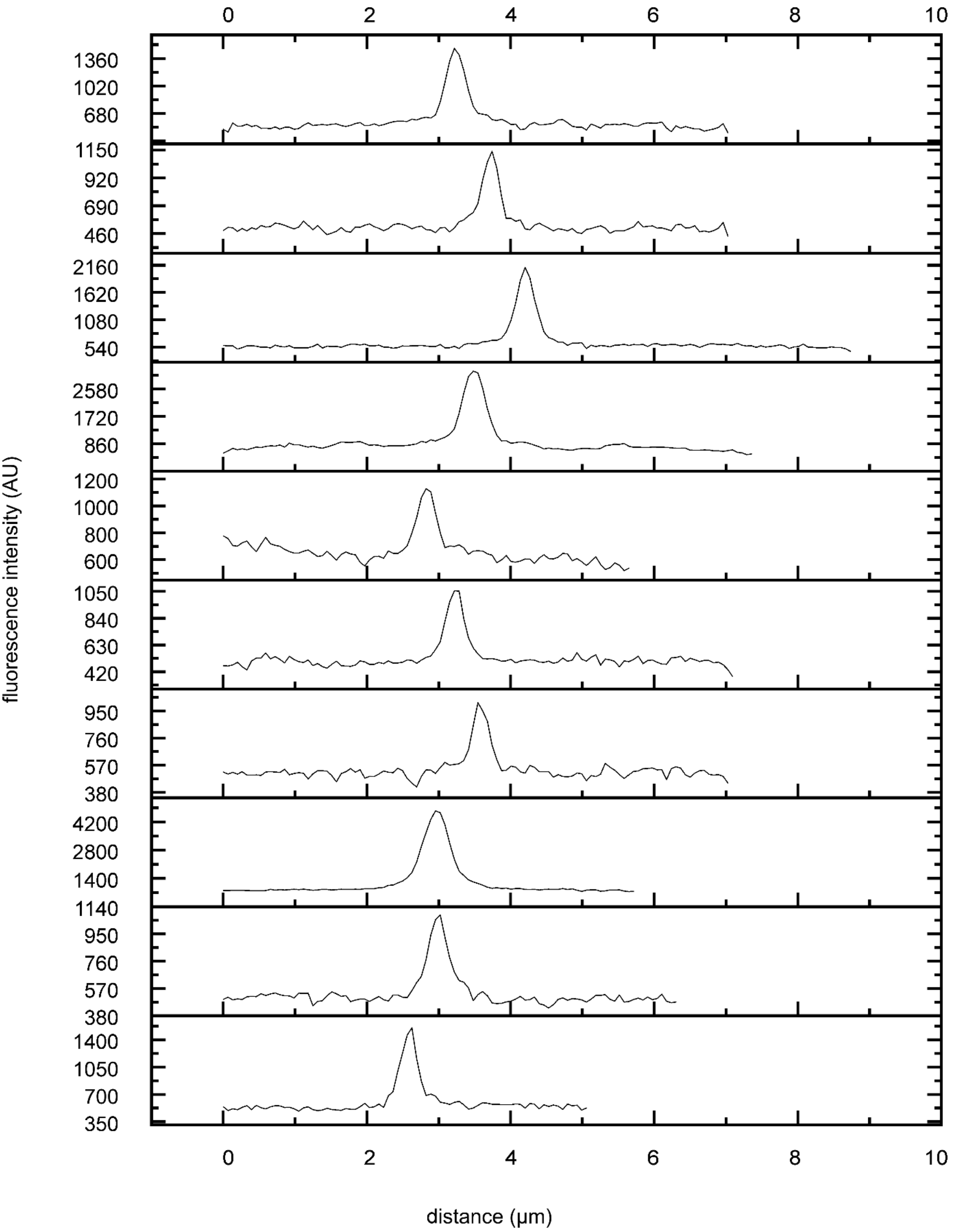

Figure S25: Longitudinal line scans of individual *B. subtilis* GP3206 (Δ*ytrABE*) cells grown at 37 °C and stained with Van-FL in exponential growth phase.

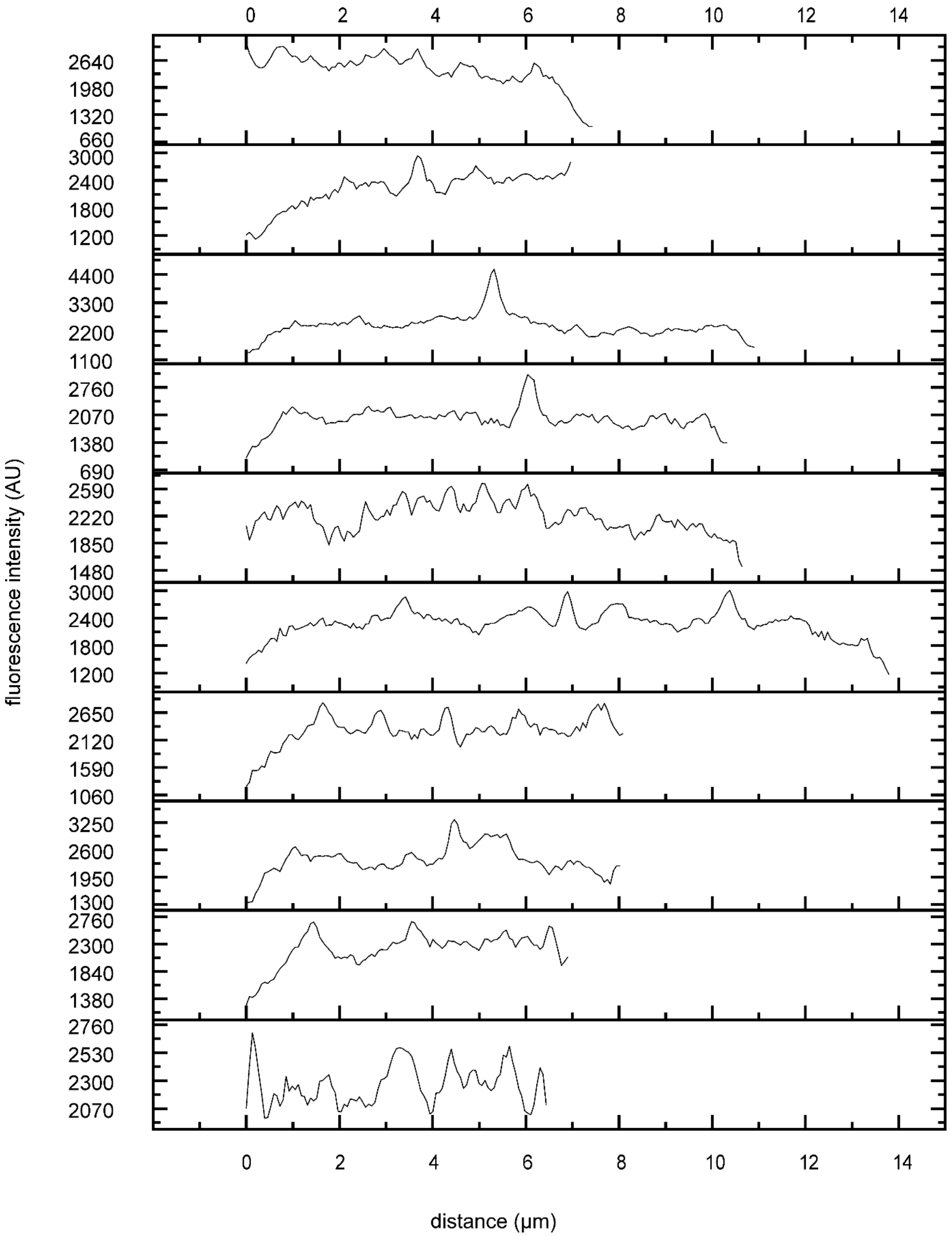

Figure S26: Longitudinal line scans of individual *B. subtilis* BLMS3 (Δ*ytrACD*) cells grown at 37 °C and stained with Van-FL in exponential growth phase.

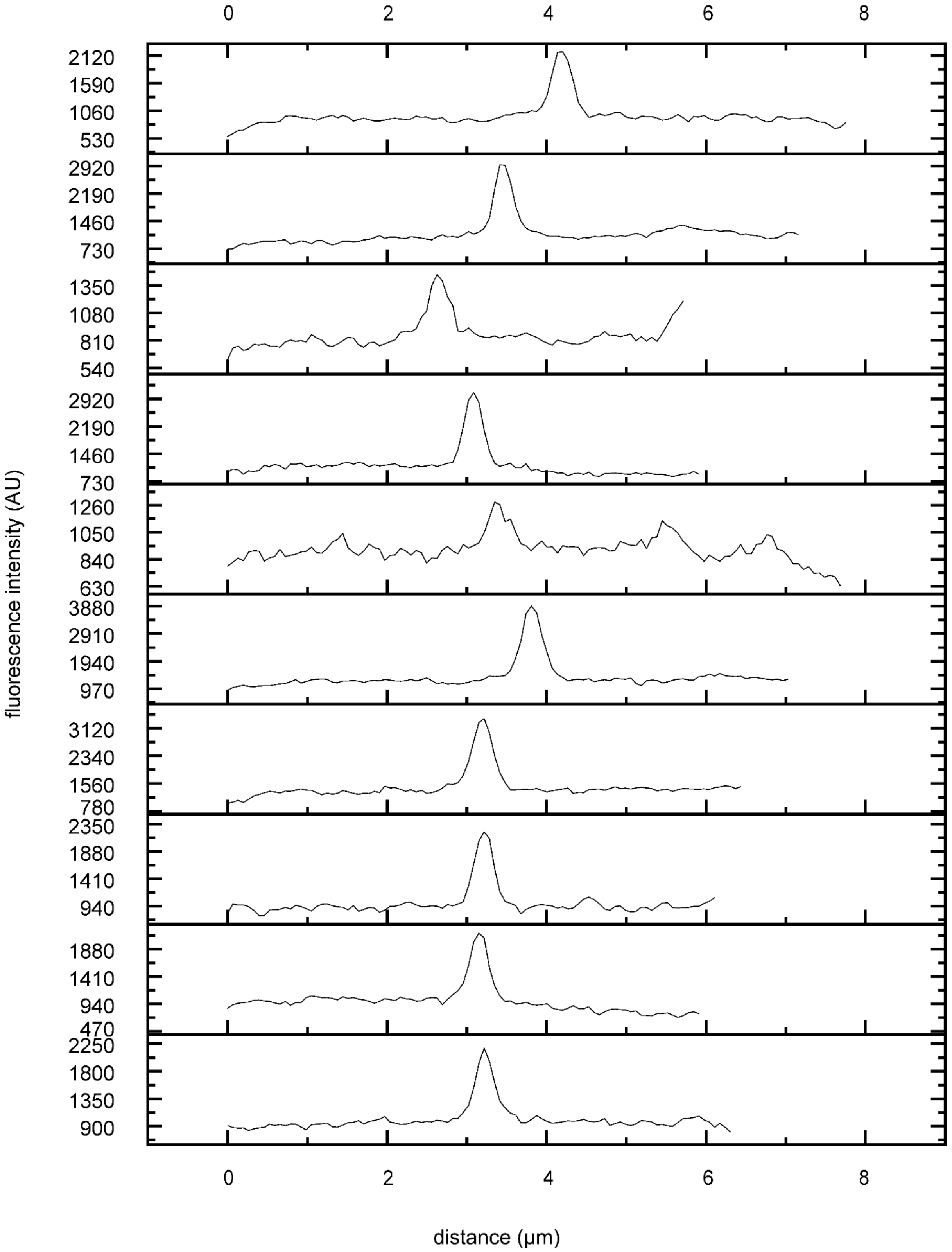

Figure S27: Longitudinal line scans of individual *B. subtilis* GP2646 (Δ*ytrGABCDEF*) cells grown at 37 °C and stained with Van-FL in exponential growth phase.

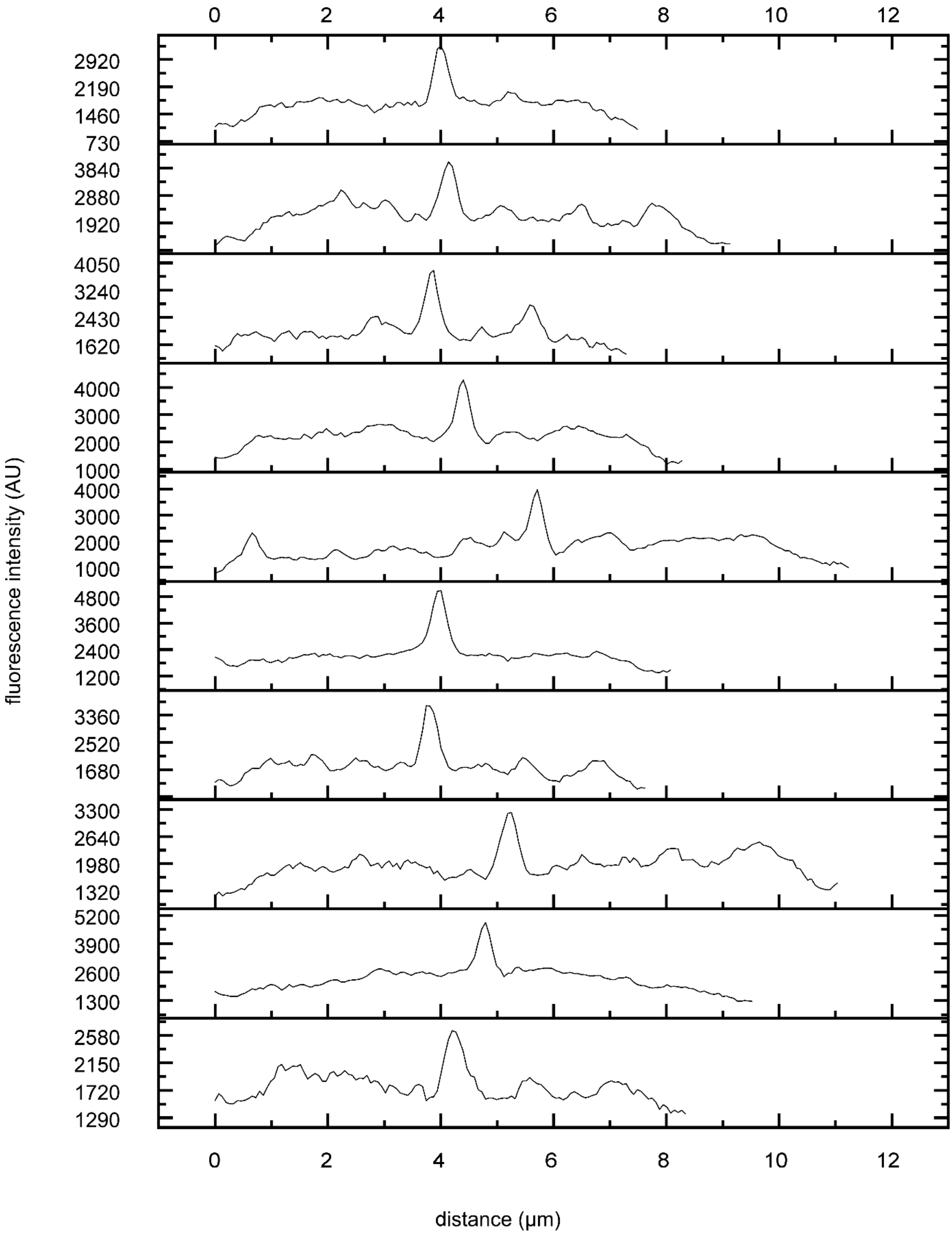

Figure S28: Longitudinal line scans of individual *B. subtilis* 168CA (WT) cells grown at 24 °C and stained with Van-FL in exponential growth phase.

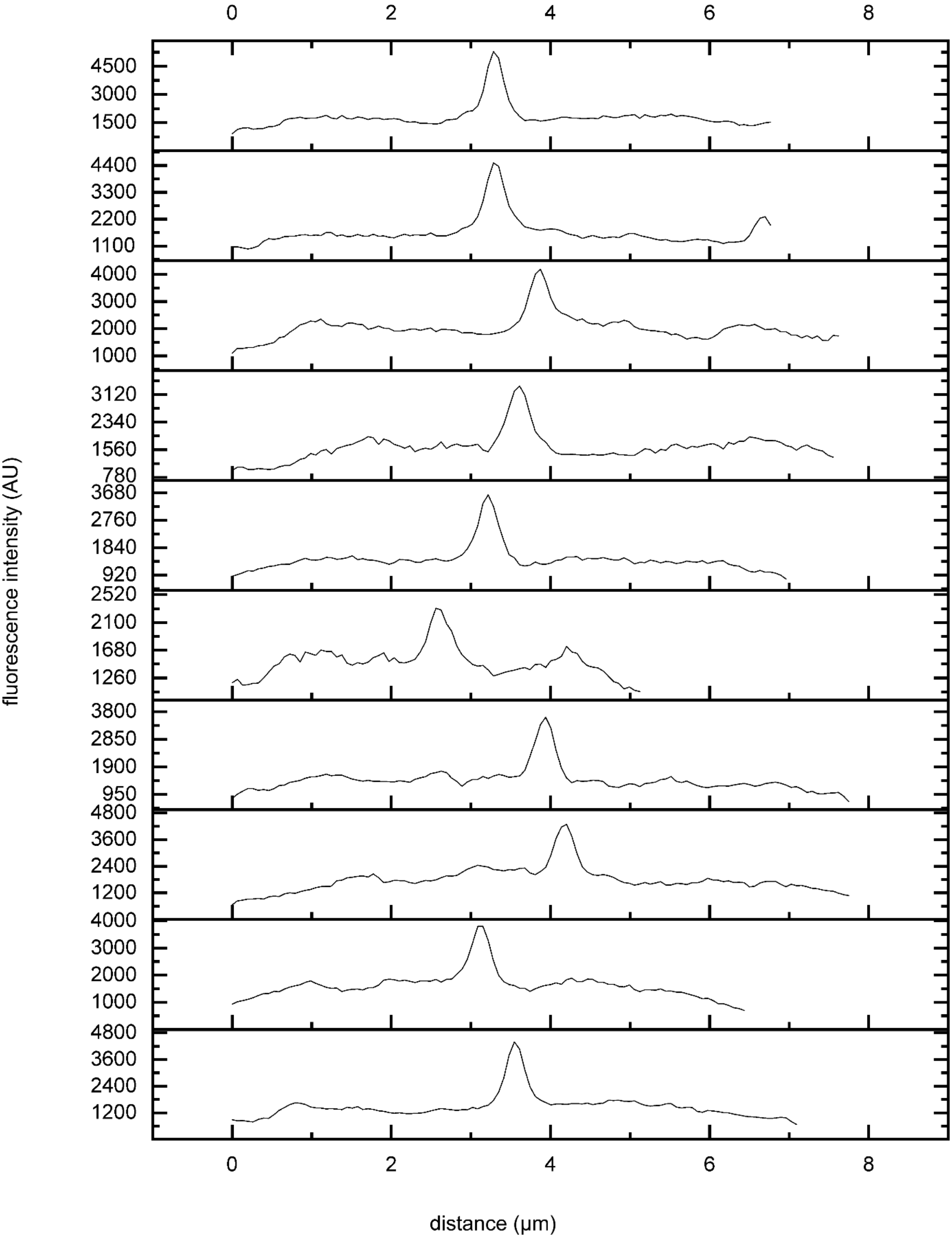

Figure S29: Longitudinal line scans of individual *B. subtilis* PH5 (Δ*ytrA*) cells grown at 24 °C and stained with Van-FL in exponential growth phase.

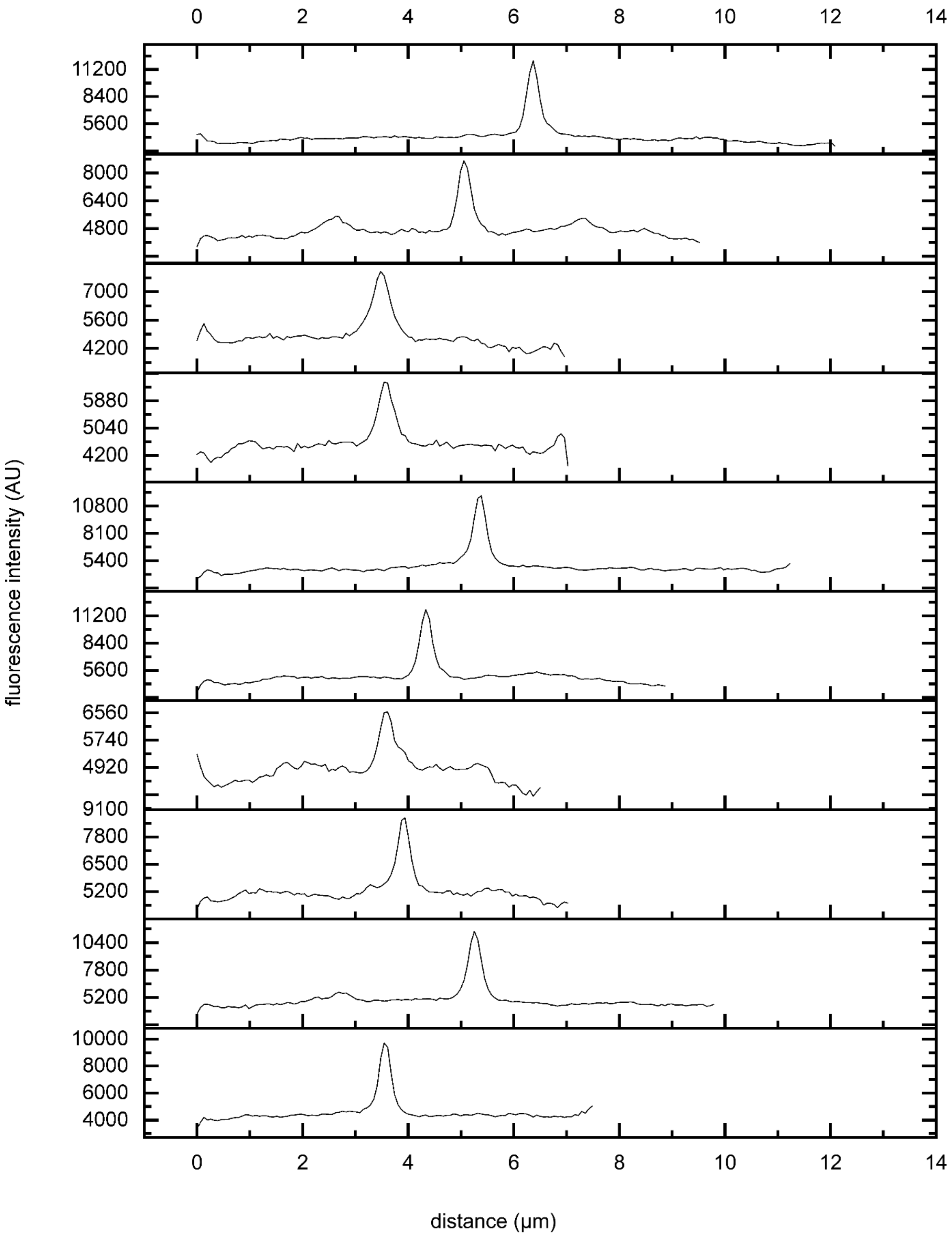

Figure S30: Longitudinal line scans of individual *B. subtilis* PH1 (Δ*ytrB*) cells grown at 24 °C and stained with Van-FL in exponential growth phase.

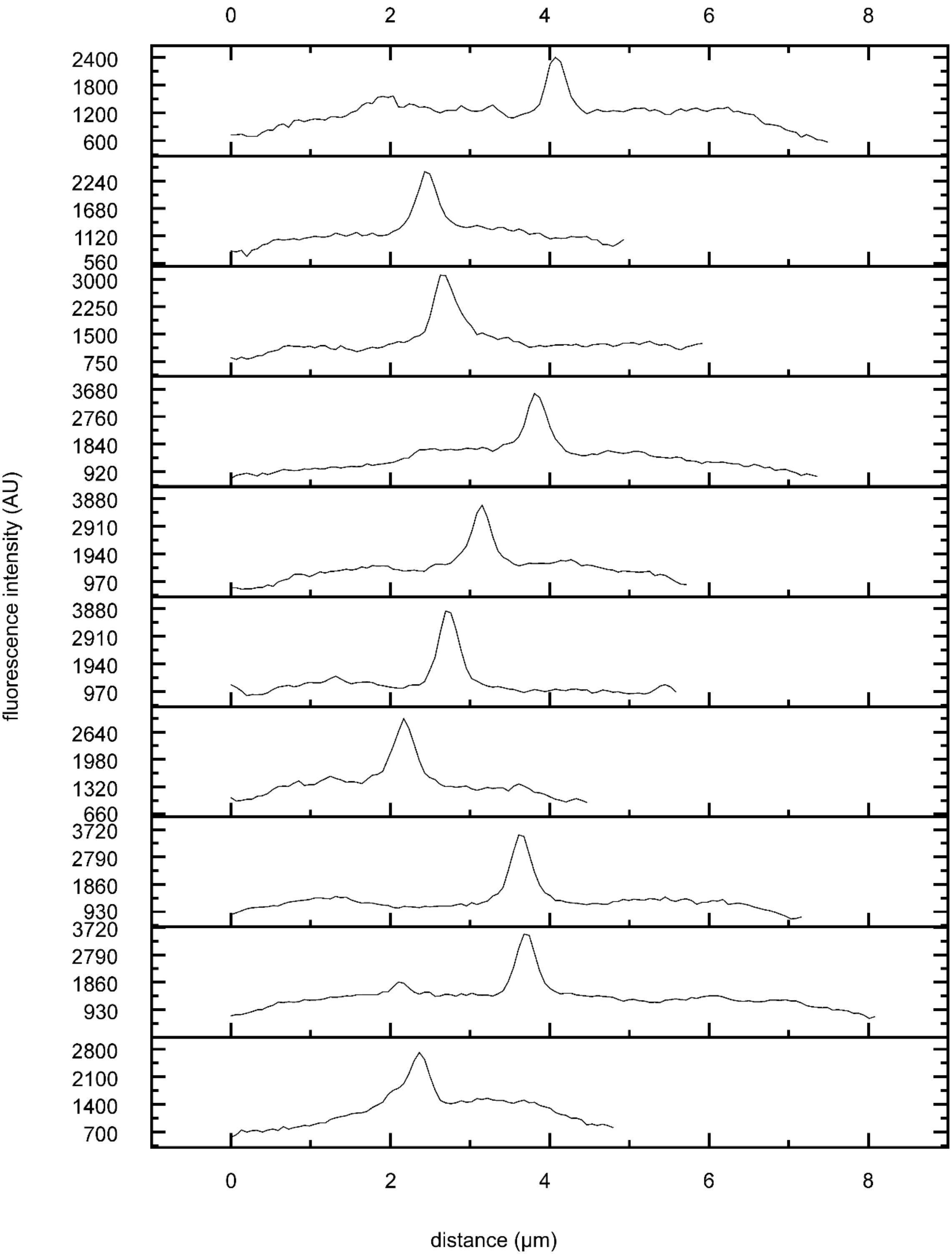

Figure S31: Longitudinal line scans of individual *B. subtilis* PD3 (Δ*ytrC*) cells grown at 24 °C and stained with Van-FL in exponential growth phase.

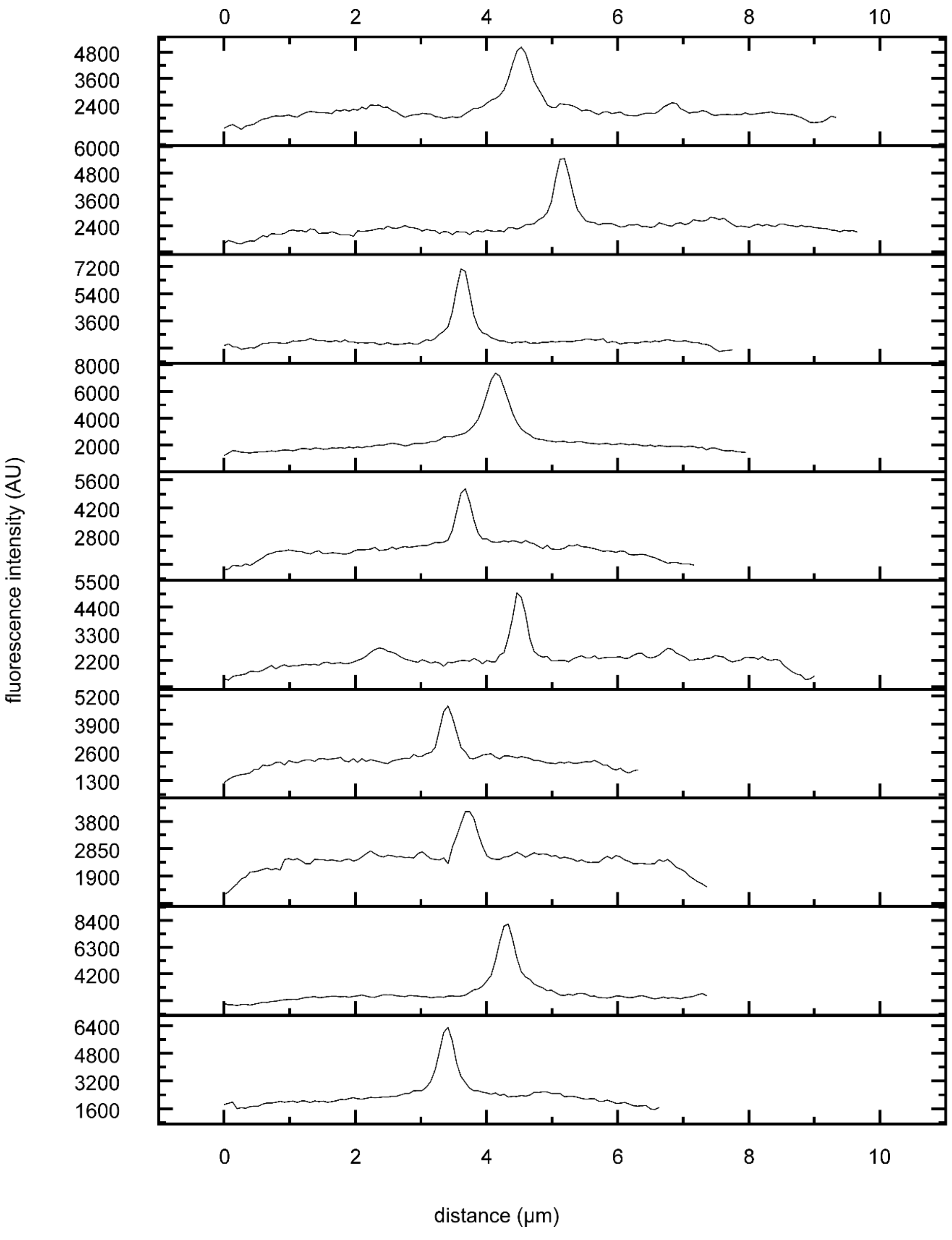

Figure S32: Longitudinal line scans of individual *B. subtilis* PD2 (Δ*ytrD*) cells grown at 24 °C and stained with Van-FL in exponential growth phase.

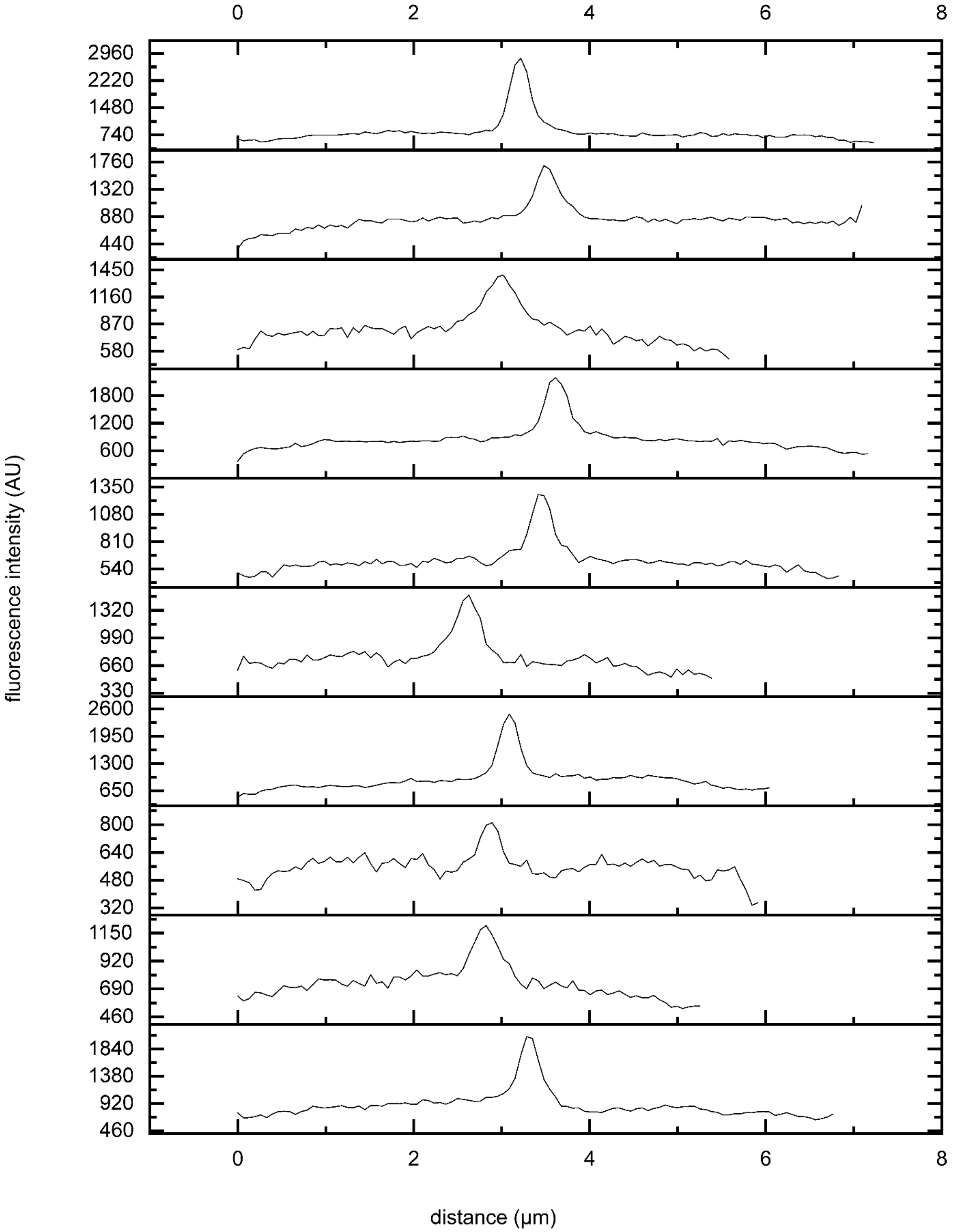

Figure S33: Longitudinal line scans of individual *B. subtilis* PH2 (Δ*ytrE*) cells grown at 24 °C and stained with Van-FL in exponential growth phase.

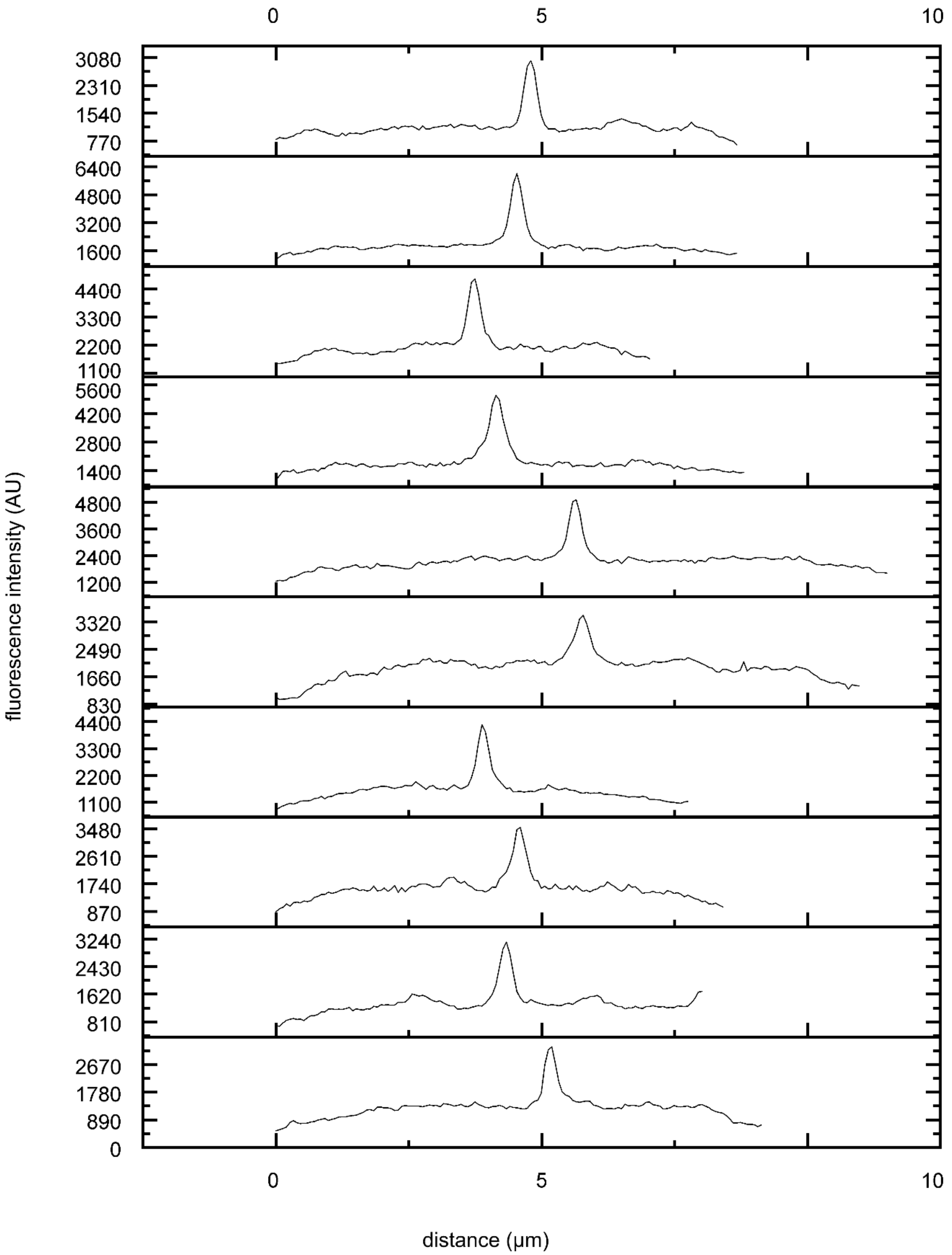

Figure S34: Longitudinal line scans of individual *B. subtilis* PD1 (Δ*ytrF*) cells grown at 24 °C and stained with Van-FL in exponential growth phase.

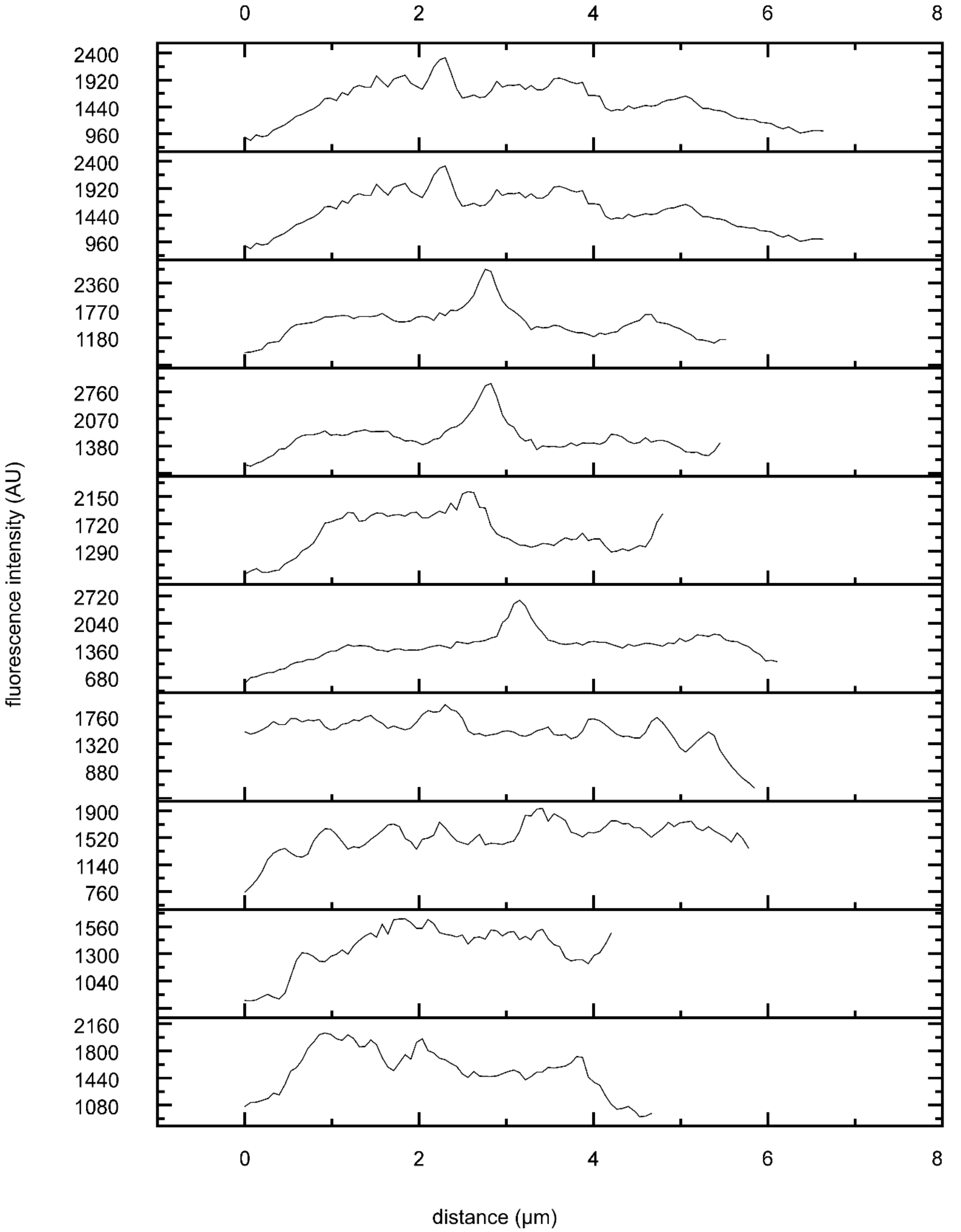

Figure S35: Longitudinal line scans of individual *B. subtilis* GP3193 (Δ*ytrAB*) cells grown at 24 °C and stained with Van-FL in exponential growth phase.

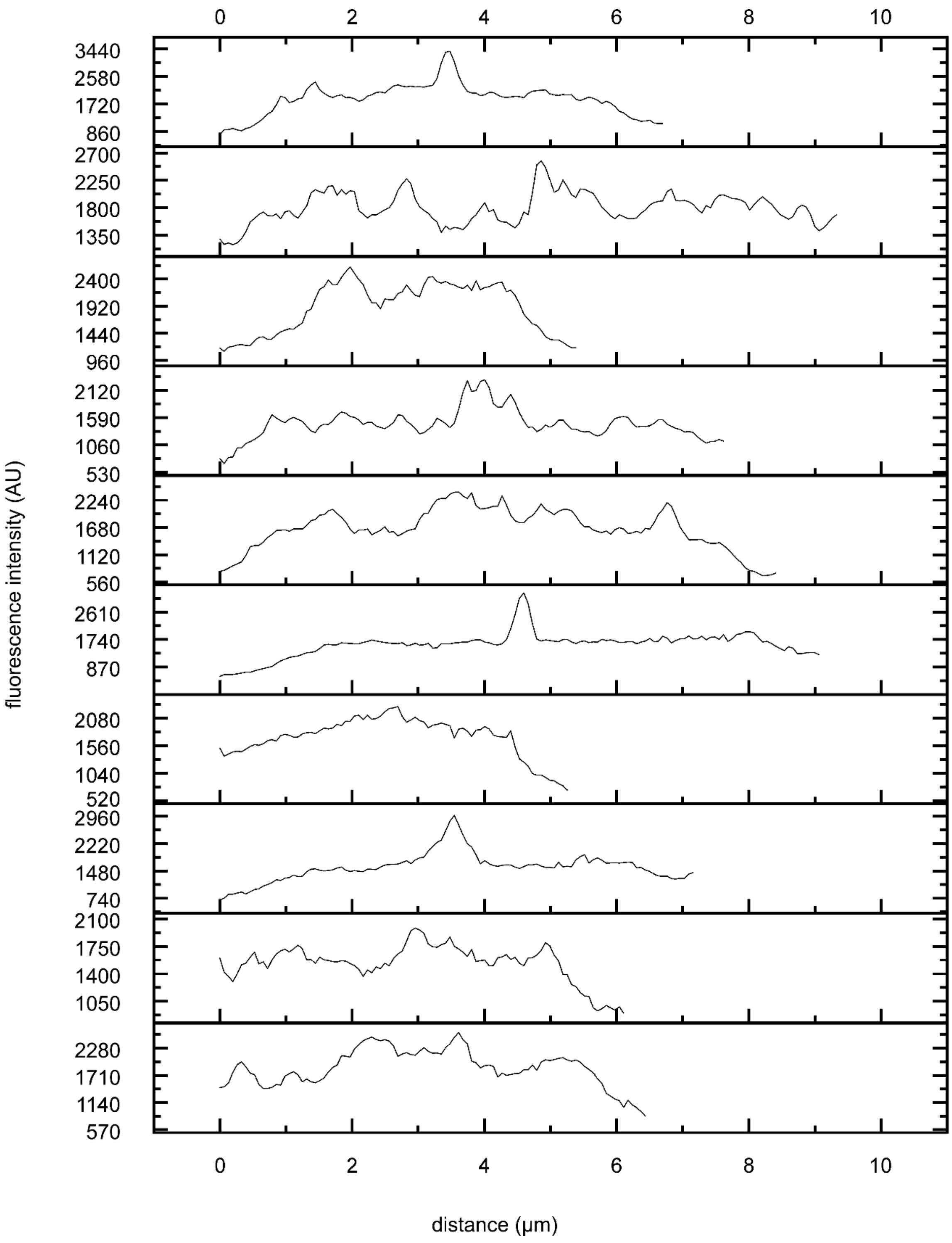

Figure S36: Longitudinal line scans of individual *B. subtilis* GP3196 (Δ*ytrAE*) cells grown at 24 °C and stained with Van-FL in exponential growth phase.

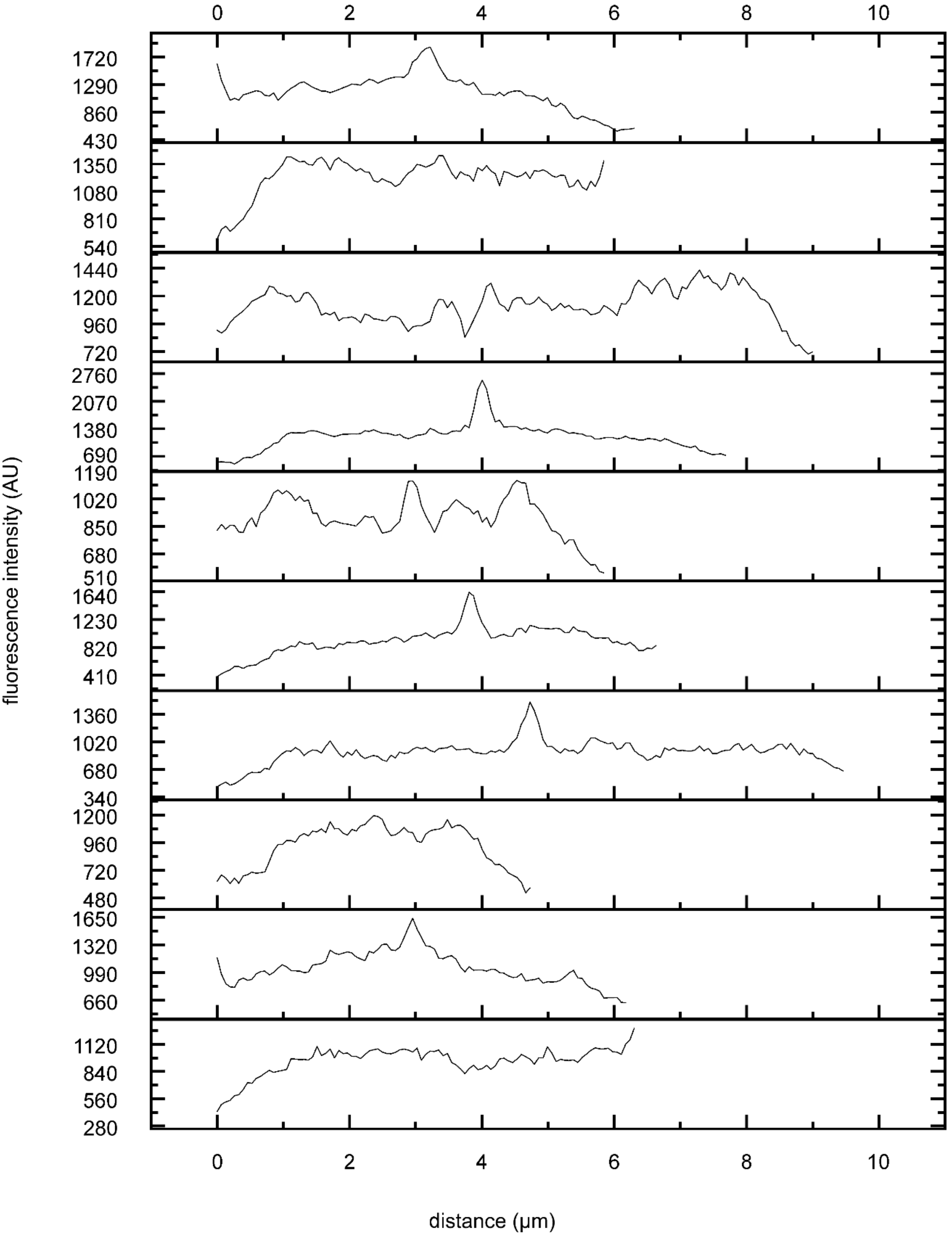

Figure S37: Longitudinal line scans of individual *B. subtilis* GP3206 (Δ*ytrABE*) cells grown at 24 °C and stained with Van-FL in exponential growth phase.

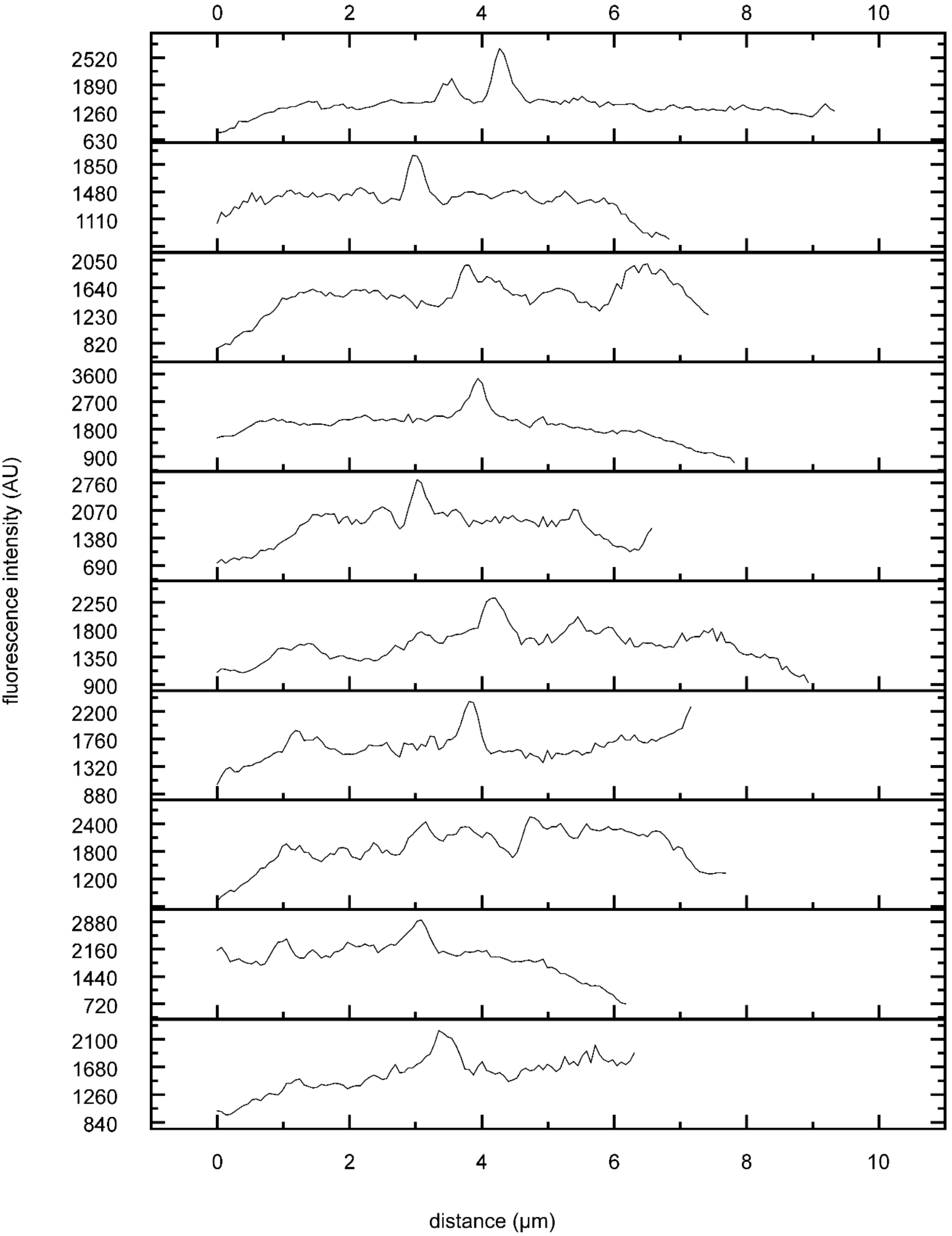

Figure S38: Longitudinal line scans of individual *B. subtilis* BLMS3 (Δ*ytrACD*) cells grown at 24 °C and stained with Van-FL in exponential growth phase.

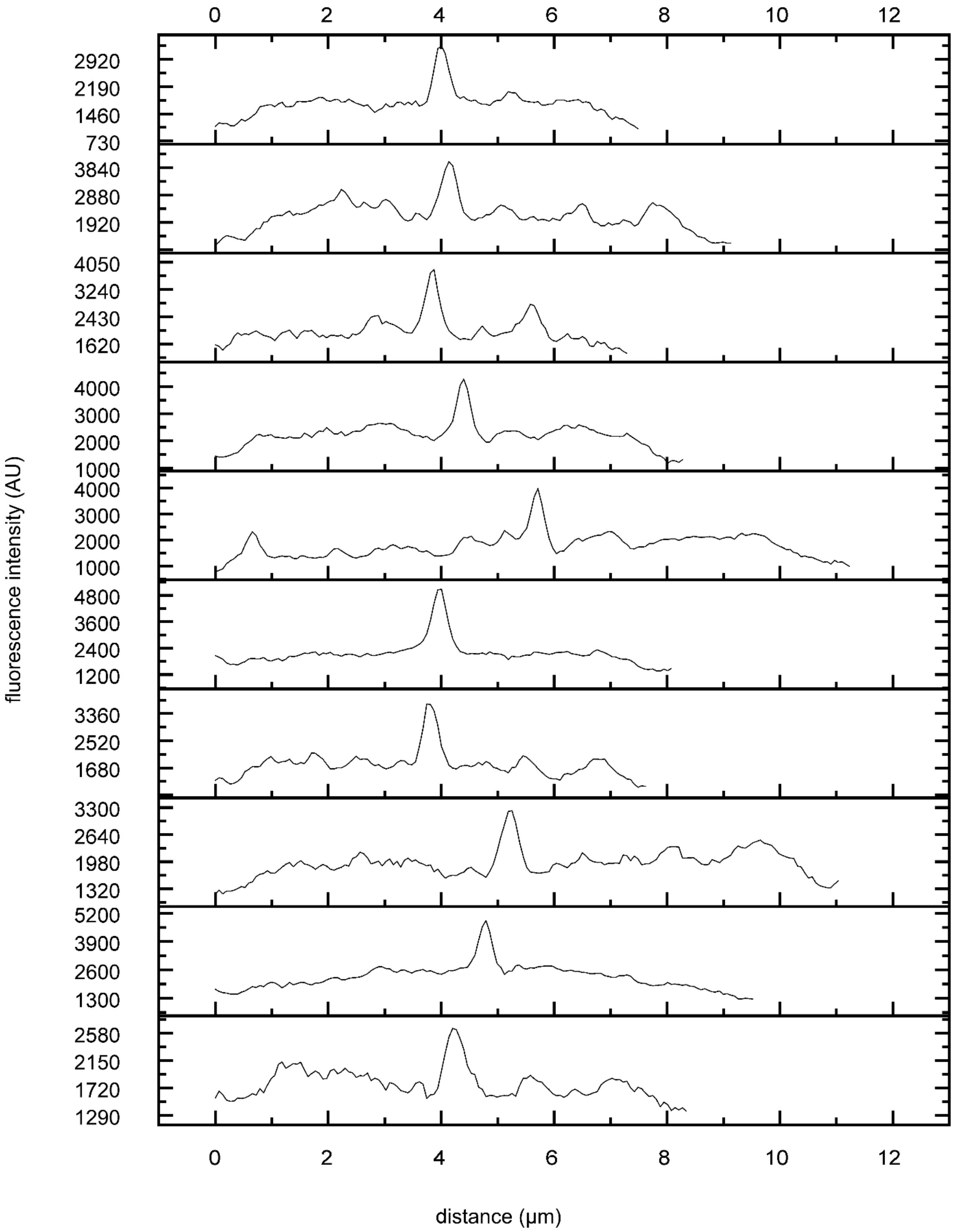

Figure S39: Longitudinal line scans of individual *B. subtilis* GP2646 (Δ*ytrGABCDEF*) cells grown at 24 °C and stained with Van-FL in exponential growth phase.

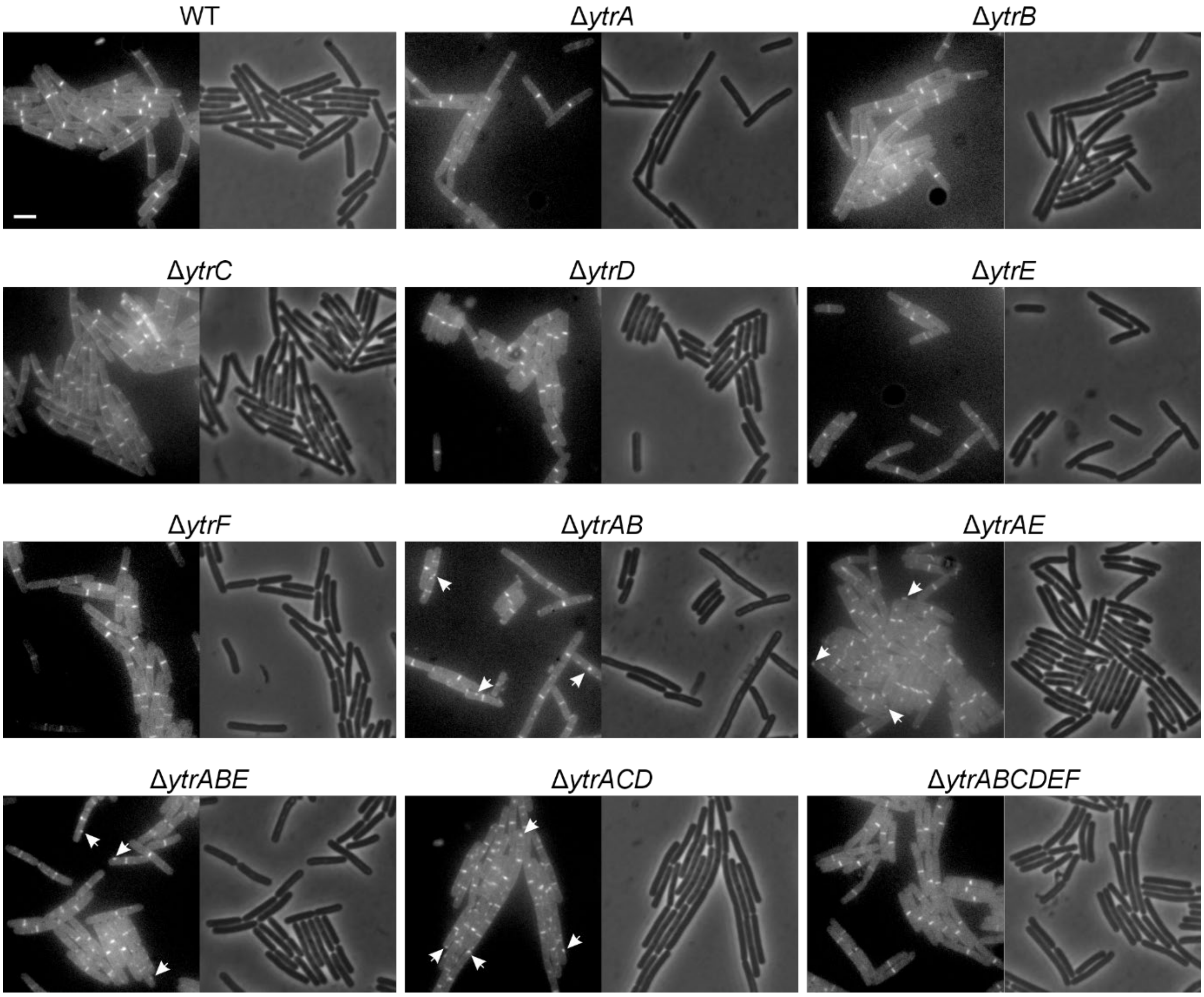

Figure S40: Microscopy images of *B. subtilis* 168CA (WT) and *ytr* mutants grown at 37 °C and labeled with bocillin. Exposure times, light intensity, and brightness/contrast settings were identical for all samples.

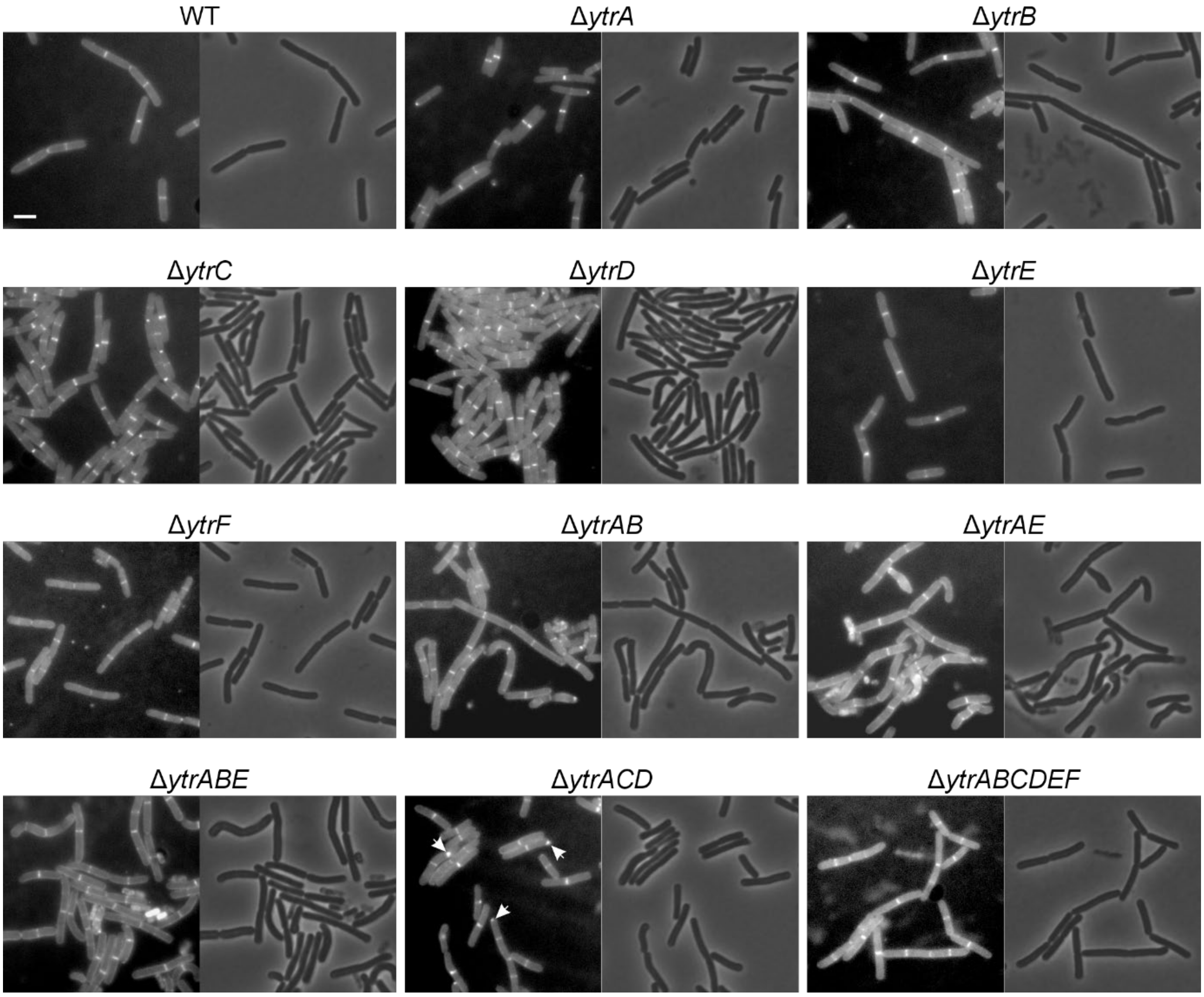

Figure S41: Microscopy images of *B. subtilis* 168CA (WT) and *ytr* mutants grown at 24 °C and labeled with bocillin. Exposure times, light intensity, and brightness/contrast settings were identical for all samples.

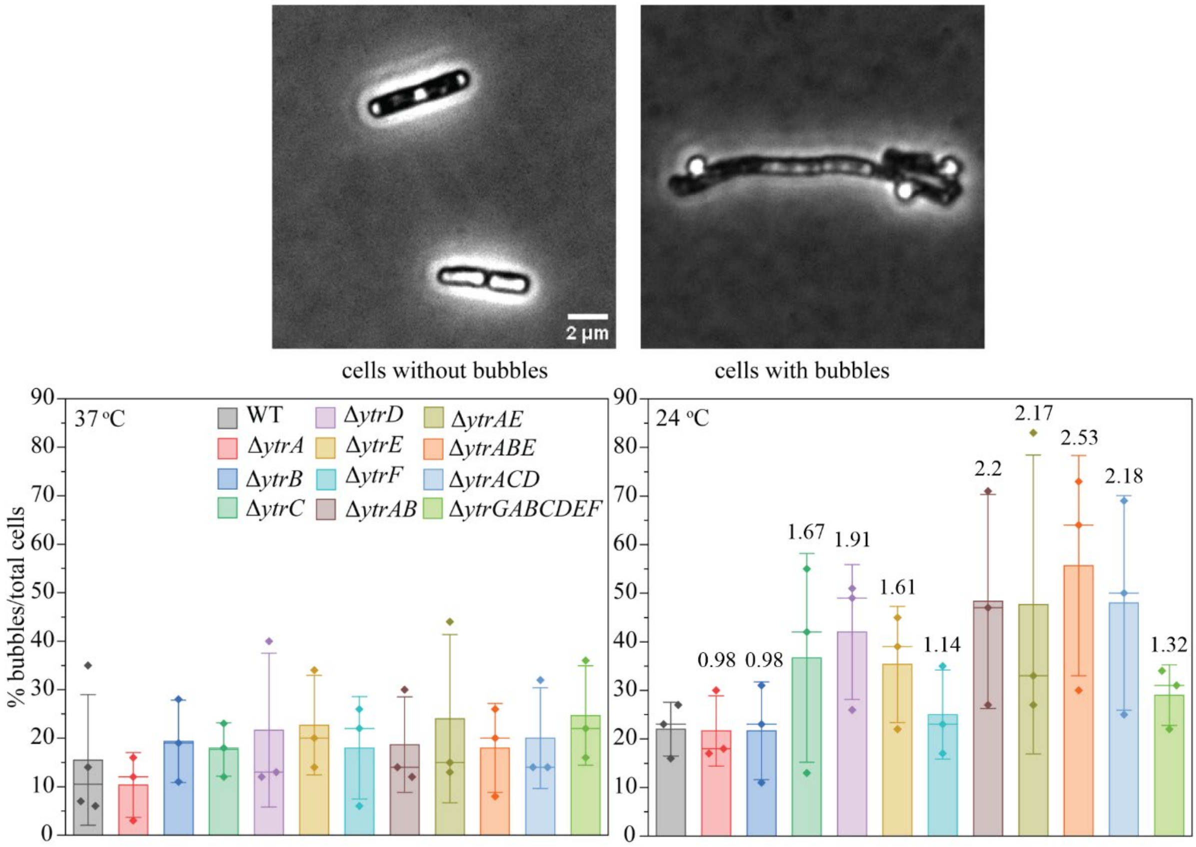

Figure S42: Bubble assay of deletion mutants at 37 °C and 24 °C. Cells were grown until early log phase prior to fixation in 1:3 acetic acid/methanol. Statistical significance was tested with a two-tailed heteroscedastic t-test and no strain showed significant ($p<0.05$) differences from the wild type. Numbers in the 24 °C panel show fold changes compared to the WT.

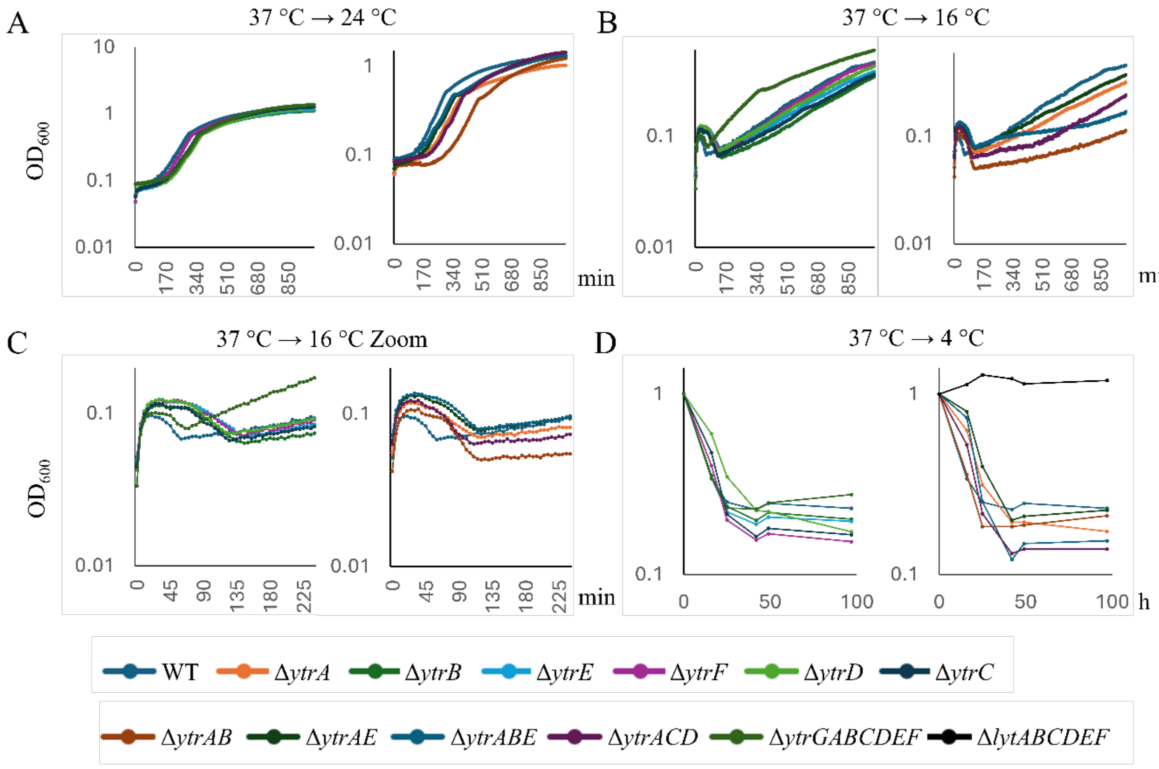

Figure S43: Growth curves of *B. subtilis* 168CA and *ytr* mutants after temperature shifts depicted on log scales. Cultures were shifted from 37 to 24 **(A)**, 16 **(B-C)**, or 4 °C **(D)** after dilution of overnight cultures. For the 4 °C experiment in **(D)**, the autolysin-defective Δ*lytABCDEF* mutant was included as negative control. Left panels show strains not constitutively expressing the operon (Δ*ytrB*, Δ*ytrC*, Δ*ytrD*, Δ*ytrE*, Δ*ytrF*, Δ*ytrGABCDEF*), while the right panels show strains that force-express the whole operon or parts thereof (Δ*ytrA*, Δ*ytrAB*, Δ*ytrAE*, Δ*ytrABE*, Δ*ytrACD*) and, in **(D)**, the Δ*lytABCDEF* control.

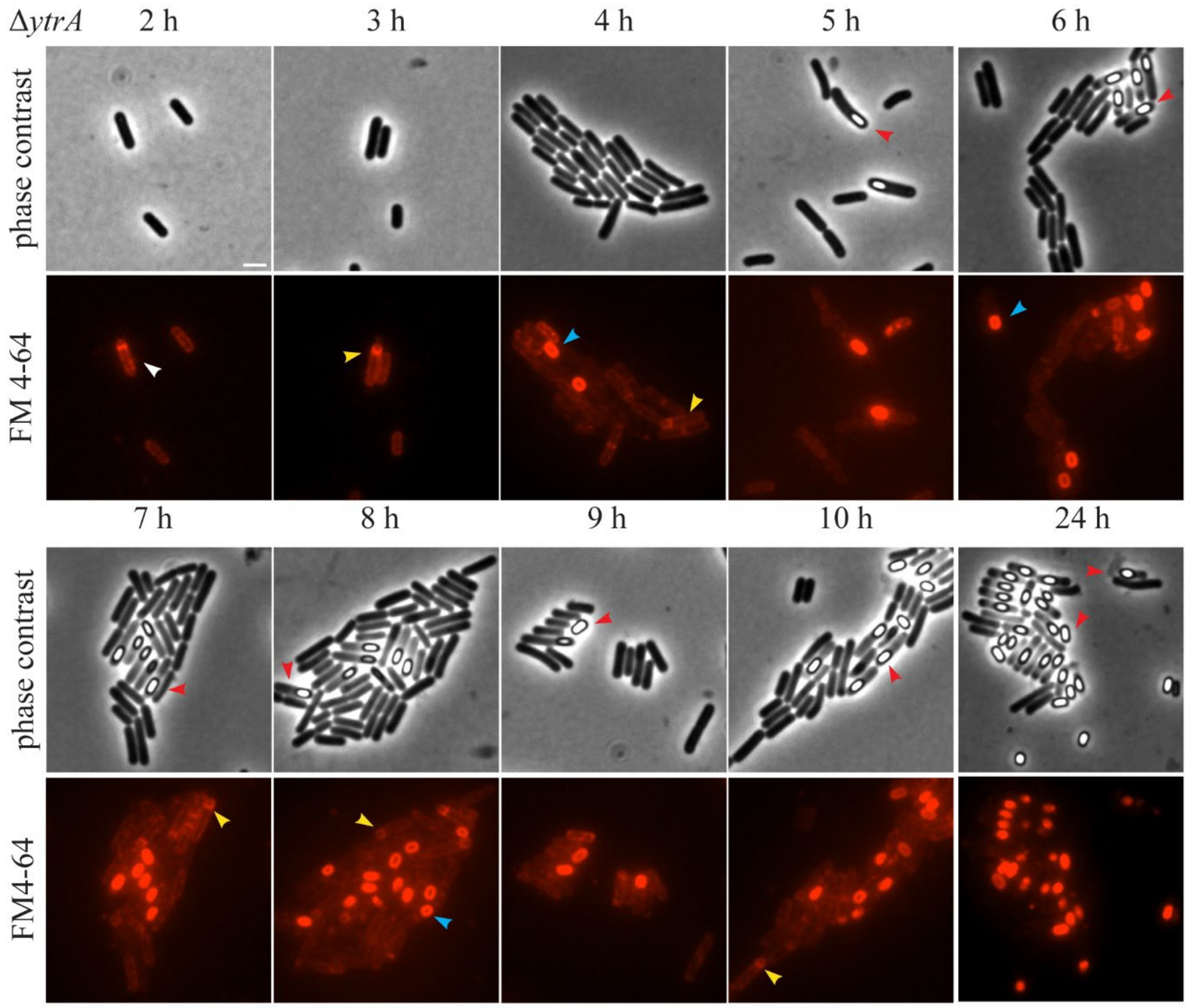

Figure S44: Fluorescence and phase contrast microscopy image of sporulating *B. subtilis* PH5 (*ΔytrA*). Cells were taken for microscopy in hourly intervals from 2-10 h and at 24 h after sporulation induction. The experiment was conducted at 37 °C. The sporulation stage was determined based on FM4-64 membrane staining and phase contrast images. Arrows indicate different stages of sporulation: white: asymmetric septation, yellow: engulfment, blue: forespore stage, red: phase-bright spores. Scale bar 2 µm.

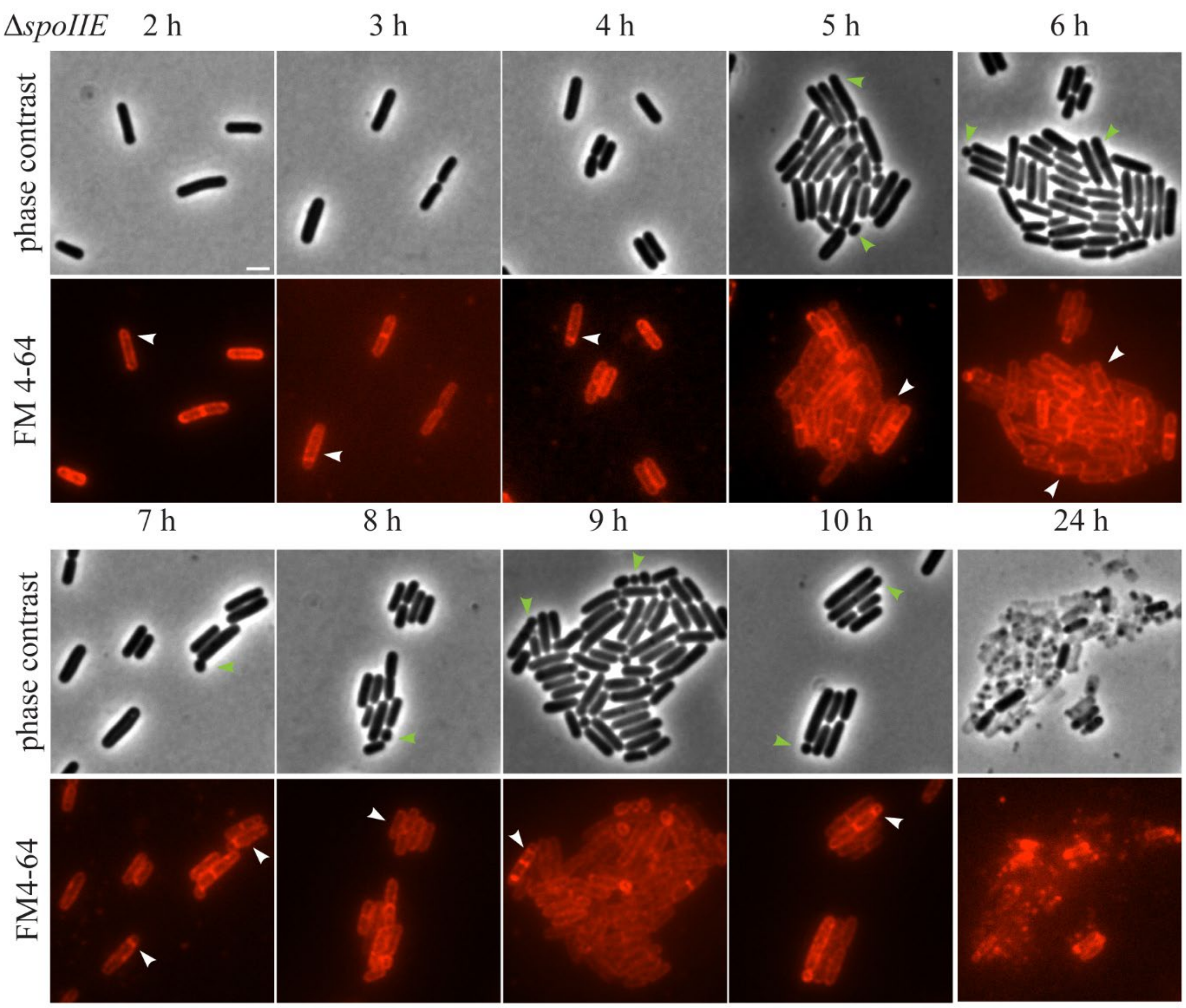

Figure S45: Fluorescence and phase contrast microscopy image of *B. subtilis* PG344 (*ΔspoIIE*). Cells were taken for microscopy in hourly intervals from 2-10 h and at 24 h after sporulation induction. The experiment was conducted at 37 °C. The sporulation stage was determined based on FM4-64 membrane staining and phase contrast images. White arrows indicate asymmetric septation. Green arrows indicate deformed cells. Scale bar 2 µm.

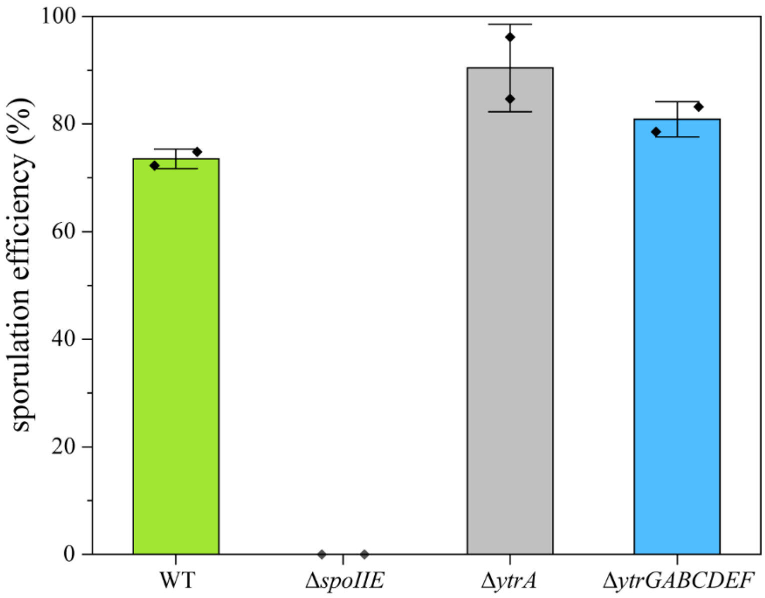

Figure S46: Sporulation efficiency of *B. subtilis* 168CA (wild type), PG344 (*ΔspoIIE*), PH5 (*ΔytrA*), and GP2646 (*ΔytrGABCDEF*). Sporulation efficiency was calculated by dividing the number of colonies growing on LB agar plates of heated (spores) and unheated (total cells) samples. Samples were taken 24 h after sporulation induction. The experiment was conducted at 37 °C. Error bars show standard deviation of the mean of two independent experiments.

References

(1) Wenzel, M.; Patra, M.; Albrecht, D.; Chen, D. Y. K.; Nicolaou, K. C.; Metzler-Nolte, N.; Bandow, J. E. Proteomic signature of fatty acid biosynthesis inhibition available for *in vivo* mechanism-of-action studies. *Antimicrob. Agents Chemother.* **2011**, *55* (6), 2590–2596. https://doi.org/10.1128/AAC.00078-11.

(2) Bandow, J. E.; Brötz, H.; Leichert, L. I. O.; Labischinski, H.; Hecker, M.; Brotz, H.; Leichert, L. I. O.; Labischinski, H.; Hecker, M. Proteomic approach to understanding antibiotic action. *Antimicrob. Agents Chemother.* **2003**, *47* (3), 948–955. https://doi.org/10.1128/AAC.47.3.948.

(3) Stepanek, J. J.; Lukezic, T.; Teichert, I.; Petkovic, H.; Bandow, J. E. Dual mechanism of action of the atypical tetracycline chelocardin. *Biochim. Biophys. Acta* **2016**, *1864* (6), 645–654. https://doi.org/10.1016/j.bbapap.2016.03.004.

(4) Senges, C. H. R.; Stepanek, J. J.; Wenzel, M.; Raatschen, N.; Ay, Ü.; Märtens, Y.; Prochnow, P.; Vázquez Hernández, M.; Yayci, A.; Schubert, B.; Janzing, N. B. M.; Warmuth, H. L.; Kozik, M.; Bongard, J.; Alumasa, J. N.; Albada, B.; Penkova, M.; Lukežič, T.; Sorto, N. A.; Lorenz, N.; Miller, R.; Zhu, B.; Benda, M.; Stülke, J.; Schäkermann, S.; Leichert, L. I.; Scheinpflug, K.; Brötz-Oesterhelt, H.; Hertweck, C.; Shaw, J. T.; Petković, H.; Brunel, J. M.; Keiler, K. C.; Metzler-Nolte, N.; Bandow, J. E. Comparison of proteomic responses as global approach to antibiotic mechanism of action elucidation. *Antimicrob. Agents Chemother.* **2020**, *65* (1), e01373-20. https://doi.org/10.1128/AAC.01373-20.

(5) Müller, A.; Wenzel, M.; Strahl, H.; Grein, F.; Saaki, T. N. V.; Kohl, B.; Siersma, T.; Bandow, J. E.; Sahl, H.-G.; Schneider, T.; Hamoen, L. W. Daptomycin inhibits cell envelope synthesis by interfering with fluid membrane microdomains. *Proc. Natl. Acad. Sci.* **2016**, *113* (45). https://doi.org/10.1073/pnas.1611173113.

(6) Wenzel, M.; Kohl, B.; Münch, D.; Raatschen, N.; Albada, H. B.; Hamoen, L.; Metzler-Nolte, N.; Sahl, H. G.; Bandow, J. E. Proteomic response of *Bacillus subtilis* to lantibiotics reflects differences in interaction with the cytoplasmic membrane. *Antimicrob. Agents Chemother.* **2012**, *56* (11), 5749–5757. https://doi.org/10.1128/AAC.01380-12.

(7) Wenzel, M.; Chiriac, A. I.; Otto, A.; Zweytick, D.; May, C.; Schumacher, C.; Gust, R.; Albada, H. B.; Penkova, M.; Krämer, U.; Erdmann, R.; Metzler-Nolte, N.; Straus, S. K.; Bremer, E.; Becher, D.; Brötz-Oesterhelt, H.; Sahl, H.-G.; Bandow, J. E. Small cationic antimicrobial peptides delocalize peripheral membrane proteins. *Proc. Natl. Acad. Sci.* **2014**, *111* (14). https://doi.org/10.1073/pnas.1319900111.

(8) Münch, D.; Müller, A.; Schneider, T.; Kohl, B.; Wenzel, M.; Bandow, J. E.; Maffioli, S.; Sosio, M.; Donadio, S.; Wimmer, R.; Sahl, H.-G. G.; Münch, D.; Müller, A.; Schneider, T.; Kohl, B.; Wenzel, M.; Bandow, J. E.; Maffioli, S.; Sosio, M.; Donadio, S.; Wimmer, R.; Sahl, H.-G. G.; Munch, D.; Muller, A.; Schneider, T.; Kohl, B.; Wenzel, M.; Bandow, J. E.; Maffioli, S.; Sosio, M.; Donadio, S.; Wimmer, R.; Sahl, H.-G. G. The lantibiotic NAI-107 binds to bactoprenol-bound cell wall precursors and impairs membrane functions. *J. Biol. Chem.* **2014**, *289* (17), 12063–12076. https://doi.org/10.1074/jbc.M113.537449.

(9) Salzberg, L. I.; Luo, Y.; Hachmann, A.-B.; Mascher, T.; Helmann, J. D. The *Bacillus subtilis* GntR family repressor YtrA responds to cell wall antibiotics. *J. Bacteriol.* **2011**, *193* (20), 5793–5801. https://doi.org/10.1128/JB.05862-11.

(10) Bachmann, B. J. Derivations and genotypes of some mutant derivatives of *Escherichia coli* K-12. In *Escherichia coli and Salmonella: cellular and molecular biology*; Neidhardt, F.C.; Ingraham, J.L.; Low, K.B.; Magasanik, B.; Schaechter, M.;

Umbarger, H. E., Ed.; American Society for Microbiology: Washington, D.C., 1987; pp 1190–1219.
(11) Spizizen, J. Transformation of biochemically deficient srains of *Bacillus subtilis* by deoxyribonucleate. *Proc. Natl. Acad. Sci.* **1958**, *44* (10), 1072–1078. https://doi.org/10.1073/pnas.44.10.1072.
(12) Koo, B.-M.; Kritikos, G.; Farelli, J. D.; Todor, H.; Tong, K.; Kimsey, H.; Wapinski, I.; Galardini, M.; Cabal, A.; Peters, J. M.; Hachmann, A.-B.; Rudner, D. Z.; Allen, K. N.; Typas, A.; Gross, C. A. Construction and analysis of two genome-scale deletion libraries for *Bacillus subtilis*. *Cell Syst.* **2017**, *4* (3), 291-305.e7. https://doi.org/10.1016/j.cels.2016.12.013.
(13) Benda, M.; Schulz, L. M.; Stülke, J.; Rismondo, J. Influence of the ABC transporter YtrBCDEF of *Bacillus subtilis* on competence, biofilm formation and cell wall thickness. *Front. Microbiol.* **2021**, *12*, 587035. https://doi.org/10.3389/fmicb.2021.587035.
(14) Scheinpflug, K.; Wenzel, M.; Krylova, O.; Bandow; E, J.; Dathe, M.; Strahl, H. Antimicrobial peptide CWFW kills by combining lipid phase separation with autolysis. *Sci. Rep.* **2017**, *7*, 44332. https://doi.org/10.1038/srep44332.
(15) Gray, D. A.; Dugar, G.; Gamba, P.; Strahl, H.; Jonker, M. J.; Hamoen, L. W. Extreme slow growth as alternative strategy to survive deep starvation in bacteria. *Nat. Commun.* **2019**, *10* (1), 890. https://doi.org/10.1038/s41467-019-08719-8.
(16) Rafailidis, P. I.; Ioannidou, E. N.; Falagas, M. E. Ampicillin/sulbactam: current status in severe bacterial infections. *Drugs* **2007**, *67* (13), 1829–1849. https://doi.org/10.2165/00003495-200767130-00003.
(17) Scherer, K. M.; Spille, J. H.; Sahl, H. G.; Grein, F.; Kubitscheck, U. The lantibiotic nisin induces lipid ii aggregation, causing membrane instability and vesicle budding. *Biophys. J.* **2015**, *108* (5), 1114–1124. https://doi.org/10.1016/j.bpj.2015.01.020.
(18) Koch, D. C.; Schmidt, T. H.; Sahl, H.-G.; Kubitscheck, U.; Kandt, C. Structural Dynamics of the cell wall precursor lipid II in the presence and absence of the lantibiotic nisin. *Biochim. Biophys. Acta* **2014**, *1838* (12), 3061–3068. https://doi.org/10.1016/j.bbamem.2014.07.024.
(19) Wiedemann, I.; Breukink, E.; van Kraaij, C.; Kuipers, O. P.; Bierbaum, G.; de Kruijff, B.; Sahl, H.-G. Specific binding of nisin to the peptidoglycan precursor lipid II combines pore formation and inhibition of cell wall biosynthesis for potent antibiotic activity. *J. Biol. Chem.* **2001**, *276* (3), 1772–1779. https://doi.org/10.1074/jbc.M006770200.
(20) Grein, F.; Schneider, T.; Sahl, H.-G. Docking on Lipid II-a widespread mechanism for potent bactericidal activities of antibiotic peptides. *J. Mol. Biol.* **2019**, *431* (18), 3520–3530. https://doi.org/10.1016/j.jmb.2019.05.014.
(21) Qiao, Y.; Srisuknimit, V.; Rubino, F.; Schaefer, K.; Ruiz, N.; Walker, S.; Kahne, D. Lipid II overproduction allows direct assay of transpeptidase inhibition by β-lactams. *Nat. Chem. Biol.* **2017**, *13* (7), 793–798. https://doi.org/10.1038/nchembio.2388.
(22) Prosser, G. A.; de Carvalho, L. P. S. Kinetic mechanism and inhibition of *Mycobacterium tuberculosis* D -alanine: D -alanine ligase by the antibiotic D - cycloserine. *FEBS J.* **2013**, *280* (4), 1150–1166. https://doi.org/10.1111/febs.12108.
(23) Champney, W. S.; Miller, M. Inhibition of 50S ribosomal subunit assembly in *Haemophilus influenzae* cells by azithromycin and erythromycin. *Curr. Microbiol.* **2002**, *44* (6), 418–424. https://doi.org/10.1007/s00284-001-0016-6.
(24) Chopra, I.; Hawkey, P. M.; Hinton, M. Tetracyclines, molecular and clinical aspects. *J. Antimicrob. Chemother.* **1992**, *29* (3), 245–277. https://doi.org/10.1093/jac/29.3.245.
(25) Schnappinger, D.; Hillen, W. Tetracyclines: antibiotic action, uptake, and resistance

mechanisms. *Arch. Microbiol.* **1996**, *165* (6), 359–369.
(26) Strahl, H.; Hamoen, L. W. Membrane potential is important for bacterial cell division. *Proc. Natl. Acad. Sci. U. S. A.* **2010**, *107* (27), 12281–12286. https://doi.org/10.1073/pnas.1005485107.
(27) Tempelaars, M. H.; Rodrigues, S.; Abee, T. Comparative analysis of antimicrobial activities of valinomycin and cereulide, the *Bacillus cereus* emetic toxin. *Appl. Environ. Microbiol.* **2011**, *77* (8), 2755–2762. https://doi.org/10.1128/AEM.02671-10.
(28) Nicolas, P.; Mäder, U.; Dervyn, E.; Rochat, T.; Leduc, A.; Pigeonneau, N.; Bidnenko, E.; Marchadier, E.; Hoebeke, M.; Aymerich, S.; Becher, D.; Bisicchia, P.; Botella, E.; Delumeau, O.; Doherty, G.; Denham, E. L.; Fogg, M. J.; Fromion, V.; Goelzer, A.; Hansen, A.; Härtig, E.; Harwood, C. R.; Homuth, G.; Jarmer, H.; Jules, M.; Klipp, E.; Le Chat, L.; Lecointe, F.; Lewis, P.; Liebermeister, W.; March, A.; Mars, R. A. T.; Nannapaneni, P.; Noone, D.; Pohl, S.; Rinn, B.; Rügheimer, F.; Sappa, P. K.; Samson, F.; Schaffer, M.; Schwikowski, B.; Steil, L.; Stülke, J.; Wiegert, T.; Devine, K. M.; Wilkinson, A. J.; van Dijl, J. M.; Hecker, M.; Völker, U.; Bessières, P.; Noirot, P. Condition-dependent transcriptome reveals high-level regulatory architecture in *Bacillus subtilis*. *Science* **2012**, *335* (6072), 1103–1106. https://doi.org/10.1126/science.1206848.
(29) Pedreira, T.; Elfmann, C.; Stülke, J. The current state of SubtiWiki, the database for the model organism *Bacillus subtilis*. *Nucleic Acids Res.* **2022**, *50* (D1), D875–D882. https://doi.org/10.1093/nar/gkab943.
